# COVID‐19: A systematic review and update on prevention, diagnosis, and treatment

**DOI:** 10.1002/mco2.115

**Published:** 2022-02-17

**Authors:** Hooman Aghamirza Moghim Aliabadi, Reza Eivazzadeh‐Keihan, Arezoo Beig Parikhani, Sara Fattahi Mehraban, Ali Maleki, Sepideh Fereshteh, Masoume Bazaz, Ashkan Zolriasatein, Bahareh Bozorgnia, Saman Rahmati, Fatemeh Saberi, Zeinab Yousefi Najafabadi, Shadi Damough, Sara Mohseni, Hamid Salehzadeh, Vahid Khakyzadeh, Hamid Madanchi, Gholam Ali Kardar, Payam Zarrintaj, Mohammad Reza Saeb, Masoud Mozafari

**Affiliations:** ^1^ Protein Chemistry Laboratory Department of Medical Biotechnology Biotechnology Research Center Pasteur Institute of Iran Tehran Iran; ^2^ Advance Chemical Studies Laboratory Faculty of Chemistry K. N. Toosi University Tehran Iran; ^3^ Department of Chemistry Iran University of Science and Technology Tehran Iran; ^4^ Department of Medical Biotechnology Biotechnology Research Center Pasteur Institute Tehran Iran; ^5^ Non‐metallic Materials Research Group Niroo Research Institute Tehran Iran; ^6^ Department of Bacteriology Pasteur Institute of Iran Tehran Iran; ^7^ Faculty of Chemistry Alzahra University Tehran Iran; ^8^ Department of Medical Biotechnology School of Advanced Technologies in Medicine Shahid Beheshti University of Medical Sciences Tehran Iran; ^9^ Department of Medical Biotechnology School of Advanced Technologies in Medicine Tehran University of Medical Sciences Tehran Iran; ^10^ Immunology Asthma & Allergy Research Institute Tehran University of Medical Sciences Tehran Iran; ^11^ Faculty of Chemistry Kharazmi University Tehran Iran; ^12^ Department of Chemistry K. N. Toosi University of Technology Tehran Iran; ^13^ School of Medicine Semnan University of Medical Sciences Semnan Iran; ^14^ Drug Design and Bioinformatics Unit Department of Medical Biotechnology Biotechnology Research Center Pasteur Institute of Iran Tehran Iran; ^15^ School of Chemical Engineering Oklahoma State University Stillwater Oklahoma USA; ^16^ Department of Polymer Technology Faculty of Chemistry Gdańsk University of Technology Gdańsk Poland; ^17^ Department of Tissue Engineering & Regenerative Medicine Iran University of Medical Sciences Tehran Iran

**Keywords:** biomaterials, coronavirus, COVID‐19, nanotechnology, pandemic, SARS‐CoV‐2

## Abstract

Since the rapid onset of the COVID‐19 or SARS‐CoV‐2 pandemic in the world in 2019, extensive studies have been conducted to unveil the behavior and emission pattern of the virus in order to determine the best ways to diagnosis of virus and thereof formulate effective drugs or vaccines to combat the disease. The emergence of novel diagnostic and therapeutic techniques considering the multiplicity of reports from one side and contradictions in assessments from the other side necessitates instantaneous updates on the progress of clinical investigations. There is also growing public anxiety from time to time mutation of COVID‐19, as reflected in considerable mortality and transmission, respectively, from delta and Omicron variants. We comprehensively review and summarize different aspects of prevention, diagnosis, and treatment of COVID‐19. First, biological characteristics of COVID‐19 were explained from diagnosis standpoint. Thereafter, the preclinical animal models of COVID‐19 were discussed to frame the symptoms and clinical effects of COVID‐19 from patient to patient with treatment strategies and in‐silico/computational biology. Finally, the opportunities and challenges of nanoscience/nanotechnology in identification, diagnosis, and treatment of COVID‐19 were discussed. This review covers almost all SARS‐CoV‐2‐related topics extensively to deepen the understanding of the latest achievements (last updated on January 11, 2022).

## Graphical abstract

This review covers almost all topics related to SARS‐CoV‐2, including biological properties, pre‐clinical studies, clinical information, diagnostic and treatment methods, and in silico studies, which is very useful for a quick understanding of the latest advances in this field. Also, in a separate section, the applications of nanotechnology in COVID‐19 studies are examined.

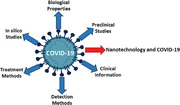

## 1 INTRODUCTION AND BACKGROUND

Based on the World Health Organization (WHO) declaration, the total reported cases of the ongoing global pandemic, coronavirus disease 2019 (COVID‐19), naming Severe Acute Respiratory Syndrome Coronavirus 2 (SARS‐CoV‐2) via the International Committee on Taxonomy of Viruses (ICTV), as of 5:48 pm CEST, January 11, 2022, have been more than 311,316,780 confirmed cases with 5,514,603 deaths, with a very sharp increase in the number of new cases because of accelerated Omicron variant transmission (designated by WHO on November 26, 2021) in the world.^1^ This new emerging zoonotic reservoir virus with one or more probable mammal intermediate hosts has received the human transmission ability like the other six Coronaviruses family members, especially alpha and beta subfamilies (HCoV‐OC43, HCoV‐NL63, HCoV‐HKU1, HCoV‐229E, MERS‐CoV, SARS‐CoV, and 2019‐nCoV [SARS‐CoV‐2]).^2^ In the 20th century, the three of seven members of Coronaviruses family (SARS‐2003 [case‐fatality rate: 10–12%], MERS‐2012 [case‐fatality rate: 36%], and COVID‐19–2020 [case‐fatality rate: estimated lower than other]) have been the cause of the highest death rate from pneumonia after lower respiratory tract replication. Although COVID‐19 has lower case fatality, its transmissibility was proved to be higher than other respiratory viruses based on R0 value calculation. Age, sex, having risk factors, and country regions can be affected by the case‐fatality rate.^3^ New reports declare the effect of virus genome mutation on the pathogenicity and immunogenicity of viruses.^4^, ^5^ Transmission of the virus can occur through human respiratory droplets or contact with virus‐infected surfaces.^6^ Transmission through blood transfusion, solid organ transplantation, and mother‐to‐neonate vertical transmission have no convincing evidence and need further studies.The possibility of transmitting the virus through environmental factors such as virus‐contaminated wastewater and airborne dust is under investigation. In this situation, the management of transmission by prevention and screening of COVID‐19 is the best advice. The development of high‐powered and automated techniques for virus monitoring is required.^7^, ^8^, ^9^, ^10^, ^11^ Dry cough, sore throat, fatigue, runny nose, and severe headache are the COVID‐19 ordinary symptoms. Possible complications are fever, severe breath shortness, and digestive symptoms, such as diarrhea, severe pneumonia, hemoptysis, and anosmia. Subsequently, multiorgan damage can happen.^12^ Symptoms and case history reviews are the first level of medical detection of the virus. Reverse transcription polymerase chain reaction (RT‐PCR) for disease in its early stages and chest computed tomography (CT) scans for severe stages are confirmable detection ways.^3^

The serological test has not been confirmed yet for the detection of COVID‐19 by medical policymakers. Despite the sensitivity of immunoglobulin M (IgM) and immunoglobulin G (IgG) antibody detection tests being 77% and 83%, and the specificity of them being 100% and 95%, immunoglobulin secretion has not had the same pattern in all cases. To enhance the speed and efficiency of detection, researchers are developing point of care (POC) tests. Potential strategies to combat COVID‐19 include interferon (IFN) therapies, monoclonal antibodies (mAbs), vaccine production, peptides, oligonucleotide‐based therapies, small molecules or natural remedies of conventional medicine, and plasma therapy. The time to develop a *de novo* small molecule drug is more than 6 years, and in the best time, it takes at least 2 years. Vaccines are produced faster, about 1–2 years. Immune system supportive antibodies can be used to treat viral diseases, although the period of their production and development is usually lasting several years.^13^ The promising strategy in the current situation is the new use of old drugs, and its purpose is to discover new fields for previously authorized drugs.^14^ The main benefit of reusing the drug is fast development due to the vast knowledge about this drug's behavior in humans. The basis of the disease is on immunology and genetics, so the role of controversial factors, such as IL6, while affecting severe respiratory symptoms, should not be overlooked in controlling inflammation, virus removal, T cell maturation, and acquired immunity. Therefore, the use of any permitted immunosuppressive agents is not allowed and must be checked.^15^, ^16^ Increasing flu vaccine absorption or strengthening public health interventions may facilitate the management of respiratory outbreaks during the flu season and compensate for the lack of diagnostic resources. However, how to increase the coverage of influenza vaccination remains a challenge. Public health decision‐makers and policymakers need to adopt informed strategies to improve flu vaccination. Difficult months lie ahead for researchers, medical staff, and government rulers.^17^

## 2 BIOLOGICAL PROPERTIES

**FIGURE 1:**
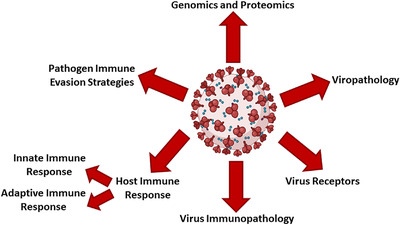
An overview of the biological properties of COVID‐19. The figure is created with BioRender.com

In this section, the biological properties of COVID‐19 referred to in Figure 1 are examined.

### 2.1 Genomics and proteomics

SARS‐CoV‐2 is a 100 nm enveloped virus with about one femtogram mass containing a positive‐sense, linear single‐strand RNA with a length of about 29,800 base pairs. Two‐thirds of the genome typically codes for nonstructural proteins, and a third of the genome encodes structural proteins.^18^ Four main structural proteins, which construct the virus, are membrane (M), envelope (E), nucleocapsid (N), and spike (S) proteins. The spike protein is the most noteworthy antigen on the virus's surface and has a trimmer structure with two subunits called S1 and S2. S1 possesses more important role with more diversity between species. The S2 subunit plays the virus membrane fusion role and is more protected. The receptor‐binding domain (RBD) sequence of the spike S1 subunit, which the virus binds to the receptor through this domain, is the primary and specific target of the virus in producing diagnostic kits and drugs.^19^ There are more than 5000 reports examining the complete genomes of the virus and their mutations. These reports show that 1100 nucleoids have undergone point mutations.^20^ It is reported that there are variants in some virus gene regions, which has changed the level of amino acid residues to about 12 nucleotides. The mutation rate of this virus is low and is about 10^−6^/site/cycle, while the mutation of similar viruses, such as influenza, is 3*10^−6^/site/cycle.^21^ The presence of endonuclease and exonuclease as proofreading enzymes and RNA‐dependent RNA polymerase enzyme (RdRp) are the main reasons for these mutations.^22^ Bat coronavirus (RATG13) and Penguin coronavirus, respectively, with 96% and 91% similarity, have the closest genome structure with SARS‐CoV‐2.^23^, ^24^ There are emerging new variants, which are reported from the beginning of the pandemic till now, which are driven of the heritable mutations of SARS‐CoV‐2.^25^ In Table 1, these variants are listed in order of variant of concern (VOC), variant under monitoring (VUM), and variant of interest (VOI). Jackson and colleagues show evidence of recombination in SARS‐CoV‐2. In their study, four from eight identified recombinant‐origin viruses had onward transmission evidence.^26^

**TABLE 1:** Heritable mutations of SARS‐CoV‐2

Variants	Name/first detected	Lineage	Country of first detection	Mutations on S protein	Signature mutations	References
Variant of concern (VOC)	Alpha December 2020	B.1.1.7	UK	Δ69/70, Δ144/145, H69, V70, Y144, N501Y, A570D, D614G, P681H, T716I, S982A, and D1118H	N501Y	^27^, ^28^
	Beta December 2020	B.1.351	South Africa	D614G, L18F, D80A, D215G, Δ242‐244, R246I, K417N, E484K, N501Y, and A701V	Δ242‐244, R246I, K417N, E484K, and N501Y	^27^, ^28^, ^29^
	Gamma November 2020	P.1 (B.1.1.28.1)	Japan/Brazil	D614G, L18F, T20N, P26S, D138Y, R190S, K417T, E484K, N501Y, H655Y, and T1027I	K417T, E484K, and N501Y	^28^, ^30^, ^31^
	Delta May 2021	B.1.617.2	India	T19R, E156, F157, R158G, L452R, T478K, D614G, P681R, and D950N	L452R and T478K	^32^, ^33^, ^34^
	Omicron November 2021	B.1.1.529	South Africa	Δ69/70, A67V, H69, V70, T95I, S:G142, V143, Y144, Y145D, N211, L212I, G339D, S371L, S373P, S375F, K417N, N440K, G446S, S477N, T478K, E484A, Q493R, G496S, Q498R, N501Y, Y505H, T547K, D614G, H655Y, N679K, P681H, N764K, D796Y, N856K, Q954H, and N969K	H655Y, N679K, P681H, E484A, and N501Y	^35^, ^36^
Variant under monitoring (VUM)	Epsilon January 2021	B.1.427/B.1.429 (CAL.20C)	California	S13I, W152C, L452R, and D614G	L452R, S13I, and W152C	^37^, ^38^, ^39^
	Eta December 2020	B.1.525	South Africa	Q52R, A67V, ΔH69/V70, ΔY144/145, E484K, D614G, Q677H, and F888L	Δ69/70, Δ145, E484K, and Q677H	^40^
	Lota November 2020	B.1.526	USA	L5F, T95I, D253G, D614G, A701N, and S477N or E484K	L5F, T95I, D253G, S477N, or E484K	^41^
	Kappa October 2020	B.1.617.1	India	D614G, G142D, E154K, L452R, E484Q, P681R, Q1071H, and H1101D	L452R and E484Q	^36^, ^40^, ^42^
	C.1.2 May 2021	B.1.1.1.1.2	South Africa	E484K and E484Q L452R s	E484Q	^27^
Variant of interest (VOI)	Lambda August 2020	B.1.1.1.37 C.37	Peru	Δ247‐253, G75V, T76I, R246, S247, Y248, L249, T250, P251, G252, D253N, L452Q, F490S, D614G, and T859N	L452Q and T859N	^43^, ^44^, ^45^
	Theta February 2021	P.3 (B.1.1.28.3)	Philippines	D614G, ΔLGV141‐143, E484K, N501Y, P681H, E1092K, H1101Y, and V1176F	E484K, N501Y, and P681H	^46^, ^47^
	Mu January 2021	B.1.621	Kolumbien	T95I, Y144S, Y145N, R346K, E484K, N501Y, D614G, P681H, and D950N	Y144S and Y145N	^27^
	Zeta July 2021	P.2 (B.1.1.28.2)	Brazil	D138Y, R190S, E484K, H655Y, T1027I, and V1176F	E484K and V1176F	^46^, ^47^

### 2.2 Viropathology

During cell fusion, the virus enters the cell via superficial phospholipids. The incubation period of the virus lasts about 10 h, followed by an assembly process of about 10–12 h, in which finally the 10^∧^3/cell virus lyses the cell that leads to infection. The binding between SARS‐CoV‐2 and human cellular receptors is much stronger than the binding of their same family member viruses to these receptors. Insertion of 12 nucleotides among the sequence of S1 and S2 results in the formation of a protease cut site. This cut site is individual for the SARS‐CoV‐2 virus and is effected on higher pathogenicity of it.

### 2.3 Virus receptors

The angiotensin‐converting enzyme 2 (ACE2) cell receptor is an intramembrane protein with the hemostatic role in balancing the impacts of ACE on the cardiovascular system. In such virus infection, virus attaching to ACE2 leads to its endocytosis and decreases the amount of surface ACE2 on the endothelial cells, which can upset the balance between ACE and ACE2 and subsequently increase angiotensin II levels. Angiotensin II, commonly known as a vasoconstrictor, promotes inflammation by activating enzymes, such as a disintegrin and metalloprotease 17 (ADAM17).^48^, ^49^ In addition to ACE2, other receptors for SARS‐CoV‐2 entry were described, such as another member of the coronaviruses family‐like NL‐63. These receptors consist of CD209L (called L‐SIGN and CLEC4M) and CD209 (called DC‐SIGN), both of them are cell adhesion receptor members, such as the immunoglobulin family.^50^ The CD209L is overexpressing in type II alveolar cells and lung endothelial cells and the CD209 is expressing in dendritic cells (DCs) and tissue‐dwelling macrophages.^51^ Another receptor of this virus is Basigin, also called CD147 or EMMPRIN (extracellular matrix metalloproteinase inducer). This membrane glycoprotein (belonging to the immunoglobulin family) serves as a ligand for virus spikes. In the discussion of blocking receptors, the specificity of inhibitory drugs (antireceptors) has been based on the spatial epitopes of these surface glycoproteins.^52^, ^53^

### 2.4 Virus immunopathology

The resemblance of the SARS‐CoV‐2 virus with its family‐related viruses suggested that the immunopathogenic response due to host–pathogen interaction may be similar. Lack of adequate immune response in combat with SARS‐CoV‐2 can affect the disease severity. In less than 20% of patients leading to severe symptoms like acute respiratory distress syndrome, loss of lung organs, respiration, shock, and less than 2.4% of them lead to death.^54^ These conditions can be due to the immune system's malfunction in blocking and regulating the immune response. Overproducing of the proinflammatory cytokines (such as interleukin‐1 [IL‐1], IL‐2, IL‐6, IL‐8, IL‐17, and tumor necrosis factor‐alpha [TNF‐alpha]) and chemokines (CXCL10 and CCL2) by immune cells leads to cytokine storms. The second mechanism observed in these patients, similar to the MERS virus, is lymphopenia, especially T cells reduction, which can occur under different approaches. The lode of the virus, the age, health of the immune system, and the presence of chronic diseases due to imbalance in proinflammatory and anti‐inflammatory cytokines, are very important influential factors. Induction the expression of these cytokines and stimulation of their signal cascade is performed directly and indirectly by viral proteins (especially S and N proteins). Adequate knowledge about the disease's immunopathogenesis can be affected in selecting potential targets to increase the immune response.^55^, ^56^

### 2.5 Host immune response

#### 2.5.1 Innate immune response

This type of immune response has a vital responsibility in protecting or failing the responses against the virus. Single‐stranded RNA (ssRNA) or double‐stranded RNA of these kinds of viruses as pathogen‐associated molecular patterns engage in innate immune cells like neutrophils and monocytes––macrophages via the cytosolic RNA sensor (RIG‐I/MDA5) as intracellular or endosomal RNA receptors (TLR3 and TLR7) pattern recognitions. This engagement stimulates proinflammatory cytokines expression by launching downstream signaling cassettes, such as NF‐κB (nuclear factor kappa light chain enhancer of activated B cells) and IRF3 (IFN regulatory factor 3). Neutrophils, C‐reactive protein, and serum IL‐6 increasing and also total lymphocytes decreasing are other cases of infection with this virus.^57^ Increasing the proinflammatory cytokines can stimulate the neuroendocrine system, secreting glucocorticoids, and other peptides, subsequently impairing the immune response.^58^

#### 2.5.2 Adaptive immune response

Both humoral and cellular immunity will act against the virus. Humoral immunity works by producing neutralizing antibodies to limit reinfection by targeting viral antigens. The most potential antibodies after the isotype switching are IgG subtypes. Excessive secretion of these antibodies is present in acute conditions that can have adverse effects. One of these complications is an antibody‐dependent enhancement, happening in some types of viral infections. In this mechanism, the antibody itself becomes a way for the virus to attach to the cell and accelerate its entry and increase inflammation.^59^ Cellular immunity through cytotoxic T cells kills virus‐infected cells, and T helper type 1 (Th1) cells play a critical role in immunization against the virus. S, M, N, and E proteins are vital molecules in the development of immunogenicity, but also, S protein is involved in both humoral and cellular responses.^56^, ^60^

### 2.6 Pathogen immune evasion strategies

Most viral proteins, especially M, N, and S, can block the signaling pathway of type I IFN production and suppress the host's innate antiviral response. On the other hand, reducing antigen presenting by lowering the expression of major histocompatibility complex class I and class II (MHC‐I and II) is also another mechanism of the virus escaping from the adaptive immune response. Immune exhaustion, viral mutations, and immune deviation can be the other potential immune evasion methods.^57^, ^60^, ^61^

## 3 DETECTION

**FIGURE 2:**
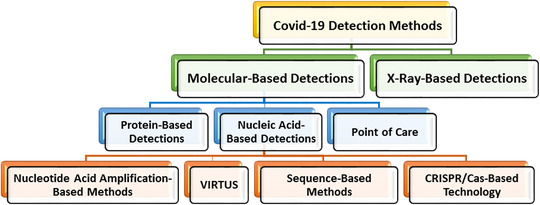
An overview of the COVID‐19 detection methods

Section 3 reviews the different COVID‐19 detection methods used so far. These methods can be seen as categorized in Figure 2.

### 3.1 Molecular detections of COVID‐19

COVID‐19 diagnostic instruments are usually available based on molecular detection in the market. Molecular detection, which is classified into two main groups based on nucleic acid and protein base methods, is the most powerful technology for detecting SARS‐CoV‐2 so far.^62^, ^63^, ^64^ Some of which are laboratory tests, such as next‐generation sequencing (NGS), PCR, enzyme‐linked immunosorbent assay (ELISA), chemiluminescent immunoassay (CLIA),^65^ and some others are POC tools^66^ like GICA.^67^ Besides nucleic acid and protein detection, numerous techniques are established to detect the whole virus in specimens, such as flow‐virometry.^68^

#### 3.1.1 Nucleic acid‐based detections

##### 3.1.1.1 Nucleotide acid amplification‐based methods

Detection of the SARS‐CoV‐2 viral genome, consisting of ssRNA, is effectively done by quantitative reverse transcription PCR (RT‐qPCR), which is commonly used as the molecular diagnostic gold standard.^69^, ^70^ The tests contain nucleic acid extraction/purification from samples and finally RT‐PCR.^69^ The approved detection kits are based on different viral targets, including RdRp, envelope (E), spike (S), open reading frame (ORF1ab) 1a, and nucleocapsid protein (N).^70^, ^71^ The LoD gets to levels less than 10 genome copies per reaction (0.5 cp/μl), depending on the chosen kits and PCR instrument. Although RT‐qPCR is sensitive and reliable, it takes at least 2 h. It needs a specific tool, trained technicians, and well‐equipped laboratories, limiting its application in underequipped laboratories or on‐site.^72^, ^73^ In addition, in order to increase the sensitivity and accuracy, several practical considerations like sample quality, viral load, sampling methods, and sample source must be taken into account when performing diagnostic assays by RT‐qPCR.^74^ A method called RTLAMP (reverse transcription loop‐mediated isothermal amplification (IA)) has been developed for quick and easy detection of SARS‐CoV‐2 nucleic acid.^75^ The LAMP reaction, which is a reliable and rapid assay with the low equipment cost, is performed at constant temperature (usually 65°C) for 20 min. The testing outcome is detected by a color change, which can be analyzed directly by the naked eye. Due to the fact that this method uses four sets of primers that target ORF1ab, S, and N genes of SARS‐CoV‐2, its specificity is high. The detection limit of the technique was 80 copies of viral RNA per milliliter sample.^76^ Another technique based on IA is recombinase polymerase amplification (RPA), which does not require a PCR machine. Amplification is performed on a specific combination of enzymes and proteins followed by fluorescence measurements by tube scanners to detect. The whole process is performed at a constant temperature (between 39 and 42°C) under 20 min and the clinical sensitivity and specificity of this about SARS‐CoV‐2 are 98% and 100%, respectively.^77^ These robust, accurate, and simple operation methods have solved RT‐qPCR problems, such as time‐consuming and expensive equipment. Nonetheless, there is a need for the one‐step process to accomplish RNA amplification directly from a throat swab sample without RNA extraction, which can be used as a rapid on‐site test.^78^

##### 3.1.1.2 CRISPR/Cas‐based technology

The clustered regularly interspaced short palindromic repeats (CRISPR)‐based nucleic acid recognition systems incorporate RPA with CRISPR–Cas12/13 enzymology for particular target detection.^79^, ^80^ In one study, SHERLOCK (high‐sensitivity enzymatic reporter unlocking) technology was developed, which targets S and ORF1ab genes. In this method, first, amplification is performed using RPA, and then targeting of preamplified viral RNA sequence using Cas13, leading to generate fluorescence and detection of COVID‐19. To enable SHERLOCK for rapid detection assay, they introduced STOPCovid (SHERLOCK Testing in One Pot), performed at a single temperature without the need for sample extraction and a simple visual using lateral flow readout. LOD of this method is 100 copies of the viral genome per reaction.^81^ Currently, two of the CRISPR‐based SARS‐COV‐2 diagnostic tests have received FDA emergency use authorization that are now available for field use, and will become more common.^82^ Today, despite new and valuable approaches to SARS‐CoV‐2 detection, the vast majority of tests worldwide are still performed using PCR‐based methods. To date, among available molecular‐based assays for the detection of SARS‐CoV‐2 in the United States, 90% are PCR‐based methods, 6% are IA assays, 2% are hybridization techniques, and 2% are CRISPR technology.^83^

##### 3.1.1.3 Sequence‐based methods

Whole‐genome sequencing is another technology to identify the Deoxyribonucleic acids (DNAs), which can be utilized for detection and evaluation of mutational properties of the SARS‐CoV‐2.^84^ Because of being costly, time‐consuming, and intricate, this method is unsuitable for crucial and large‐scale testing. So, new strategies based on sequencing were developed. A laboratory in Wuhan developed a novel approach, nanopore target sequencing. At the primary step, the amplification of different SARS‐CoV‐2 gene fragments is performed. The nanopore platform is involved in the sequencing of the amplicons. Nanopore system can sequence and evaluate the outputs at the same time, letting us to detect SARS‐CoV‐2 in short time. Also, by adding multiple characteristic primers, it can be used for simultaneously analyzing and identification of various types of viruses, including influenza.^78^ In one of the published researches, INSIGHT (Isothermal NASBA sequencing‐based high‐throughput test) was proposed. This is a two‐phase approach and is performed by an isothermal nucleic acid sequence–based amplification (NASBA) reaction followed by NGS. The sensitivity of this method is high (10–100 copies per reaction), and confirming the rapid results of the first stage by NGS analysis improves the test accuracy in a high‐throughput way.^85^ Another strategy is massively parallel diagnostic assays, which combines reverse transcriptase (RT), PCR, and NGS for detection. RT primers with highly unique barcode sequences link to three amplicons (N1 and N2 SARS‐CoV‐2, RNase P human control). After the RT reaction, the cDNA of patient samples is pooled together and amplified in a single PCR, followed by NGS. Using patient‐specific primers at the RT step enables this method simultaneously to carry out thousands of viral RNA measurements in a single assay.^86^ These methods are transportable, scalable, accurate, rapid, and cost‐effective strategies for detecting SARS‐CoV‐2; however, further development is needed to bring them into practical use.

##### 3.1.1.4 VIRTUS

A sensor named VIRTUS (Viral Transcript Usage Sensor) was developed for detecting and quantifying mRNA transcripts in the data of traditional human RNA‐seq data. VIRTUS is applied to identify the cells sheltering activated viruses and not the copy number of the virus, the composition of multiple viruses in a cell, and the expression differences between infected and uninfected cells.^87^

#### 3.1.2 Protein‐based detections

Immunological assays have also been used as COVID‐19 detection. They have been categorized in antigen base and antibody‐based (serological) detection. Antigen testing has been used as diagnostic tools performed by ELISA and lateral flow assay (LFA) using nasopharyngeal and oropharyngeal samples and mAbs to target SARS‐CoV‐2 antigens. However, there is concern about their sensitivity.^88^, ^89^ One study found that a rapid SARS‐CoV‐2 antigen COVID‐19 diagnostic test based on LFA technology had a sensitivity of 82.2% for CT values under 25.^80^ Unlike antigen testing that can be used as detection tools for viral infection, serological immunoassays have different roles and serve as screening and surveillance tools during the prevalence of COVID‐19.^90^ SARS‐CoV‐2 specific antibodies are usually detected more than 1 week after symptom onset.^91^ To recognize these antibodies in serum samples, recombinant viral nucleocapsid or spike (S) proteins, part of them like S1, S2, and RBD, or a mixture, have been immobilized to detect different classes of anti‐SARS‐CoV‐2 antibodies, which include immunoglobulin A (IgA), IgM, IgG, or IgM/IgG in total.^92^ Utilization of whole viral antigens for serological methods, due to containing conserved domains, raises the possibility of cross‐reactivity with other common circulating CoVs.^93^ Protein‐based detection of COVID‐19 is not limited to manual ELISA. Digital ELISA, CLIA, surface plasmon resonance (SPR),^94^ flow cytometry,^95^ lateral flow immunoassay (LFIA),^96^ and microfiber‐based immunoassay^97^ are other protein‐based tests, which have been used or are in progress for the diagnosis of SARS‐CoV‐2. Digital ELISA, also called a single‐molecule array (Simoa), is a technique whose sensitivity is in the femtomolar range. In this method, the array amounts are almost two billion times lower than a traditional ELISA.^98^ In one study, developing a Simoa immunoassay on the automated system to identify SARS‐CoV‐2 N‐protein in venous and capillary blood was reported.^99^ In another study, an ultrasensitive digital ELISA based on a Simoa was developed, which is rapid and can simultaneously detect spike and nucleocapsid proteins of SARS‐CoV‐2.^100^ CLIA is a solid‐phase immunoassay method in which the label is a luminescent molecule. Other advantages of this method include high signal intensity, absence of interfering emissions, and reduced incubation time.^101^ In a study about diagnosing SARS‐CoV‐2 infection, it was shown that specificity for IgG was greater than 98% for CLIA.^102^ SPR is a label‐free technique and does not require additional reagents, assays, or laborious sample preparation steps and is mostly used for large biomolecules. This optical method has been incorporated into immunoassay tools, especially in detecting antibodies.^103^, ^104^ In the same context, a sensor detecting nucleocapsid antibodies specific against the COVID‐19 was reported, which can use a human serum as a sample.^94^ The flow cytometric approach was also validated based on antigen‐expressing human embryonic kidney 293 (HEK 293) cells to evaluate spike‐specific IgG and IgM antibody reactions. Regarding the results, the specificity and sensitivity were comparable to ELISA or CLIA.^53^

#### 3.1.3 Point of care

Laboratory tests require expensive materials and equipment and take time. In contrast, POC devices are simple, inexpensive, and in‐house tests that usually take less than 1 h to respond.^105^, ^106^ As of October 12, 2021, the Foundation for Innovative New Diagnostics (FIND), which publish the lists of all commercially available immunoassays molecular tests for COVID‐19, reports that about 402 flow assay strips or cassettes are commercially available.^107^ Microfiber‐based immunoassay, which is microfiber‐based arrays of antigens to capture specific antibodies,^99^ and LFA are some of the POC devices for molecular detection. LFIA is a kind of cellulose‐based device and usually employs gold nanoparticles to label antigen or antibody to identify SARS‐CoV‐2‐specific antibody or antigen in specimens.^108^ LFA has also been used for nucleic acid recognition. For instance, the LAMP POC device is used for the fast and on‐site recognition of COVID‐19, a combination of lateral flow strip and LAMP assay.^106^ The other end of care method is RPA with lateral flow dipstick reaction, which is a nucleic acid base method and can be performed in less than 20 min at 10–37°C.^109^

In conclusion, diagnostics are essential for dealing with outbreaks to diagnosis, surveillance, vaccine design, and so on.^110^ The congenital COVID‐19 diagnostic methods are different in sensitivity, accuracy, specificity, and application. For instance, RT‐PCR is a method to detect viral nucleic acid, while serological tests are beneficial for surveillance^111^ and vaccine studies.^94^ Factors that play a vital role in preventing false negatives in COVID‐19 as well as other respiratory diseases include sampling method, sampling time based on symptom onset, and method of sample transmission to the laboratory.^112^ The other important parameter is selecting a suitable target based on virology and biology of SARS‐CoV‐2.^113^ Up‐to‐date, a variety of tests have been commercialized with or without FDA‐EUA approval, and others are in research and development.^64^

### 3.2 X‐ray‐based detections

Nowadays, RT‐PCR is used as an accepted standard method for the identification of COVID‐19 infection. However, this laboratory test is expensive and time‐consuming. Moreover, the false‐negative test result is possible because of insufficient quantities of viral load and incorrect extraction method.^114^ Considering all the above disadvantages, we need an accurate, fast, and cost‐effective way to detect COVID‐19. Chest CT can be an indispensable tool for screening and diagnosing people suspected of COVID‐19. In this regard, two common abnormalities in the lung, such as grand‐glass opacities and consolidating, are diagnosable by chest CT. The importance of CT is not only limited to detection and diagnosis, but also this test can be used for treatment evaluation and follow‐up.

Nevertheless, some studies reported that patients with a severe form of COVID‐19 have positive RT‐PCR results with standard CT. One possible explanation for this phenomenon is that SARS‐CoV‐2 targets multiple organs, such as the heart, kidney, and liver, so comprehensive examinations should be performed for better understanding.^114^, ^115^ Based on a meta‐analysis, the sensitivity of chest CT was great in Wuhan (96–99%) and in other regions of the world was varied from 61% to 98%.^116^

## 4 PRECLINICAL STUDIES

Cell lines and organoids are a rapid system for studying the interactions and infection processes of the virus with limitations in understanding pathology, antigenic drift, and virus evolution in this system. So, animal models provide the advantage of studying virus replication according to the physiological symptoms of an organism.^117^ Despite FDA regulations' flexibility in saving time, in research on SARS‐CoV‐2 vaccines and antiviral drugs, animal models will play a critical role in preclinical studies. Animal models of nonhuman primates, including African green monkeys, rhesus monkeys, and cynomolgus monkeys, can infect via SARS‐CoV‐2 with severe lesions and histopathological changes in vital organs. The rhesus monkey is more sensitive, and the African green monkey has more respiratory‐related symptoms.^118^, ^119^ The similarity of the ACE2 receptor in rhesus macaques, Syrian hamsters, and common marmosets is very high and reaches 100% in rhesus macaques.^118^ The hamster ACE2 has the highest affinity for SARS‐CoV‐2 spike proteins. The viral load in this animal increases, leading to diffuse alveolar damage in the early stages and apoptosis in the later stages of infection. Ferret is more susceptible to the virus than cats, with differences in only two amino acids in ACE2.^120^ Because the virus affects the ferret's upper respiratory tract, they are a potential animal model for evaluating vaccines efficacy.^121^, ^122^ Owning to mice's advantages, such as small size and availability, these animals are suitable models for preclinical researches. But mouse ACE2 receptor has less similarity to human ACE2 and low binding affinity for entering the SARS‐CoV‐2 viruses via their spike protein. Therefore, for studying the transmission and pathogenesis of the virus, evaluating antiviral drugs and vaccine developments, it is necessary to produce mouse‐adapted SARS‐CoV‐2 strains to develop mild to severe disease suitable for studies.^123^ There are two pathways to the manipulation of mice for SARS‐CoV‐2 studies listed in Tables 2 and 3. The first strategy is producing human angiotensin converting enzyme 2 (hACE2) mice to develop vaccines and other potential antiviral therapies, and the second knockout of other genes to mimic disease.

**TABLE 2:** Manipulation of mice for SARS‐CoV‐2 studies: producing hACE2 mice for the development of vaccines and other potential antiviral therapies

	Mouse model	Identification	Utilities	Benefits	Limitations	References
hACE2 mice	Adenovirus serotype 5 (Ad5)‐hACE2‐transduced mice	Lung expression of human ACE2 by transduction of adenovirus 5 in mice	Useful for the assessment of SARS‐CoV‐2‐specific therapies, such as vaccine evaluation, human convalescent plasma therapy, and antiviral therapies	Rapid and efficient mouse lungs expression, and manufacture of an easily reproducible murine model for SARS‐CoV‐2 within 2–3 weeks	Severe and extrapulmonary manifestations of the disease	^124^
	hACE2 knockin mice	ACE2 humanized mouse by CRISPR/Cas9 knockin method	Evaluation of the potential therapeutics Development of vaccines; elucidation of the transmission and pathogenesis Validation of the risk factors dependent on the intense symptoms in COVID‐19	In the mice susceptible to SARS‐CoV‐2 contamination upon intranasal inoculation, hACE2 antigen expression was discovered in the kidneys, vascular endothelium, skeletal muscle, lungs, adrenal, liver, pancreas gastrointestinal, heart, spleen, LN, smooth muscle, and ganglia, and as a consequence, pulmonary contamination and pathological variations be similar to COVID‐19 patients	Requires longer periods of time to cross or genetically modify these mice	^125^
	hACE2‐transgenic mice	pCAGGS‐ACE2 plasmid with CAG promoter (AC70, AC22, and AC63 mouse lineage)				^126^, ^127^
		pK18‐hACE plasmid with human K18 promoter				^128^
		pEGFP‐N1ACE2 plasmid with mouse ACE2 promoter				^129^
		HFH4‐ACE2 plasmid with human HFH4 promoter				^130^

**TABLE 3:** Manipulation of mice for SARS‐CoV‐2 studies: knockout of other genes to mimic disease

	Mouse model	Utilities	Benefits	References
Knockout mice	ACE2 knockout mice	Model for coronavirus‐induced cytokine storm‐driven inflammation, etiology, and treatment	Stimulation of acute respiratory distress syndrome (ARDS)	^131^, ^132^
	Tmprss2 knockout mice	COVID‐19 disease pathogenesis	Development of pneumonitis to accompany with viral replication	^133^, ^134^
	Stat1 knockout mice	Model for morbidity, viral replication, and mortality	Antivirals and pathogenesis studies	^135^, ^136^

## 5 CLINICAL INFORMATION

Various sampling paths have been introduced for RT‐PCR, such as pharyngeal swab specimens, oropharyngeal swab specimens, urine, and stool samples.^137^, ^138^ Fecal and urine sampling is easier than nasopharyngeal swabs and sputum specimens, while the quantity/quality of the samples are simply determined and increase the diagnosis of asymptomatic patients.^139^ The ordinary COVID‐19 disease symptoms are fever (>37.8°C), cough, asthenia, and dyspnea,^140^, ^141^ but patients with positive stool tests did not suffer gastrointestinal issues and had nothing to do with the severity of the lung infection^138^ and some people have no symptoms.^141^ Altered mental status, myocardial, hepatic, and kidney injuries have been reported not only among older patients but also in younger patients.^142^ The nonpersistent cough, hoarse virus voice, nausea and vomiting, shortness of breath, nasal discharge or congestion, headache, wheeze, muscle aches, diarrhea, and loss of sense of taste/ smell have also been reported in some cases.^141^ The main signs of the onset of the disease in children include abdominal pain, vomiting, and headache.^143^, ^144^ A study showed that in some patients with CT scan confirmed COVID‐19, the PCR test was negative.^145^ In contrast, some COVID‐19 positive patients do not represent any of the symptoms mentioned above, and the development of new symptoms makes the diagnosis more complicated.^146^

### 5.1 Abdominal symptoms

A 55‐year‐old man was hospitalized with abdominal ache in the left iliac fossa without the usual signs of COVID‐19, and no significant changes in the respiratory system were CT‐scanned. In evaluating the thoracoabdominal transition pictures, separate ground‐glass opacities were discovered on the periphery of the middle lobe and posterior basal segment of the right lobe. A full chest CT was taken for better assessment of degree of pulmonary involvement in which a viral infectious process was found similar to the documents representing pulmonary involvement in COVID‐19. This finding was reconfirmed by real‐time PCR through the nasal section and oropharynx swab. In another case, an 84‐year‐old man was referred to the hospital with abdominal pain, 6 days of fever 38°C, mild diarrhea without vomiting, nausea, or abdominal symptoms. A chest CT revealed multiple ground‐glass and crazy‐paving pulmonary opacities in multifocal, predominantly peripheral, bilateral, and posterior distribution, mostly in the lower lobes, which confirms the COVID‐19 infection with certainty.^147^ In this case, COVID‐19 PCR on oropharynx swab was positive, and laboratory tests demonstrated increased inflammatory serum biomarkers with normal blood cell counts. Patients who were followed‐up for autoimmune liver disease, cirrhosis, chemotherapy for hepatoblastoma, and transplantation, none developed a pulmonary disease appeared to be the major driver of the lung tissue injury through this infection.^148^, ^149^

### 5.2 Hypertension

Although 30% of hospitalized COVID‐19 patients have shown hypertension, it should be considered that the hypertension occurrence among the age‐matched healthy population is within the same range. Therefore, the hypothesis in which hypertension (and its therapy with ACE2 inhibitors/ARB) rises the danger of severe COVID‐19 infection is not supported by these findings. It is best not to increase the dose of angiotensin receptor blocker (ARB) drugs in SARS‐CoV‐2‐infected patients and not to start a new ARB therapy.^150^, ^151^

### 5.3 Diabetes

Published data suggest that the incidence of diabetes mellitus (DM) in patients with COVID‐19 is higher than normal.^152^, ^153^, ^154^, ^155^, ^156^, ^157^, ^158^, ^159^, ^160^ Diabetes can be assumed as an independent risk factor for increased ICU admissions, need for ventilation, and eventually death.^161^, ^162^ It can be suggested that diabetic patients are at a significantly enhanced risk of SARS‐CoV‐2 and COVID‐19. Possibly, enhanced ACE2 expression in DM patients contributes to enhanced sensitivity toward these infections.^163^ Also, relatively high ACE2 expression in the pancreas islets may contribute in hyperglycemia among COVID‐19 patients.^164^ An asymptomatic girl was admitted to the hospital without clinical symptoms. The throat swab was COVID‐19 negative by RT‐PCR, while the urine test was positive. After antiviral and symptomatic supportive treatments, the throat swab became positive. During the second and third weeks, both tests became negative, while 1 month later, the patient felt well, and throat swab RT‐PCR was negative.^139^

### 5.4 Renal dysfunction

Podocytes and proximal convoluted tubules, as potential host cells for SARS‐CoV‐2, show considerable expression of coexpression of ACE2 and TMPRSS genes. Severe renal malfunction is one of the fatal complications in COVID‐19 patients. Pathophysiological research has shown that this complication resulted by the virus can cause cytopathic effects.^165^, ^166^ Reports indicate that 0.5–19% of COVID‐19 patients develop acute kidney injury. In studies related to COVID‐19, contrast‐enhanced imaging (CT and MRI) should be used with more caution, as impaired renal function enhances patients' sensitivity to contrast‐induced nephropathy.^167^, ^168^

### 5.5 Cardiac manifestation

ACE1 is a transmembrane aminopeptidase that is a target receptor for SARS‐CoV‐2. It is implicated in the development of hypertension and significantly expressed in the heart. Accordingly, the probability of cardiovascular damage/myocarditis is also believed as a symptom of COVID‐19. Furthermore, one clinical study reported five confirmed cases of SARS‐CoV‐2 that showed advanced myocardial damage through infection. Myocardial injuries are mostly represented by enhanced levels of biochemical markers, containing cardiac creatine kinase, troponin I, lactate dehydrogenase, and α‐hydroxybutyrate dehydrogenase.^168^, ^169^, ^170^

### 5.6 Mediastinal findings

Mediastinal lymphadenopathy has been observed in COVID‐19 patients.^171^ A recently published article reported enlarged mediastinal lymph node as a common symptom in patients with severe COVID‐19. Therefore, lymphadenopathy is another symptom of COVID‐19, especially in acute patients.^172^, ^173^

### 5.7 Neurological discoveries

Corona family viruses can go into the central nervous system (CNS) via the neuronal retrograde route or bloodstream heading to encephalitis or meningitis with mortality and morbidity. While viral encephalitis can continue undiagnosed because of symptoms absence, acute viral encephalitis can affect body temperament, mental status, abnormal motor movement, irregular behavior/speech, and focal neurological irregularities as flaccid paralysis, hemiparesis, paresthesia, or seizures.^174^ SARS‐CoV‐2 can find its path via the circulation or through the cribriform plate of the ethmoid bone and attack the CNS. It can also interact with ACE2 receptors and damage nerve tissues. COVID‐19 cerebral attachment via the cribriform plate may cause further complications, such as hyposmia/anosmia.^175^, ^176^, ^177^

### 5.8 Hematological symptoms

Pathological studies on SARS‐CoV‐2 pathogenesis derived that the COVID‐19 disease was instead a hypersensitivity pneumonitis than viral pneumonia.^178^ SARS‐CoV‐2 stimulates a cytokine storm (hyperactive immune response) and spilled high levels of cytokines into the circulatory system, which leads to systemic issues across multiple organs. SARS‐CoV‐2 pneumonia can cause multiorgan failure by overproduction of proinflammatory cytokines combine with a diminished oxygenation capacity of the patient's blood. Other symptoms of severe cases contain septic shock, difficult‐to‐correct metabolic acidosis, and coagulation dysfunction.^179^, ^180^, ^181^, ^182^, ^183^

### 5.9 COVID‐19 and pregnancy

Recent studies on pregnant women infected with COVID‐19 have shown that the main symptoms of their clinical manifestations include fever and cough and are no different from those of nonpregnant adults. Also shown that pregnant women are more prone to COVID‐19 and its complications and may even become severely ill.^184^ Even though there is no significant evidence to confirm vertical transmission, the possibility of mother‐to‐child transmission of COVID‐19 or SARS infection is not ruled out.^185^, ^186^, ^187^, ^188^, ^189^

### 5.10 Autoimmune diseases

Through an examination of patients who suffer from autoimmune diseases, such as rheumatoid arthritis, systemic lupus erythematosus, and Sjogren's syndrome, it was shown that anti‐SARS‐CoV‐2 IgG and IgM antibodies were not detected in serum samples, indicating that there was no cross reactivity between autoantibodies and SARS‐CoV‐2 antibodies.^190^, ^191^

### 5.11 Cancer

The results of a study conducted on cancer patients revealed that these patients might be more susceptible to infection in comparison to noncancer subjects. The rate of COVID‐19 mortality was higher in cancer patients showing worse prognoses for older ages and women. Combination therapy by antiviral medicines plus hydroxychloroquine (HCQ) seems superior to HCQ alone.^192^ In another study, the infection caused severe clinical events in Chinese cancer patients.^193^ It was also reported that 10.7% of virus‐positive cancer patients had symptoms.^194^, ^195^

## 6 TREATMENT

There is still no cure for COVID‐19. However, only one treatment, a drug called remdesivir, has been approved by the FDA for this disease, and research suggests that it provides only modest benefit to patients.^196^ Therefore, the best first‐line actions against this disease are staying home, washing hands, wearing the face mask, and having adequate rest. In this review, we summarized some potential treatments against this new emerging virus.

### 6.1 Therapeutic agents and inhibitors against COVID‐19

Here, we aim to present some of the potential and repurposed drugs for the treatment of COVID‐19.

#### 6.1.1 Antiviral drugs

**FIGURE 3:**
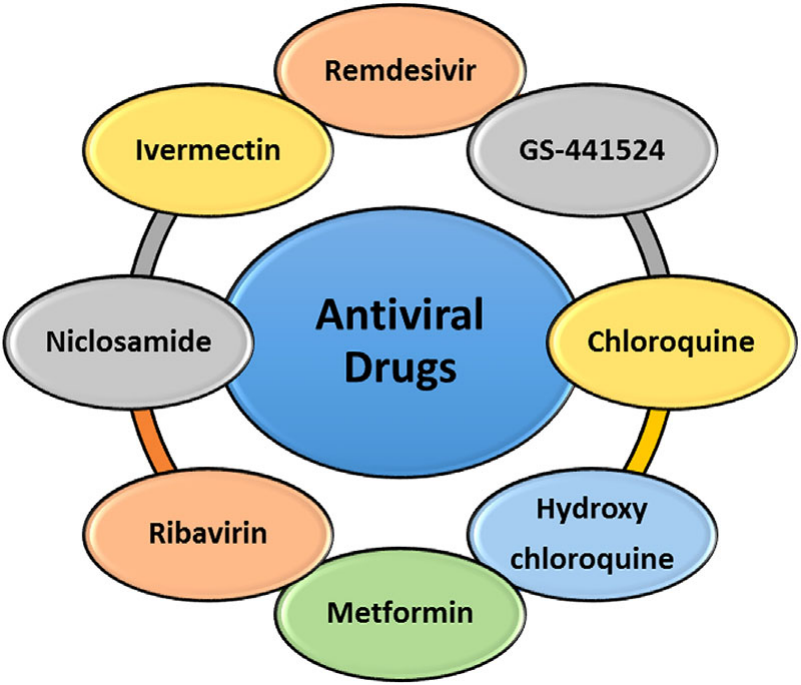
Antiviral drugs against COVID‐19

The most important antiviral drugs used to treat COVID‐19 virus are listed in Figure 3 and are discussed in detail below.

##### 6.1.1.1 Remdesivir

Remdesivir (GS‐5734), a broad‐spectrum antiviral agent, was synthesized and developed in 2017. It is a monophosphate prodrug, which belongs to the class of nucleotide analogs (Table S1). Owing to its low half maximal effective concentration (EC_50_) and host polymerase selectivity against the Ebola virus, it was used to treat the Ebola virus disease. This drug is adenosine analog and decreases viral RNA production by obscuring viral RNA polymerase and evading proofreading by viral exonuclease.^197^ Moreover, further research revealed considerable antiviral activity against MERS‐CoV and SARS‐CoV viruses, particularly human coronavirus 229E.^198^, ^199^, ^200^, ^201^, ^202^ As an only drug approved by the FDA for the treatment of SARS‐CoV‐2, it is currently being monitored in multisite clinical trials. The evidence of the antiviral effects of GS‐5734 on coronaviruses in vitro and in vivo studies have demonstrated favorable efficacy and low toxic side effects.^198^, ^199^, ^203^, ^204^ One survey showed that the combination of remdesivir and emetine has a synergy effect and inhibits SARS‐CoV‐2 replication in vitro.^205^ Moreover, the efficacy of remdesivir and chloroquine (CQ) in the inhibition of COVID‐19 was demonstrated.^206^ Also, the U.S. FDA has issued an emergency license for remdesivir to expedite the treatment of SARS‐CoV‐2.^196^

##### 6.1.1.2 GS‐441524

GS‐441524 is an antiviral drug that is the parent nucleoside of remdesivir (Table S1). It has been found that GS‐441524 exhibits antiviral activity on SARS coronavirus (SARS‐CoV), Marburg virus, and feline infectious peritonitis virus. Several in vitro and in vivo studies on this drug against COVID‐19 using a mouse and nonhuman primate animal models have reported favorable outcomes.^197^, ^203^ Nevertheless, another investigation based on a more comprehensive pharmacokinetic rationale supported the application of GS‐441524 over remdesivir for the treatment of SARS‐CoV‐2 due to its synthetic simplicity and in vivo efficacy in the veterinary setting.^202^

##### 6.1.1.3 Chloroquine

CQ is an antiviral drug that has been long employed in the prevention and treatment of malaria and also exhibited in vitro activity against the replication of some coronaviruses, such as the SARS‐CoV‐2 (Table S1).^197^, ^207^, ^208^ The safety and tolerability of this drug have been approved previously.^209^ It was found that CQ inhibits the glycosylation of host receptors, proteolytic processing, and endosomal acidification, which results in blocking viruses from entering into cells. Furthermore, it modulates immunity by attenuating cytokine production and the inhibition of autophagy and lysosomal activity in host cells.^210^, ^211^ Consequently, this molecule was suggested as a potential drug in the treatment of COVID‐19.^212^ However, this drug's efficacy (with or without a macrolide) in patients with COVID‐19 was not confirmed by available clinical data, although an increased frequency of ventricular arrhythmias and decreased in‐hospital survival were observed.^213^, ^214^ According to the latest update of the Coronavirus Drug and Treatment Tracker (October 7, 2021), this drug is not considered promising, meaning early evidence suggests that these treatments do not work.^215^

##### 6.1.1.4 Hydroxychloroquine

HCQ is an antiviral and antimalarial drug, a safer, less toxic, and more potent derivative of CQ. It is currently widely used to treat autoimmune diseases, rheumatoid arthritis, systemic lupus erythematosus, sarcoidosis, alopecia areata, and antiphospholipid syndrome (Table S1).^207^, ^211^ This drug directly inhibits viral entry into cells with the same mechanisms of action for CQ. So, it has been proposed as an antiviral drug in the treatment of several coronaviruses, especially SARS‐CoV‐2.^198^, ^199^, ^211^, ^216^ In preliminary clinical trials, the clinical efficacy of HCQ in combination with azithromycin in vivo was reported.^217^, ^218^ On the other hand, the use of HCQ (with or without a macrolide) in patients hospitalized with COVID‐19 was not supported by other studies, and adverse side effects were also found in the patients.^214^, ^215^, ^219^, ^220^, ^221^ An open‐label nonrandomized clinical trial demonstrated that HCQ reduced viral load in COVID‐19 patients, and azithromycin reinforced its effect.^222^ Finally, the FDA warns that the drug can have several serious side effects on the heart and other organs when used to treat COVID‐19. On March 2, a WHO expert panel strongly advised against the use of HCQ in all patients, adding that the drug was no longer a research priority.^215^

##### 6.1.1.5 Metformin

Metformin was initially announced as an anti‐influenza medication that glucose‐lowering was known as one of its side effects. This drug has pleiotropic effects and may be effective against hepatitis C virus (HCV), hepatitis B virus, and human immunodeficiency virus (HIV). At the molecular level, metformin phosphorylates AMP‐activated protein kinase (AMPK) in hepatocytes and causes its activation. The SARS‐CoV‐2 binds to the ACE2 receptor to enter the host cell using RBD in the spike protein. It has been hypothesized that ACE2 causes acute lung injury through the AMPK pathway and autophagy. Therefore, the phosphorylation of ACE2 can change its conformation and function and reduce virus entry.^223^ In this regard, a retrospective analysis demonstrated that metformin treatment decreased the mortality rate in COVID‐19 patients with diabetes.^224^

##### 6.1.1.6 Ribavirin

Ribavirin, a guanosine analog, has antiviral activity, so it is recommended for COVID‐19 treatment. The efficacy of the combination of ribavirin with IFN alfa in virus clearance and survival of patients was reported in the experience of MERS‐CoV. However, no significant benefit was observed with ribavirin treatment compared to the control group in hospitalized patients with severe or critical COVID‐19.^198^, ^199^, ^225^

##### 6.1.1.7 Niclosamide

Niclosamide, an FDA‐approved anthelminthic drug, has various antiviral activities. The efficacy of this drug against SARS‐CoV, MERS‐CoV, ZIKV, and HCV has been investigated. The potential antiviral mechanism of niclosamide against SARS‐CoV‐2 is inhibition of autophagy, viral application, and receptor‐mediated endocytosis.^226^, ^227^ The clinical efficacy of niclosamide against COVID‐19 should be evaluated.

##### 6.1.1.8 Ivermectin

Ivermectin is a wide‐ranging antiparasitic that was approved by the FDA (Table S1). It has been reported to exhibit in vitro efficacy against several viruses, such as dengue virus, Zika virus, yellow fever virus, SARS‐CoV‐2, and so on. However, this drug has some side effects, such as neurotoxicity.^228^ The single addition of this inhibitor of COVID‐19 to Vero‐hSLAM cells led to over 5000‐fold reduction in viral RNA within 48 h. Nonetheless, an increase in time, that is, up to 72 h, did not result in more viral replication reduction. Finally, a trial on 1500 patients found no benefit from ivermectin.^215^, ^229^

#### 6.1.2 Calcineurin inhibitors

##### 6.1.2.1 Cyclosporine (cyclosporin)

Cyclosporine (cyclosporin) is a calcineurin inhibitor and belongs to immunosuppressant drugs used to prevent rejection after organ transplantation (Table S1). Since it binds to cyclophilins in cells and consequently inhibits replicating different coronaviruses, it has been proposed to use against COVID‐19.^230^, ^231^ Nevertheless, clinical trials should be considered in further studies.

#### 6.1.3 Cyclophilin inhibitors

##### 6.1.3.1 Alisporivir

Alisporivir is a nonimmunosuppressive cyclophilin inhibitor, which is derived from cyclosporine (Table S1). This inhibitor blocks the replication of four different coronaviruses, such as SARS and MERS.^232^ It also inhibits in vitro SARS‐CoV‐2; thus, it has been recommended as a potential candidate for the treatment of COVID‐19.^233^

#### 6.1.4 HCV inhibitors

##### 6.1.4.1 Sofosbuvir

Sofosbuvir is a clinically approved drug against the HCV (Table S1). The high sequence and structural homology in the RdRp of the HCV and SARS‐CoV‐2 viruses support the idea that sofosbuvir can effectively inhibit the SARS‐CoV‐2 RdRp. Moreover, the safety profile of this drug has been known due to its long‐lasting application in patients for treating and eradicating HCV chronic infection. As a result, it has been suggested to use sofosbuvir to treat SARS‐CoV‐2.^234^, ^235^

#### 6.1.5 HIV protease inhibitors

##### 6.1.5.1 Lopinavir/ritonavir (LPV/r)

Lopinavir (LPV) is an antiretroviral that inhibits the activity of the protease. Also, ritonavir (RTV) is another antiretroviral that belongs to the protease inhibitor class (Table S1). RTV helps to stabilize LPV through an increase in its plasma half‐life. The combination of these agents results in lopinavir/ritonavir (LPV/r) approved by the U.S. FDA and is used to treat HIV. It has also been shown to have in vitro and in vivo activities on other new coronaviruses, such as SARS and MERS, by inhibiting the 3‐chymotrypsin‐like protease (3CL^pro^), also known as main protease (M^pro^).^198^, ^200^, ^236^, ^237^ However, the use of LPV/r for the treatment of COVID‐19 was not supported.^198^, ^199^, ^219^, ^238^ In the case of MERS‐CoV, the efficacy of a combination of LPV‐RTV with ribavirin and IFN alfa has been studied. However, a trial study of LPV/RTV in adults hospitalized with severe COVID‐19 showed that there is no benefit with LPV‐RTV treatment beyond standard care.^239^

##### 6.1.5.2 Nelfinavir

Nelfinavir is another HIV‐1 protease inhibitor that reduced the replication of SARS‐CoV‐2 in vitro. So, it can be considered as a potential drug in the treatment of COVID‐19.^240^ Moreover, this drug may inhibit SARS‐CoV‐2 spike‐mediated cell fusion.^241^ One study demonstrated the high potency of nelfinavir against SARS‐CoV‐2 in Vero E6 cells. However, further exploration as a potential treatment for COVID‐19 is needed.^242^

##### 6.1.5.3 Darunavir/cobicistat

This combination can be a potential alternative to LPV/RTV based on a similar mechanism of action. However, a single‐center, randomized, and open‐label trial on mild patients with confirmed COVID‐19 demonstrated that this HIV‐1 protease inhibitor might not have clinically significant anti‐SARS‐CoV‐2 activity. As a result, more studies are needed to investigate its role.^225^

#### 6.1.6 Anti‐influenza drugs

##### 6.1.6.1 Favipiravir

Favipiravir is a broad‐spectrum antiviral drug (Table S1), a prodrug of a purine nucleotide. It was used against new influenza in Japan. This drug appears to have a role as an inhibitor of the RNA‐dependent RNA polymerase, which leads to stopping viral replication. It exhibited good efficacy in the treatment of influenza and Ebola virus in preclinical studies and showed activity against other RNA viruses. Regarding the in vitro activity of favipiravir against SARS‐CoV‐2 with the EC_50_ value of 61.88 μM/L in Vero E6 cells and relatively safe use of this agent, in addition to its benefit in early clinical trials, the application of favipiravir for the treatment of COVID‐19 has been recommended. However, more research is needed.^199^, ^200^, ^243^, ^244^ Studies have shown that this drug is effective in disease progression and viral clearance.^245^ Recently, a review of favipiravir trials found that this drug has a negligible effect on mortality in patients with severe symptoms.^246^, ^247^

##### 6.1.6.2 Arbidol

Arbidol is another anti‐influenza drug, and it is a membrane fusion inhibitor. This drug has been approved in Russia and China for treating infections associated with influenza A and B and other arboviruses. Arbidol is prescribed for adults with COVID‐19 disease.^248^, ^249^ One study illustrated that COVID‐19 treatment with Arbidol monotherapy is superior to LPV/RTV.^250^

##### 6.1.6.3 Oseltamivir

Oseltamivir was approved to use against influenza A and influenza B. This drug targets neuraminidase, which is distributed on the surface of the virus. Oseltamivir is under investigation in clinical trials to treat COVID‐19 in combination with other drugs, such as CQ and favipiravir.^237^

#### 6.1.7 Antibiotics

##### 6.1.7.1 Azithromycin

Azithromycin is an antibiotic that belongs to macrolides (Table S1). It is employed to treat a large variety of bacterial infections, such as bronchitis, pneumonia, and *Mycobacterium avium* complex infection. It regulated the pH of endosomes and trans‐Golgi network by acting as an acidotropic lipophilic weak base.^215^ It has also been effective against SARS‐CoV‐2 when combined with HCQ in preliminary clinical trials.^177^, ^200^, ^216^, ^251^ However, a large‐scale randomized clinical trial found no benefit of azithromycin in patients hospitalized with COVID‐19.^252^

#### 6.1.8 Other drugs for COVID‐19

Other therapeutic agents that have been proposed for the treatment of COVID‐19 include copper,^253^ nasal nitric oxide,^254^ Qingwen Baidu Decoction,^255^ sodium chromo‐glycate and palmitoylethanolamide,^256^ bovine lactoferrin,^257^ α‐ketoamides,^258^ mammalian target of rapamycin inhibitors,^259^ 1‐thia‐4‐azaspiro[4.5]decan‐3‐one derivatives,^260^ H_2_S‐producing compounds,^261^ Gemcitabine, lycorine, and oxysophoridine,^262^ Atovaquone, Mebendazole, and Ouabain,^263^ and bruton's tyrosine kinase inhibitors.^264^ These possible drugs need further clinical trials. On the other hand, the auranofin,^265^ eukaryotic initiation factor 4A inhibitor silvestrol,^266^ Liu Shen capsule,^267^ methylprednisolone,^268^ tilorone,^269^ HTCC,^270^ indomethacin and resveratrol,^271^ and naproxen^272^ compounds have been tested, some of which showed good efficacy against SARS‐CoV‐2. However, more comprehensive studies on these drugs should be considered.

### 6.2 Supporting agents

#### 6.2.1 Vitamins

##### 6.2.1.1 Vitamin C

The beneficial effects of vitamin C in improving common colds and pneumonia have been shown previously. Vitamin C decreases the mRNA expression of proinflammatory cytokines in obese patients in vitro. Moreover, the combination of C and E vitamins decreases oxidative stress and reduces the viral load in patients with HIV infection. Furthermore, vitamin C can stimulate the intracellular type I IFN system, which performs the antiviral activity.^273^

##### 6.2.1.2 Vitamin D

This vitamin reduces the risk of viral infection and mortality using different mechanisms, such as decreasing the cytokine storm, regulating adaptive immunity, and stimulating T cell induction. Therefore, it is better for people who are at high risk for COVID‐19 infection to maintain vitamin D at the optimal level in their circulating blood.^273^, ^274^

##### 6.2.1.3 Vitamin E and A

Vitamin E has some acute effects on the immune system, such as improving the activation of natural‐killer, naive T‐lymphocytes, and DC, and inhibiting the production of proinflammatory cytokines, including IL‐1, IL‐6, and TNF. Vitamin A and its metabolites can regulate the innate and adaptive immune system and modulate cytokine production, differentiation, and so on.^274^

##### 6.2.1.4 Convalescent plasma treatment

According to two systematic review studies, convalescent plasma treatment could significantly reduce the viral load and increase the level of neutralizing antibodies in infected patients. Moreover, the general condition of all patients improved after convalescent plasma transfusion. On March 24th, 2020, the FDA approved convalescent plasma transfusion as a treatment option for patients in life‐threatening conditions. However, this treatment strategy has some limitations, such as increased thrombotic event risk and finding volunteer donors with high neutralizing antibody titers, and the optimal dosage.^275^, ^276^ Finally, the Infectious Disease Society of America discourages the use of convalescent plasma in hospitals, saying that there is no evidence yet to support its use in people in the early stages of their infection.^215^

##### 6.2.1.5 Melatonin

Melatonin is a hormone that helps to adjust the cycle of body's sleep‐wake. It is produced in the pineal gland. Melatonin secretion gradually decreases with age. It is thought that this hormone, with its immune‐modulatory and antiviral properties, can be useful as a prophylactic treatment against COVID‐19. However, using this prophylactic treatment may have side effects, such as dizziness, headache, nausea, and sleepiness.^277^

##### 6.2.1.6 Monoclonal antibodies

The efficacy of mAbs, as a promising class of drugs, has been shown against some viral infectious diseases. The target of the mAb is vulnerable sites of viral surface proteins. For example, the human 47D11 mAb targets the full‐length spike proteins of SARS‐CoV and SARS‐CoV‐2 and may be a potential option for the prevention and treatment of COVID‐19. It was suggested that the combination of mAbs and the remdesivir could be an ideal treatment strategy.^278^ Nowadays, mAbs are categorized as widely used treatment against COVID‐19. For example, Regneron won an emergency use authorization for REGEN‐COV in November. The FDA authorized its use for patients with mild to moderate cases who are at high risk of progressing to severe COVID‐19.^279^

###### 6.2.1.6.1 Tocilizumab

Tocilizumab is a humanized mAb IL‐6 receptor antagonist with the protein chemical formula of C_6428_H_9976_N_1720_O_2018_S_42_ that is used to treat rheumatoid arthritis and cytokine release syndrome, a side effect of chimeric antigen receptor T cells anticancer therapy.^199^, ^200^ Therefore, it has been used in some severe cases of COVID‐19, which led to early good results.^199^, ^280^, ^281^ However, another study recommended to use this drug cautiously in severe and critical COVID‐19 cases and did not support the evidence of clinical improvement.^282^ However, WHO recommended this drug for patients with severe or critical COVID‐19 infection.^215^

##### 6.2.1.7 Interferons

The efficacy of IFN therapy was investigated in the SARS‐CoV and MERS‐CoV pandemic. A multicenter randomized open‐label phase 2 trial in COVID‐19 revealed that a multiple combination of an injectable of IFN beta‐1b with LPV‐RTV and ribavirin could reduce virus shedding and hospital stay.^283^, ^284^ However, Su and Jiang declared that the role of IFN in the pathogenesis of SARS‐CoV‐2 is suspicious and may enhance the expression of the ACE2 receptor.^285^ On July 20, the British pharmaceutical company Synairgen announced that an inhaled form of IFN called SNG001 reduced the risk of severe Covid 19 infection in infected patients in a small clinical trial.^286^

##### 6.2.1.8 Corticosteroid

Corticosteroid has two main classes: glucocorticoids and mineralocorticoids. Glucocorticoids reduce inflammation in COVID‐19 patients. The WHO recommends not to use glucocorticoids for COVID‐19 treatment unless for patients with the acute respiratory syndrome. Dexamethasone is a derivative of glucocorticoids. Recent studies showed that Dexamethasone reduces the risk of death in receiving ventilation and requiring oxygen patients. However, this drug does not have a benefit for patients not requiring respiratory support.^287^ The British government estimated that the drug had saved one million lives worldwide.^215^ According to a study, Tocilizumab, another inflammatory agent, is useful in the case of severe and critical COVID‐19 patients and reduced the mortality rate.^288^

### 6.3 Other potential therapies

#### 6.3.1 Aptamer‐based therapy

In recent years, aptamer has attracted the attention of scientists as an alternative to antibiotics. These oligonucleotides (RNA, ssDNA, and peptide molecules) have their specific 3D structure and connect to their targets with high affinity and sensitivity. Since coronaviruses and orthomyxoviridae (influenza viruses) are RNA viruses with a similar infection mode, aptamers designed for influenza viruses can be investigated for SARS‐CoV‐2 therapy. A22 is a DNA‐type aptamer against influenza H5N1. This aptamer reduced the viral load in BALB/c mice up to 90%. Another example of DNA‐type aptamer is C7‐35M against influenza H9N2. This aptamer inhibited viral infections in a dose‐dependent manner.^289^

#### 6.3.2 Chinese folk medicine

From the previous coronavirus outbreaks, it was approved that some Chinese folk medicine has antiviral activity, so therapies used in previous epidemics can help to control the disease caused by SARS‐CoV‐2 and be a starting point for new treatments. Helicase can be considered as a drug target because they are necessary for viral replication. Myricetin, scutellarein, and baicalein are suitable substances that can inhibit the hydrolysis of SARS‐CoV‐1 nsP13. Moreover, it was demonstrated that the biflavonoid and amentoflavone were effective against MERS‐CoV helicase nsP13.^290^

Glycyrrhizin (GL), a triterpene, has various biological functions and can be considered as an antiviral drug for the treatment of COVID‐19. It is assumed that this drug has some antiviral effects and inhibits virus binding to the ACE2 receptor, downregulates proinflammatory cytokines expression, and induces endogenous IFN production.^291^ GL is a frequent component in Chinese folk medicine.^292^

#### 6.3.3 Mesenchymal stem cell therapy

Mesenchymal stem cell (MSC) therapy is broadly used in treating spinal cord injury, type 2 diabetes, autoimmune disease, and some other diseases. Recently, the use of MSCs in the clinical treatment of H5N1 viral infections has also been suggested. MSCs using their immunomodulatory effect to protect alveolar epithelial cells prevent pulmonary fibrosis and cure lung dysfunction.^293^

#### 6.3.4 Small‐interfering RNA

Small‐interfering RNA (siRNA) is a class of double‐stranded and noncoding RNA molecules. The length of this molecule is 20–25 base pairs. It can regulate the expression of genes and so far was implemented for cancer, virus, and genetic disease therapies. In the experience of SARS and MERS, siRNA was effectively used. Therefore, this approach may be useful for COVID‐19 treatment.^294^ However, formulating an effective delivery system is a limitation to this approach.

#### 6.3.5 Bacillus Calmette–Guerin vaccination

It has been described that Bacillus Calmette–Guerin (BCG) vaccination offers broad protection against respiratory infections. It was discovered that countries such as Italy, Netherland, and the United States that do not have universal policies of BCG vaccination have been more severely influenced in comparison with countries that have universal and long‐standing BCG policies.^295^

### 6.4 Vaccine strategy

Designing a vaccine against SARS‐CoV‐2 is challenging. For example, the mutation rate is high in RNA viruses, so the manufactured vaccine may lose its efficacy rapidly. Moreover, an ideal vaccine should decrease transmission, induce herd immunity and long‐lived immunity. In SARS‐CoV‐2, herd immunity would require vaccination of about 67% of the population. Protein subunits, RNA, DNA, nonreplicating vector, replicating vector, inactivated virus, and the attenuated virus are among the main strategies for vaccine design.^296^ Up to now, eight vaccine candidates have achieved regulatory authorization or approval around the globe for full use.^215^ For better understanding, we classified these vaccine strategies into two major platforms: (1) classic vaccine platforms and (2) next‐generation vaccine platforms.

#### 6.4.1 Classic vaccine platforms

We can put virus‐based and protein‐based vaccines in this category. These classical vaccine platforms were successful in the eradication of some diseases, such as smallpox. However, using these platforms is time‐consuming, and the development of a vaccine for this pandemic must be done quickly.^297^ A whole‐inactivated virus is a kind of virus‐based vaccine. This strategy is used against current influenza. This kind of vaccine is safe and easy to prepare.

Moreover, it induced potent serum neutralizing antibodies. However, they may cause disease in highly immunosuppressed individuals. In the category of authorized or approved vaccines, BBIBP‐CorV (Chinese company Sinopharm), CoronaVac (Chinese company Sinovac), and Covaxin (produced by Bharat Biotech) used this strategy. These three mentioned vaccines are developed in China and India (Table 4). The efficacy of BBIBP‐CorV vaccine was reported 78.1% and it is approved in China, Bahrain, UAE, and other countries. The efficacy of CoronaVac varies between 50.65% in Brazil trial to 83.5% in Turkey trial. The injection of this vaccine has approved in China and other countries. The efficacy of Covaxin was reported 77.8% and it was the first COVID‐19 vaccine developed in India to receive emergency approval.^215^ The BIV1‐CovIran vaccine is another vaccine that has been developed based on this strategy. Preclinical study has highlighted the BIV1‐CovIran vaccine as a potential candidate to induce a strong and potent immune response that may be a promising and feasible vaccine to protect against SARS‐CoV‐2 infection.^298^ Emergency use of this vaccine has been issued in Iran and its immunogenicity has been reported at about 90%.

**TABLE 4:** Different authorized or approved vaccine strategies against SARS‐CoV‐2

No.	Brand	Type	Developers	Origin country	Approval
1	BNT162b2	mRNA‐based vaccine	Fosun Pharma, Pfizer, and BioNTech	Multinational	Approved in the United States and other countries Emergency use in E.U. and other countries
2	mRNA‐1273 or Spikevax	mRNA‐based vaccine	Moderna	The United States	Approved in Switzerland Emergency use in the United States, E.U., other countries
3	Sputnik V	Ad26, Ad5	Gamaleya Research Institute	Russia	Emergency use in Russia and other countries
4	Vaxzevria	ChAdOx1	Oxford‐AstraZeneca	British‐Swedish	Approved in Brazil Emergency use in the UK, E.U., and other countries
5	Ad26.COV2.S	Ad26	Johnson & Johnson	The United States	Emergency use in the United States, E.U., and other countries
6	BBIBP‐CorV	Inactivated	Sinopharm‐Wuhan	China	Approved in China, UAE, and Bahrain Emergency use in other countries
7	CoronaVac	Inactivated	Sinovac	China	Approved in China Emergency use in other countries
8	Covaxin	Inactivated	Bharat Biotech	India	Emergency use in India and other countries
9	BIV1‐CovIran	Inactivated	Amirabad Virology Lab, Shifa Pharmed Industrial Group	Iran	Emergency use in Iran

A live‐attenuated virus is another example of classic platforms. The measles, mumps, rubella, and polio vaccines are examples of live‐attenuated virus vaccines. The development of this kind of vaccine may be rapid. However, reversion by mutation or recombination is possible.

The SARS‐CoV‐2 has three surface‐exposed proteins: the M protein (membrane protein), the E protein (envelope protein), and the S protein (spike protein). Among them, the S protein is a promising vaccine candidate. Besides full‐length S protein, the S1 domain, the RBD, and NTD can be considered vaccine candidates. Since S protein is conserved in SARS and MERS, designing a subunit vaccine based on this protein can make cross‐protection. This strategy has been successful in creating the diphtheria, tetanus, pertussis, and hepatitis B vaccines. This kind of vaccine is safe and induces a cellular and humoral immune response. However, they may be too expensive.^299^ In the category of authorized or approved vaccines, EpiVacCorona (Vector Institute) and Novavax are the only protein‐based vaccines developed and approved in Russia and the United States, respectively. The efficacy of EpiVacCorona vaccine is unknown (Table 4).^215^

#### 6.4.2 Next‐generation vaccine platforms

Designing these kinds of vaccines is based on sequence information. Viral vector and nucleic acid‐based vaccines are putting in this category. The viral vector can be a promising vaccine design strategy because of endogenous antigen production and both humoral and cellular immune response stimulation.^296^ Sputnik V (Gamaleya Research Institute) is a nonreplicating viral vector (Ad5 and Ad26) designed in Russia. This vaccine is among authorized or approved vaccines with the efficacy about 91.6% (Table 4). Vaxzevria (manufactured by the British‐Swedish company AstraZeneca) is a vaccine based on ChAdOx1 (chimpanzee adenovirus). The vaccine has an efficacy of 74% against symptomatic COVID‐19 and 100% against severe or critical COVID‐19. Convidecia (Chinese company CanSino Biologics) used Ad5 to develop vaccine against COVID‐19 with the efficacy about 65.28%. Ad26.COV2.S (Johnson & Johnson's vaccine) makes the third coronavirus vaccine available in the United States. The efficacy of this vaccine is about 72% in the United States, 68% in Brazil, and 64% in South Africa.^215^

DNA and RNA vaccine strategies are safe and easy to develop and manufacture. However, the DNA vaccine strategy has some disadvantages, for example, lower immune response and toxicity. On the other hand, RNA vaccines are unstable under physical conditions.^296^ Despite all the mentioned disadvantages, Pfizer‐BioNTech is a successful vaccine developer that uses an mRNA‐based vaccine strategy. This vaccine is currently injected in the UK, Bahrain, Canada, Mexico, the United States, and other countries with the efficacy of 91% (Table 4). The mRNA‐1273 or Spikevax (Boston‐based Company Moderna) is the second vaccine that used this strategy. This vaccine prevents COVID‐19 illness about 93.2% and prevents severe disease about 98.2%.^300^

In addition to vaccines that have been fully approved or licensed for emergency use (discussed above), a number of other vaccines are undergoing the third phase of clinical trials. These vaccines are summarized in Table 5.^215^

**TABLE 5:** The list of vaccine candidates at phase 3 clinical trials

No.	Brand	Type	Developers	Origin country	Approval
1	Convidecia	Ad5	CanSino	China	Approved in China Emergency use in other countries
2	EpiVacCorona and Aurora‐CoV	Protein	Vector Institute	Russia	Approved in Turkmenistan Early use in Russia
3	NVX‐CoV2373	Protein	Novavax	The United States	–
4	Sinopharm	Inactivated	Sinopharm‐Wuhan	China	Approved in China Limited use in UAE

## 7 NANOTECHNOLOGY AND CORONA VIRUSES

### 7.1 Application of nanotechnology to confront coronaviruses

**FIGURE 4:**
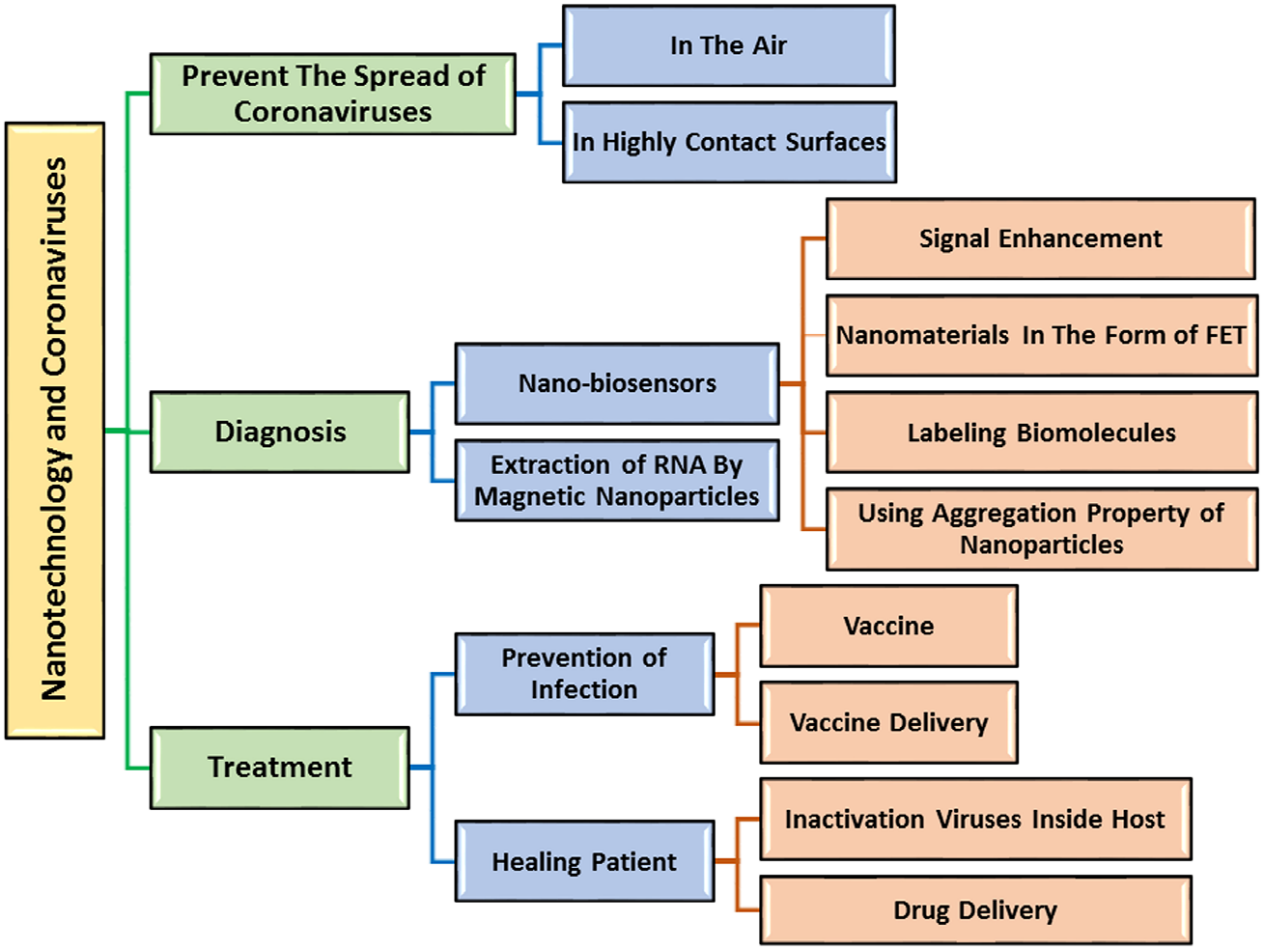
Nanotechnology applications in various stages of confronting coronaviruses

Nanotechnology and nanomaterials are proved to prevent the spread of the virus in the environment, improve diagnosis, assist in vaccine formulation, and facilitate targeted delivery of antiviral drugs (Figure 4).^301^

#### 7.1.1 Prevent the spread of the virus in the environment

One of the most important applications of nanotechnology is to prevent the spread of coronaviruses in the environment outside the body and limit transmission. Transmission of coronaviruses, especially COVID‐19, occurs with two main approaches. One is transmission via droplets scattered in the air through the patient's sneezing and coughing, and the other is contact with the virus‐infected surfaces.^302^ The shelf‐life of the virus on contaminated surfaces depends on the type of body, and the shelf‐life of the virus in the air is estimated to be 3 h.^303^ To prevent the spread of the virus in the air, using face masks for people in public places and using air filters and disinfectants for closed environments such as hospitals are recommended. Depositing a layer of nanomaterials on N95 and FFP3 face masks––which has given them hydrophobicity and self‐disinfection properties,^304^, ^305^ fabricating a replaceable nanoporous membrane to prevent coronavirus entry for N95 face masks,^306^ applying nanocoatings for air filters to create the self‐disinfecting property^307^, ^308^ and using nanodisinfectants in hospitals environment^309^ are examples of the use of nanotechnology to prevent the spread of viruses in the air.

**FIGURE 5:**
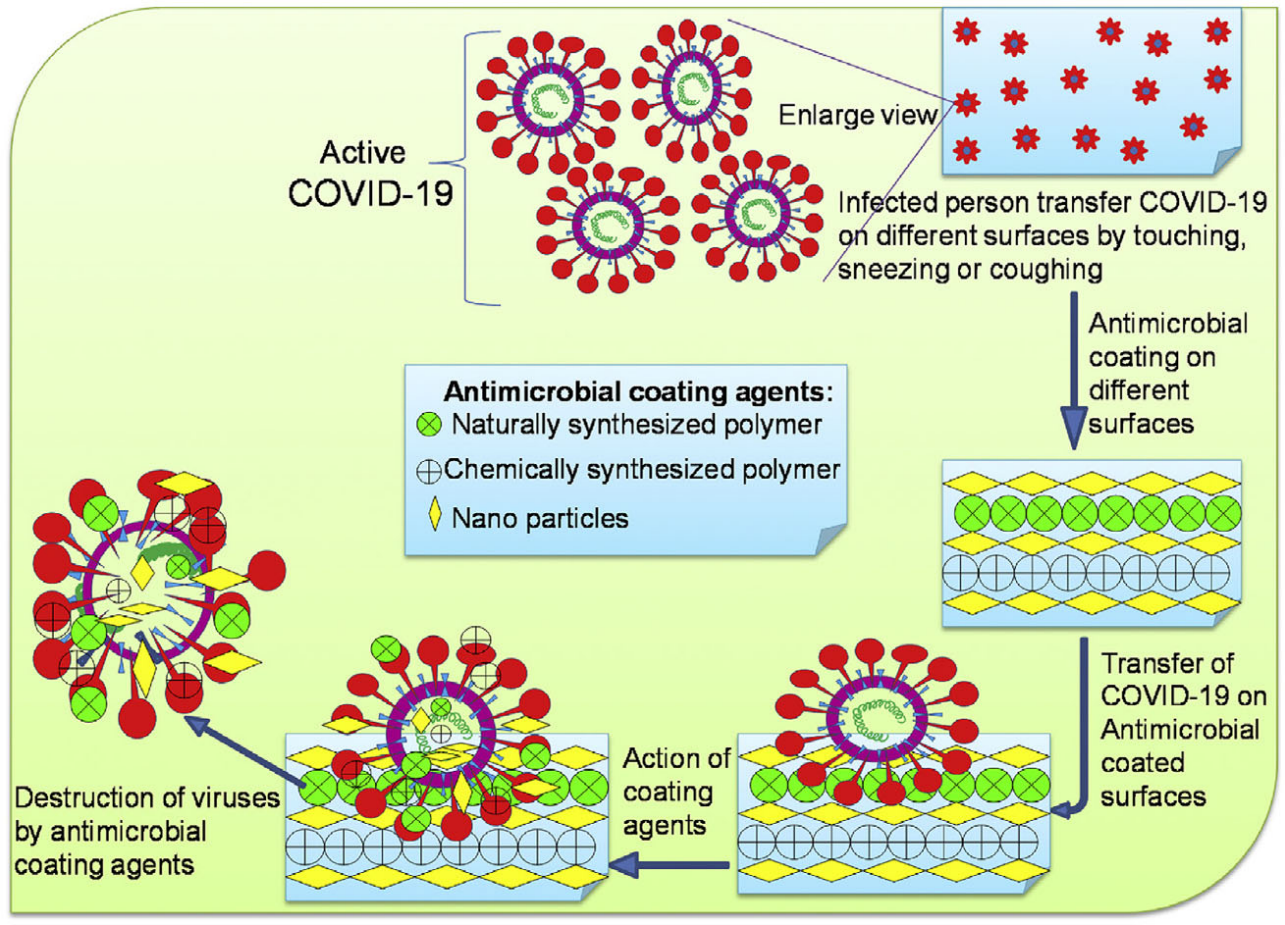
The proposed mechanism to prevent the spread of the COVID‐19 on surfaces through nanocoatings (reproduced from ref. ^309^ with permission from Elsevier)

To prevent virus transmission through contaminated surfaces, the nanocoating was designed using metal nanoparticles, such as Ag, Cu, ZnO, and Ti_2_O_3_.^310^, ^311^ Nanomaterials can be used in polymer paints and coatings for medical devices, walls, and highly contact surfaces.^312^ One of the proposed mechanisms to prevent the spread of the COVID‐19 on surfaces through nanocoatings is shown in Figure 5.^309^ Silver nanoparticles‐based nanodisinfectants are used in hospitals and high‐contact surfaces in public places.^311^

Metal nanoparticles, such as metal oxides and graphene, can be used for these applications due to their antiviral, antibacterial, and antifungal properties. According to the high surface‐to‐volume ratio, small amounts can effectively inactivate the virus.^312^ Antiviral and antimicrobial nanomaterials and their mechanisms of micro‐organisms inactivation are summarized in Table 6.^313^, ^314^

**TABLE 6:** Common antiviral and antibacterial nanomaterials along with their mechanisms

Nanomaterials	Proposed mechanisms	References
Ag nanoparticles (NPs)	The silver ion release Alteration of microbial membrane permeability Interaction of silver nanoparticles with intracellular proteins, especially membrane proteins containing sulfur and microbial DNA Disruption of cell division leading to cell death	^313^, ^314^, ^315^
AuNPs	Contact of AuNPs with viruses has no significant effect on disabling viruses The proposed mechanism is the production of heat by light radiation to gold nanoparticles at a specific wavelength, which is called plasmonic photothermal property, resulting in a change in the virus membrane and a decrease in the binding power of the virus to the surfaces	^313^, ^314^
CuNPs	The Cu ions release reactive oxygen species (ROS) produced from Cu reacting with exogenous hydrogen or molecular oxygen through Fenton‐like or Haber Weiss reactions Damage to envelope proteins and lipids	^313^, ^314^, ^316^
ZnONPs	Intercellular accumulation of nanoparticles Generation of ROS, which accumulates in cell membranes and causes damage to the cell wall and increases membrane permeability Release of Zn ions into the cell and disruption of RNA replication	^313^, ^314^, ^317^
TiO_2_NPs	Production of ROS by UV light due to the photocatalytic properties of TiO_2_NPs Antiviral activity in the absence of light due to cell uptake by NPs and destruction of their membrane	^313^, ^314^
Carbon nanostructures (fullerene, graphene, carbon nanotube [CNT], graphene, and diamond‐like carbon)	Carbon nanostructures damage bacterial cell walls and cell membranes The production of ROS by light radiation due to photochemical activity disrupts cell membranes and damages RNA. It affects the metabolic pathways of energy to inactivate microorganisms in compounds, such as fullerene	^313^, ^314^

### 7.2 Nanotechnology applications in the detection of coronaviruses

Nanomaterials, due to their small size and large surface area, can be effective in sensitivity increase and also quick and easy detection of coronaviruses.^318^

#### 7.2.1 Nanobiosensors

The advantages of combining nanotechnology and biosensors include increased sensitivity and selectivity, improved detection limit, accelerated response, and reduced dimensions of devices, portability, and POC detection.^319^, ^320^, ^321^ Nanomaterials are appropriate for biosensors due to their unique physical and chemical properties and catalytic properties, such as improved electron transfer, biomolecule labeling, and biomolecule adsorption capability.^320^ Table 7 presents some nanobiosensors for the detection of human coronaviruses, such as MERS, SARS, SARS‐CoV2, and also infectious bronchitis virus (IBV) and influenza.

**TABLE 7:** Types of nanobiosensors for the detection of human coronaviruses

Target	Type of biosensor	Nanomaterials	Role of nanomaterials	Methods	Bioreceptor	LOD	Response time	POC (Y/N)	References
COVID‐19	Optical	Gold nanoisland with two dimensions	Functionalizing AuNl with complementary DNA receptors for plasmonic photothermal (PPT) enhancement	Simultaneous use of two techniques: localized surface plasmon resonance (LSPR) and PPT	Nucleic acid	Lower than 0.22 PM	_	N	^322^
	Electrochemical	Gold nanoparticles	Drop cast onto the fluorine doped tin oxide (FTO) electrode as signal amplifier	Potentiometric FTO‐based immunosensor	Antibody	120 FM	20 s	N	^323^
	Electronic	Graphene sheets	Coating graphene sheets of field‐effect transistor (FET) with antibody	FET/voltammetric biosensor	Antibody	2.42 × 10^2^ copies/m (for medical test)	_	Y	^324^
	Colorimetric	Dye‐coated polymer nanoparticles	Dye label for streptavidin	RT‐LAMP coupled with nanoparticles‐based lateral follow biosensor	Nucleic acid	12 copies of each reaction	1 h	Y	^325^
	Optical	Lanthanide‐doped polystyrene nanoparticles (LNPs)	Self‐assembled LNPs for labeling antibody	Fluorescent LFA immunoassay	Nucleocapsid phosphoprotein	_	10 min	N	^326^
	Colorimetric	Gold nanoparticles	AuNPs for labeling antigens	Lateral flow immunoassay for simultaneously detection of IgM and IgG antibodies	COVID‐19 antigen	_	15 min	Y	^327^
MERS	Optical	Nanopillar arrays (NPA) comprise gold nanoislands	NPA enhances light absorption, and functionalized AuNl provides heating for PCR	PPT heating for ultrafast PCR on‐chip	Nucleic acid	0.1 ng.μl^−1^	3 min and 30 s	Y	^328^
	Electrochemical	Gold nanoparticles	Depositing AuNPs on the electrode surface for signal enhancement	Voltammetric immunoassay chip	Spike protein S1	1.0 pg.ml^−1^	20 min	Y	^329^
	Colorimetric	Gold nanoparticles	Functionalized AuNPs with dsDNA self‐assembly	LSPR	Nucleic acid	1 pmol/μl	10 min	Y	^330^
	Colorimetric	Gold nanoparticles	AuNP‐bound polyHRP for signal enhancement	Simultaneous use of ELISA and LFA on 2‐dimensional paper network	HRP‐conjugated antibody label	5 × 10^11^ NA copies/ml	60 min	Y	^331^
	Colorimetric	AgNPs	Using aggregation property of NPs for color changing	Paper‐based colorimetric assay‐based nanoparticles aggregation	Pyrrolidinyl peptide nucleic acid	1.53 nM	_	Y	^332^
SARS	Electronic	Carbon nanotube	Carbon nanotube functionalization with fibronectin‐based protein	FET/conductance biosensor	Nucleic acid	5 nM	_	Y	^333^
	Electronic	In_2_O_3_ nanowire	In_2_O_3_ nanowire functionalization with fibronectin‐based protein	FET/voltammetric biosensor	Antibody mimic proteins (AMPs)	0.6 nM of N protein in 44 μM BSA	10–15 min	N	^334^
Influenza	Optical	CdSe/ZnS quantum dots (QDs)	QDs labeled antibody	Use of QDs as fluorescent labels in LFIAS	Antibody	0.01 ng/ml	15 min	Y	^335^
	Electrochemical	CdS QDs	Influenza virus labeled with QDs	Pulse voltammetry and pulse anodic stripping voltammetry	Glycon‐modified MPs in streptavidin chip	_	_	N	^336^
	Optical	QDs	QD labels excited with UV LEDs	LFA/ratiometric mobile phone fluorescence imaging	Antibody	2 fmol	_	Y	^337^
	Optical	Ag@SiO_2_ nanoparticles	Functionalized NPs as a metal‐enhanced fluorescence sensing platform	Surface plasmon resonance enhancement, which can be transformed into more efficient fluorescence emission	Guanine‐rich anti‐Rha aptamer	2–3.5 ng/ml	30 min	Y	^338^
	Electrochemical	Iron magnetic nanoparticles/gold nanoparticles	MNP‐influenza virus‐AuNPs sandwich	Chronoamperometric biosensor	Anti‐M2 antibody and Fetuin‐A	Less than 16 HAU	160 s	Y	^339^
IBV	Optical	Chiral zirconium QDs and magnetoplasmonic nanoparticles (Fe_3_O_4_@Au)	Formation of nanostructured magnetoplasmonic‐fluorescent with the addition of target	Using the fluorescence properties of immunoconjugated QD‐MPNPs nanohybrids	Antibody	79.15 EID/50 ml	_	N	^340^
	Optical	Molybdenum disulfide 2‐D nanosheet	Fluorescence‐quenching ability of MoS2 when applied to a dye‐labeled antibody	Fluorescent immunosensor performed on cotton thread‐based microfluidic platform	Antibody	4.6×102 EID50 per ml	10 min	Y	^341^

#### 7.2.2 Using nanomaterials for signal enhancement

Metal nanomaterials, in many cases, are used to amplify the output signal of biosensors and increase sensitivity for their unique properties. Due to their ability to accelerate electron transfer^318^ and high conductivity, stability, biocompatibility, and electrical properties related to size, metal nanoparticles can be deposited as a layer on the electrode used in electrochemical biosensors, and their surface can be functionalized with bioreceptors.^323^, ^329^

It is noteworthy that silver and gold nanostructures are applied in optical and colorimetric biosensors as bioreceptors due to their LSPR and PPT properties; otherwise, they are attached to biomolecules to improve the color signal.^322^, ^338^

In this regard, a dual‐functional plasmonic biosensor was designed to detect SARS‐CoV‐2, which operates based on combining the PPT effect and LSPR with gold functionalized nanoislands and complementary DNA receptors. In this project, heat generation due to AuNIs irradiation in plasmonic resonance frequency provides in‐situ hybridization of RdRp‐CoV and cDNA. According to this study, high sensitivity, fast response, and improved accuracy are the salient features of this biosensor.^322^

#### 7.2.3 Implementing nanomaterials in the form of field‐effect transistor

Semiconductor nanomaterials, due to electronic properties, are sensitive to the binding of biomolecules on the surface in the form of FET. Carbon nanotubes,^333^ silicon nanowires,^342^ graphene,^324^ and transition metal dichalcogenide^343^ are typical semiconductor nanostructures for the fabrication of these types of nanosensors that can detect viruses with high sensitivity without the need for viral RNA amplification.^344^

Applying CNT‐functionalized fibronectin based on protein for a conductance biosensor construction to detect the SARS virus is one of the applications of these nanomaterials.^331^ Also, antibody‐coated graphene sheet has led to the development of a voltammetric biosensor for SARS‐Covid 2 detection.^324^

#### 7.2.4 Using nanomaterials for labeling biomolecule

Nanoparticles that attach to biomolecules as labels in optical nanobiosensors have properties such as fluorescence.^341^ In optical biosensors, nanoparticles with fluorescence properties, especially QDs, are used. QDs and AuNPs have also been used as labels in electrochemical biosensors due to their electrical properties.^336^, ^339^ In one study, an IBV detection method using fluorescence and magnetoplasmonic nanocrystals was proposed. In this method, after preparing ZrQDs and paramagnetic nanoparticles and binding IBV antibodies, these nanoparticles come together and form a magnetoplasmonic fluorescent nanohybrids structure in the presence of the target virus. They could be separated from the solution with an external magnet and by measuring their photoluminescence intensity, and the targeted analytic concentration can also be calculated.^340^

Another biosensor for IBV detection was made of MoS_2_ that is a 2‐D nanosheet with a high ability to turn off fluorescence when attached to a dye‐labeled antibody. This immune sensor utilized fluorescence resonance energy transfer between MoS_2_ and the antibody‐bound dye label during antibody–antigen interaction.^341^

#### 7.2.5 Using aggregation property of nanoparticles in colorimetric nanobiosensor

A label‐free colorimetric method for detecting MERS‐CoV has been developed using the optical properties of gold nanoparticles that lead to color change in the presence and absence of the target molecule. In a recent study surface of the gold, nanoparticles were coated with two thiolated single‐stranded DNAs with strong Au–S interaction. Hybridization of target DNA with ssDNA on gold nanoparticles' surface leads to AuNPs aggregation and changes the solution's color through an LSPR shift.^330^

**FIGURE 6:**
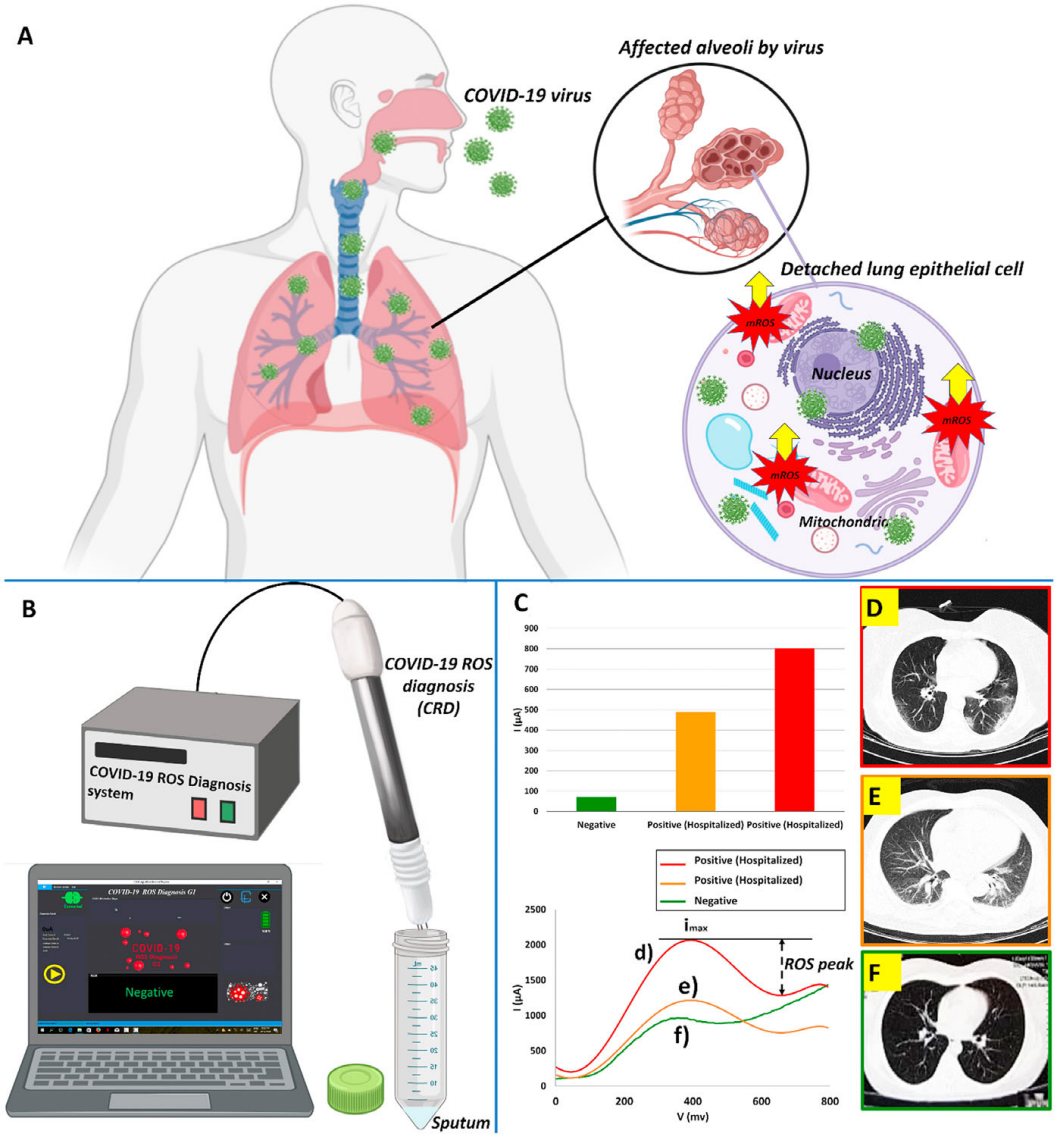
Schematic illustration of: (A) COVID‐19 side effect in lung host cells (the virus amplifies viral replications by inducing overproduction of mitochondrial ROS); (B) the COVID‐19 ROS diagnosis system (consists of three needle electrodes coated by functionalized MWCNTs); (C) electrochemical reaction and cyclic voltammetry cathodic peaks from two different patients; (D and E) the CT‐scan of the patient showed more distinctive hazy patches; (F) a normal candidate lung CT‐scan (reproduced from ref. ^345^ with permission from Elsevier)

Other applications of nanotechnology include nanomaterials used as a tracer in biochemical sensors. One of the biosensors for detection of CoV‐19 used a multiwall carbon nanotube (MWCNT) to cover the electrode heads in the biosensor. The biosensor was based on electrochemical measurements of ROS released in a virus‐infected lung. MWCNT is known as an electrochemical superoxidant selective tracer, such as H_2_O_2_/ROS. The amount of electrical change is characterized due to the ROS reaction on the surface of the electrode and is transferred to the working electrode by the counter electrode, and this system measures the released ROS by cyclic voltammetry procedure (Figure 6). The detection time is less than 30 s in this biosensor.^345^

#### 7.2.6 Extraction of RNA by magnetic nanoparticles

RT‐PCR test requires the extraction of high‐purity viral RNA from complex samples.^346^, ^347^, ^348^ This method is entirely dependent on the amount of target so that if the extraction efficiency is low, the received signal as a response will be weak and may lead to false‐negative results.^349^ The silica‐based spin column is a standard method for extracting RNA from nasopharyngeal cells.^346^, ^349^ This method protocol has several steps, such as sample prelysis, centrifugation, washing, and consumption of toxic organic solvents, which is time‐consuming and requires plenty of operators in the laboratory. Due to low extraction efficiency and operator endangerment, it is essential to use a fast and automatic technique to extract viral RNA.^346^, ^350^, ^351^ Surface functionalized magnetic nanoparticles (MNPs) can extract viral RNA as an alternative method.^352^ This method has fewer steps, does not require any centrifuge step, fast and straightforward, and can also be operated manually and automatically.

Additionally, it has high extraction efficiency and reduces false‐negative results. In this method, the surface of MNPs is functionalized by groups with strong interaction with nucleic acids and RNA molecules are efficiently and rapidly adsorbed from the lysis solution on the surface of MNPs. The extracted RNA can enter the detection process by RT‐PCR without MNPs wash or after washing with buffer and separated nanoparticle.^346^, ^350^, ^353^

In one study, by using the poly amino ester‐functionalized Fe_3_O_4_ NPs, viral RNA molecules were adsorbed on the surface of NPs via carboxylic groups and pcMNPs–RNA complex was used directly in RT‐PCR for the detection of SARS‐CoV‐2.^346^

### 7.3 Nanotechnology‐based treatments

Today, various nanotechnology‐based products have been produced, developed, and made available to the public in order to fight COVID‐19.

#### 7.3.1 Vaccine development

The development of viral or peptide vaccines against various diseases, such as infectious diseases, has been a medical advancement.^354^, ^355^, ^356^ Nanotechnology has the potential for the development of COVID‐19 drug delivery due to enormous advantages included:

1. Small size and morphology of the nanoparticles that can deliver the drug to physiologically sites and reduce immune response by reticular endothelial cells. ^357^
2. The big surface‐to‐volume ratio of nanoparticles that make an increase of drug loading.^358^
3. The ability to cross negatively charge membranes because of surface charge modification of nanoparticles.^359^, ^360^

According to many studies, both humoral and cell‐mediated immunity play a beneficial role in SARS‐CoV infection.^361^, ^362^ Both adaptive systems (T cells and B cells) and innate immune systems (macrophages, monocytes, and neutrophils) at the cellular level can be targeted by nanoparticles. Modulating APCs using nanoparticles could be very significant, especially for COVID‐19 vaccine strategies.^363^, ^364^ The capability of nanoparticles to deliver antigen to DCs by enhancing antigen presentation and several other mechanisms can boost T cell immunity.^365^ Various nanoparticle‐based mechanisms for altering the induction of the immune response have been described in many studies. Nanoparticles have shown many benefits for improving the efficacy and safety of the vaccine approach, including their ability to deliver molecular adjuvants. Also, in some cases, the nanomaterials themselves have inherent adjuvant property for the loaded antigens.^366^ Table 8 summarizes the applications of different nanoparticles against COVID‐19, SARS, and influenza.

**TABLE 8:** Applications of nanoparticle‐based vaccine‐like polymeric NPs, peptide‐based NPs, inorganic NPs, and so on against COVID‐19, SARS, and influenza

Target	Nanomaterials	Antigen	Mechanism	Adjuvant	References
COVID‐19	Au NPs	Swine transmissible gastroenteritis	Rise of the peritoneal macrophages respiratory action and plasma IFN‐γ level	Au NPs	^367^
	Au NPs	SARS‐CoV	Induction of IgG response	Au NPs	^368^
	Ferritin‐based NPs	MERS‐CoV (RBD antigen)	Initiation of CD4^+^ T cells and IFN‐α TNF‐γ reactions	–	^369^
	Spike protein NPs	MERS‐CoV	Initiation of better titers of neutralizing antibody and Th2 immune response with no initiation of Th1 immune reaction	Aluminum	^370^
	Hollow polymeric NPs	MERS‐CoV (RBD antigen)	Initiation of elevated amounts of humoral reactions and IgG2a antibodies with no initiation of lung eosinophilic immunopathology	–	^371^
SARS	Polyprotein 1a (pp1a) of the human SARS coronavirus	Proteins 10 and 11 (hereafter NSP10)	Used as a novel self‐assembling system for NPs	Adavax (Squalene based)	^372^
Influenza	Poly(lactic‐coglycolic acid)	Inactivated SwIV H1N2	(PLGA‐KAg) capsule vaccine in cells in vitro	–	^373^
	Au NPs	Antigen extracellular domain of the M2 protein (M2e)	Use gold nanoparticle and its conjugated CpG oligodeoxynucleotide 1826 to document rises in the breathing action of splenic lymphocytes in the respirational action of peritoneal macrophages and in the creation of proinflammatory cytokines (IL‐6 and IFN‐γ).	CpG	^374^
	Virus‐like particle	Hemagglutinin	Extremely preserved multiple ectodomains of matrix protein 2 (M2e5x VLP) of influenza virus stimulate broad cross‐protection by M2‐specific humoral and cellular immune reactions	^−^	^375^

#### 7.3.2 Therapeutic development

Nanotechnology offers many solutions to prevent COVID‐19. In this section, we will review some solutions in this field.

##### 7.3.2.1 Use of carbon quantum dots for COVID‐19 treatment

In one study, carbon quantum dots (CQDs) were first synthesized and post modified with boronic acid ligands. The second generation of anti‐HCoV nanomaterials contains CQDs derived from 4‐aminophenylboronic acid without any further modification, which has an EC50 lower than 5.2 ± 0.7 μg ml^−1^. The main mechanism of action of these CQDs is inhibition of HCoV‐229 entry, which occurs due to the interaction of its functional groups with HCoV‐229 entry receptors, so that an equally large inhibition activity is observed in the viral replication phase.^376^

##### 7.3.2.2 Mesenchymal stem cells and management of COVID‐19 pneumonia (LIFNano)

Leukemia inhibitory factor (LIF) is essential to resist the cytokine storm in the lungs in the period of viral pneumonia.^377^, ^378^ Even though MSCs produce LIF as a remedy, this fails because of cell‐based nature while bringing a prohibitive cost burden. Based on nanotechnology strategies, synthetic stem cells “LIFNano” can be used with a thousand times boost in effectiveness than soluble LIF.^379^ In EAE, a preclinical version of multiple sclerosis, LIFNano‐based therapy navigated paralysis in 1 week, a timeline in accordance with that revealed for advantageous impacts in COVID‐19 pneumonia using MSC treatment. Earlier experiments using neural stem cells (NSCs) to cure experimental autoimmune encephalomyelitis (EAE) indicated advantages that were only related to NSC‐derived LIF. While a developing replacement to cell‐based therapy, like LIFNano, provide the requirement for a large quantity and off‐the‐shelf medicinal agents able to regenerate injured tissues and restrain cytokine storm in pneumonia. Worldwide delivery is straightforward utilizing low‐volume vials optional delivery routes, including inhalation or intravenous or both.^380^

## 8 IN‐SILICO STUDIES AND COMPUTATIONAL BIOLOGY APPROACHES IN COVID‐19

### 8.1 Background

The silico study allows the researcher to perform an utterly complete classification of various parameters, thereby providing further suggestions or predictions of workable results.^381^ Thus, bioinformatics resources have become a powerful tool for optimizing and analyzing the interaction between target proteins and antiviral candidates, searching for new antiviral compounds by reducing the time and costs associated with laboratory evaluation.^382^

### 8.2 Computational biology and bioinformatics in fundamental studies of SARS‐CoV‐2

In‐silico findings and computational studies have greatly accelerated the process of fundamental studies of SARS‐CoV‐2, such as virus genomics and proteomics, structural studies, evolutionary studies, and phylogenetics. These necessary studies provide an introduction to the rapid development of research in the diagnosis, prevention, and treatment of COVID‐19, each of which is facilitated by in‐silico approaches. In the field of genomics and proteomics of SARS‐CoV‐2, various databases and servers provided services, which can be seen in Table S2.

A simple search in the NCBI database with SARS‐CoV‐2 keyword to date revealed that one genome (Accession number: NC_045512.2), 25,501 nucleotide sequences, one taxonomy, 12 genes, 135 GEO data sets, 265,862 protein sequences, 457 protein structures, and 3711 case clinical trials have been recorded, which indicates an explosion of information in this case.

Some servers have also been instrumental in the structural modeling of SARS‐CoV‐2 proteins or their derivatives, such as Phyre 2 for ab initio modeling, PSIPRED for prediction of secondary structure, and ITASSER and PEPFOLD3 for predicting the third structure (3D) of proteins and peptides and the SWISS‐MODEL for the homology modeling of sequences whose structures are unknown.^383^, ^384^, ^385^, ^386^, ^387^

The design of specific primers (by bioinformatics tools) to perform molecular detection tests on nasopharyngeal and oropharyngeal specimens was one of the first in‐silico achievements in COVID‐19. From the very beginning, qRT‐PCR was the basis for this type of diagnosis that the primers based on ORF1ab and N genes were designed.^388^ In the diagnosis of the SARS‐CoV‐2, false‐negative responses have a high rate and diagnostic accuracy is about 30–50%, which is due to mutations and the spread of different types of viruses. Accordingly, more detailed studies are being conducted in this field today, in which bioinformatics studies play an essential role.^389^, ^390^ For example, to select the best regions for primers design, conserve areas in cDNAs derived from different SARS‐CoV‐2 variant genomes can be used, which are obtained after alignment with bioinformatics methods, such as FASTA, Basic Local Alignment Search Tool, and multiple sequence alignments, such as Clustal Omega and T‐Coffee.^391^, ^392^, ^393^, ^394^, ^395^ However, this strategy assumes that DNA sequences have general characteristics and require prior information and, usually, several tests for accurate diagnosis.^396^

Deep learning (DL) methods based on the artificial neural networks (ANNs) machine learning (ML) algorithm can have many applications in bioinformatics studies, drug design, and biomedical analysis.^397^, ^398^, ^399^ DL methods are essential strategies for classifying viral DNA and cDNA sequences without the need for prior information.^400^, ^401^ In April 2020, a study was published in the Bulletin of the World Health Organization that used a combination of DL techniques, viromics, and primer design with bioinformatics tools to design specific primers for accurate SARS‐CoV‐2 detection. Their results showed that designed primers are very specific and significantly increase the accuracy of the diagnosis of COVID‐19 compared to previous studies.^391^ In addition to genome and proteome analysis by ML algorithms, a method called nCOVnet uses an ANN‐based DL strategy to screen and rapidly analyze X‐ray images of the lungs of people suspected of having COVID‐19 for definitive diagnosis.^402^

### 8.3 Immunoinformatics technics and in‐silico studies in vaccine design for COVID‐19

In the prevention and design of vaccines or multiepitope construct with diagnostic value, immunoinformatics studies are critical in identifying the best immune‐dominant epitopes of different SARS‐CoV‐2 proteins.^403^, ^404^, ^405^ In the early 1990s, a genome‐based vaccine design or reverse vaccinology approach was introduced. Today, bioinformatics methods have facilitated the development of this approach.^406^ In many in‐silico studies to COVID‐19 vaccine design, epitopes associated with T helper and Cytotoxic T lymphocytes (CTL) epitopes were placed next to B cell epitopes by specific linkers so that the final construct could stimulate both humoral and cell‐mediated immunity and produce a better immune response.^405^, ^406^ Many servers and software have been useful in this area.^403^, ^404^, ^405^

In this regard, in addition to actual databases, such as NCBI, PDB, Expasy, and PDB sum, other servers provide immune‐informatics services to design multiepitope constructs. For example, Vaxijenserver^407^ for predicting protein antigenicity, Immune Epitope Database (IEDB)^403^, ^404^, ^405^, ^408^ for mapping, forecasting, and evaluating epitopes, and NetCTL1.2 for designing CTL cell epitopes are useful.^404^, ^409^ Awareness of Human leukocyte antigen (HLA) types involved in the immune response to COVID‐19 is essential for vaccine design. TepiTool from IEDB server can enable this.^410^ Epitool kit is a suitable server for designing Treg cell epitopes.^404^, ^411^ ABCpred and BepiPred linear epitope prediction tools can create linear B cell epitopes,^412^, ^413^, ^414^ while Ellipro is used to design conformational (discontinuous) B cell epitopes.^415^ AlgPred and Allertop servers are used to predict the allergenicity of designed sequences.^416^, ^417^ Usually, the final construct's antigenicity is rechecked with Vaxijen v2.0 or ANTIGENpro tools.^407^, ^418^ The SOLPro server predicts the solubility of the protein construct.^419^ C‐ImmSim online server is used to simulate the immune response against the final design.^420^ Phyre 2 and Galaxy refine server can predict the structure, modify, and evaluate the quality of the spatial structure of the final multiepitope construct.^421^, ^422^ ProtParm can predict the physicochemical properties of the designed construct. The most important of these properties are isoelectric pH (pI), molecular weight (Mw), amino acid content, hydrophobicity, instability index, and Grand average of hydropathicity index (GRAVY).^423^ RAMPAGE and SWISS‐MODEL can be used to predict the placement of protein backbone residues according to their torsion (dihedral) angles in the permitted or unauthorized areas of the Ramachandran plot.^404^, ^424^ The ProSA‐web server can also be used for the analysis and final validation of the structure of a designed protein construct.^425^

To evaluate the interaction affinity and binding stability of vaccine candidate construct with some important immune‐receptors, such as Toll‐like receptors (TLRs) and Human leukocyte antigens (HLAs), molecular docking studies and molecular dynamics (MD) simulations are very important and based on their results can be selected as the best vaccine candidates.^404^, ^426^, ^427^ HADDOCK and ClusPro are useful methods for performing molecular docking between two proteins or a peptide with one protein.^428^, ^429^, ^430^ GROMACS and Yet Another Scientific Artificial Reality Application (YASARA) are also two servers suitable for MD.^431^, ^432^ Eventually, at the IEDB, Population Coverage Analysis Tool can be performed to determine what percentage of the population responds appropriately to the epitopes.^404^ The population coverage analysis tool calculates the fraction of individuals predicted to respond to a given set of epitopes with known MHC restrictions.^433^

### 8.4 Chemoinformatics and computational bioinformatics for drug discovery and drug design to combat COVID‐19

Computer‐aided drug discovery is a promising strategy for developing novel drugs and specific targets to combat diseases, such as COVID‐19; this issue requires understanding of the structure of coronavirus and various target proteins.^434^ Regarding the urgency of therapeutic measures for the COVID‐19 pandemic and because drug discovery is a time‐consuming process, the use of available drugs with FDA approval is preferred due to the need for less cost and time.^435^ In‐silico medicine repurposing represents an accurate way to speed up the screening of existing drugs with FDA approval to find a therapeutic option for COVID‐19.^436^, ^437^

For this purpose, databases and servers related to drug and chemical compounds, such as DrugBank (https://go.drugbank.com/) and PubChem (https://pubchem.ncbi.nlm.nih.gov/), were used as beneficial resources. Also, chemoinformatics and in‐silico approaches, such as Monte Carlo‐based quantitative structure–activity relationship (QSAR), SARS‐CoV‐2 Genome Annotation (NCBI server), Homology Modeling (SWISS‐MODEL), virtual screening (VS), molecular docking (Large‐Scale Docking by supercomputer), MD, prediction of physicochemical properties, toxicity, immunogenicity, pharmacokinetic/pharmacodynamics parameters, and drug‐likeness analysis, have been widely used in drug discovery and design for COVID‐19.^438^, ^439^

Virtual screening has become a popular method for novel drug discovery and drug repurposing.^393^, ^394^ Structure‐based and ligand‐based drug discovery and design are two critical subgroups of this type of virtual screening.^440^, ^441^ Usually, in silico, drug screening for COVID‐19 is performed based on structures of viral proteins (spike protein, viral helicase, 3C‐like protease [3CL^pro^], and RNA‐dependent RNA polymerase order) and human ACE2 receptor.^439^ In drug discovery, in addition to HADDOCK and ClusPro mentioned above, AutoDock Vina is widely used for molecular docking protein targets and small molecules.^442^

The big data obtained from high‐throughput screening and the high number of drug candidates have shown that ML algorithms, such as support vector machine, ANN, random forests, discriminant analysis, and so on, have become a fundamental tool for drug designing and discovery.^423^, ^443^ ML techniques can be used to model QSAR or quantitative structure–property relationships and develop artificial intelligence programs that accurately predict in silico how chemical modifications might influence biological behavior.^441^, ^442^ Many physicochemical properties of drugs, such as toxicity, metabolism, drug–drug interactions, and carcinogenesis, have been effectively modeled by QSAR techniques.^443^, ^444^

Finally, on the importance of in‐silico drug discovery and repurposing, it should be noted that due to the lack of specific FDA‐approved drugs against SARS‐CoV‐2, currently available antiviral drugs, such as remdesivir, favipiravir, CQ, and so on, are prescribed to COVID‐19 patients.

## 9 CONCLUSION AND FUTURE PERSPECTIVE

This article reviews the general and biological characteristics of the virus, including genomics, proteomics, receptors, immunopathology, host immune response, and pathogen immune evasion strategies. Then, various molecular methods, including nucleic acid‐ and protein‐based as well as the POC and X‐ray‐based methodologies examined in the diagnosis of COVID‐19, were discussed. In the following, two pathways for manipulation of the mice for SARS‐CoV‐2 studies were described, including producing hACE2 mice for the development of vaccines and other potential antiviral therapies and knockout of other genes to mimic disease. Then, we framed the symptom effects and clinical properties of COVID‐19 on various conditions. Symptoms and illnesses were included in abdominal symptoms, hypertension, diabetes, renal dysfunction, cancer, and so on. In the next section, multiple treatments for this disease were described, including therapeutic and antiviral agents and inhibitors, supporting agents, and other potential therapies. Then, the applications of nanotechnology in the identification, diagnosis, and treatment of coronaviruses and, finally, in‐silico studies and biocomputational approaches in COVID‐19 were reviewed. According to the extensive studies that are being done around the world during the outbreak of this disease, there is no doubt that effective drugs and vaccines will be made for this disease, although some of them have entered the consumer market. The use of new technologies, such as nanotechnology, will also facilitate the continuation, diagnosis, and treatment of COVID‐19. Nevertheless, we should confess that the unexpected time‐to‐time mutations and designation of new variants of COVID‐19 make it, indeed, a near‐to‐impossible task to look at the future ahead of treatment and mortality prevention of COVID‐19 from an explicit perspective.

## FUNDING

Not applicable.

## CONFLICT OF INTEREST

There is no conflict of interest to declare.

## AUTHOR CONTRIBUTIONS

H.A.M.A. and R.E.‐K. conceived the study, collected the literatures, and drafted the manuscript. Corresponding authors, including V.K., H.M., and M.M., provided their corrective comments and tips. All authors collaborated to write the article. P.Z., M.R.S., and M.M. edited the revised version of the manuscript. All authors approved this manuscript for publication.

## ETHICS APPROVAL

Not applicable.

## Supporting information

SUPPORTING INFORMATION

## Data Availability

The data included in this study are available upon request from the corresponding authors.

## References

[1] World Health Organization (WHO). WHO Coronavirus (COVID‐19) Dashboard. https://covid19.who.int/. Accessed January 11, 2022.

[2] Tay MZ , Poh CM , Rénia L , MacAry PA , Ng LFP . The trinity of COVID‐19: immunity, inflammation and intervention. Nat Rev Immunol. 2020;20(6):363‐374. 32346093 10.1038/s41577-020-0311-8 PMC7187672

[3] Abd El‐Aziz TM , Stockand JD . Recent progress and challenges in drug development against COVID‐19 coronavirus (SARS‐CoV‐2) — an update on the status. Infect Genet Evol. 2020;83:104327. 32320825 10.1016/j.meegid.2020.104327 PMC7166307

[4] Daniloski Z , Jordan TX , Ilmain JK , et al. The spike D614G mutation increases SARS‐CoV‐2 infection of multiple human cell types. eLife. 2021;10:e65365. 33570490 10.7554/eLife.65365 PMC7891930

[5] Korber B , Fischer WM , Gnanakaran S , et al. Tracking changes in SARS‐CoV‐2 spike: evidence that D614G increases infectivity of the COVID‐19 virus. Cell. 2020;182(4):812‐827. 32697968 10.1016/j.cell.2020.06.043 PMC7332439

[6] Prasaath Sastha KR , Arul B , Kothai R . Understanding COVID‐19 – the pandemic of 2020. Int J Res Pharm Sci. 2020;11:94‐102.

[7] Zhang H , Dai H , Xie X . Solid organ transplantation during the COVID‐19 pandemic. Front Immunol. 2020;11:1392. 32612614 10.3389/fimmu.2020.01392 PMC7308422

[8] Corman VM , Rabenau HF , Adams O , et al. SARS‐CoV‐2 asymptomatic and symptomatic patients and risk for transfusion transmission. Transfusion. 2020;60(6):1119‐1122. 32361996 10.1111/trf.15841 PMC7267331

[9] Kallem VR , Sharma D . COVID 19 in neonates. J Matern Fetal Neonatal Med. 2020;18:1‐9. 10.1080/14767058.2020.1759542 32419544

[10] Wigginton KR , Boehm AB . Environmental engineers and scientists have important roles to play in stemming outbreaks and pandemics caused by enveloped viruses. Environ Sci Technol. 2020;54(7):3736‐3739. 32207922 10.1021/acs.est.0c01476

[11] Qu G , Li X , Hu L , Jiang G . An imperative need for research on the role of environmental factors in transmission of novel coronavirus (COVID‐19). Environ Sci Technol. 2020;54(7):3730‐3732. 32202420 10.1021/acs.est.0c01102

[12] Renu K , Prasanna PL , Valsala Gopalakrishnan A . Coronaviruses pathogenesis, comorbidities and multi‐organ damage — a review. Life Sci. 2020;255:117839. 32450165 10.1016/j.lfs.2020.117839 PMC7243768

[13] Dömling A , Gao L . Chemistry and biology of SARS‐CoV‐2. Chem. 2020;6(6):1283‐1295. 32529116 10.1016/j.chempr.2020.04.023 PMC7243793

[14] Holstein B . Coronavirus 101. J Nurse Pract. 2020;16(6):416‐419. 32292300 10.1016/j.nurpra.2020.03.021 PMC7146654

[15] Liu B , Li M , Zhou Z , Guan X , Xiang Y . Can we use interleukin‐6 (IL‐6) blockade for coronavirus disease 2019 (COVID‐19)‐induced cytokine release syndrome (CRS)? J Autoimmun. 2020;111:102452. 32291137 10.1016/j.jaut.2020.102452 PMC7151347

[16] Han H , Ma Q , Li C , et al. Profiling serum cytokines in COVID‐19 patients reveals IL‐6 and IL‐10 are disease severity predictors. Emerg Microbes Infect. 2020;9(1):1123‐1130. 32475230 10.1080/22221751.2020.1770129 PMC7473317

[17] Li Q , Tang B , Bragazzi NL , Xiao Y , Wu J . Modeling the impact of mass influenza vaccination and public health interventions on COVID‐19 epidemics with limited detection capability. Math Biosci. 2020;325:108378. 32507746 10.1016/j.mbs.2020.108378 PMC7229764

[18] Johns Hopkins Center for Health Security. SARS‐CoV‐2 Genetics. https://www.centerforhealthsecurity.org. Accessed April 16, 2020.

[19] Tang X , Wu Ch , Li X , et al. On the origin and continuing evolution of SARS‐CoV‐2. Natl Sci Rev. 2020;7(6):1012‐1023. 34676127 10.1093/nsr/nwaa036 PMC7107875

[20] Liu SL , Saif LJ , Weiss SR , Su L . No credible evidence supporting claims of the laboratory engineering of SARS‐CoV‐2. Emerg Microbes Infect. 2020;9(1):505‐507. 32102621 10.1080/22221751.2020.1733440 PMC7054935

[21] Bar‐On YM , Flamholz A , Phillips R , Milo R . SARS‐CoV‐2 (COVID‐19) by the numbers. eLife. 2020;9:e57309. 32228860 10.7554/eLife.57309 PMC7224694

[22] V'kovski P , Kratzel A , Steiner S , Stalder H , Thiel V . Coronavirus biology and replication: implications for SARS‐CoV‐2. Nat Rev Microbiol. 2021;19(3):155‐170. 33116300 10.1038/s41579-020-00468-6 PMC7592455

[23] Zhou P , Yang XL , Wang XG , et al. A pneumonia outbreak associated with a new coronavirus of probable bat origin. Nature. 2020;579(7798):270‐273. 32015507 10.1038/s41586-020-2012-7 PMC7095418

[24] Wu F , Zhao S , Yu B , et al. A new coronavirus associated with human respiratory disease in China. Nature. 2020;580(7803):E7. 32296181 10.1038/s41586-020-2202-3 PMC7608129

[25] Yang J , Yan Y , Zhong W . Application of omics technology to combat the COVID‐19 pandemic. MedComm. 2021;2(3):381‐401. 34766152 10.1002/mco2.90 PMC8554664

[26] Jackson B , Boni MF , Bull MJ , et al. Generation and transmission of interlineage recombinants in the SARS‐CoV‐2 pandemic. Cell. 2021;184(20):5179‐5188. 34499854 10.1016/j.cell.2021.08.014 PMC8367733

[27] World Health Organization (WHO). Guidance for Surveillance of SARS‐CoV‐2 Variants: Nterim Guidance. https://www.who.int/publications/i/item/WHO_2019‐nCoV_surveillance_variants. Accessed August 9, 2021.

[28] Washington NL , Gangavarapu K , Zeller M , et al. Emergence and rapid transmission of SARS‐CoV‐2 B.1.1.7 in the United States. Cell. 2021;184(10):2587‐2594. 33861950 10.1016/j.cell.2021.03.052 PMC8009040

[29] Wu A , Wang L , Zhou HY , et al. One year of SARS‐CoV‐2 evolution. Cell Host Microbe. 2021;29(4):503‐507. 33676588 10.1016/j.chom.2021.02.017 PMC7903908

[30] Li Q , Nie J , Wu J , et al. SARS‐CoV‐2 501Y.V2 variants lack higher infectivity but do have immune escape. Cell. 2021;184(9):2362‐2371. 33735608 10.1016/j.cell.2021.02.042 PMC7901273

[31] Cao Y , Yisimayi A , Bai Y , et al. Humoral immune response to circulating SARS‐CoV‐2 variants elicited by inactivated and RBD‐subunit vaccines. Cell Res. 2021;31(7):732‐741. 34021265 10.1038/s41422-021-00514-9 PMC8138844

[32] Wang P , Casner RG , Nair MS , et al. Increased resistance of SARS‐CoV‐2 variant P.1 to antibody neutralization. Cell Host Microbe. 2021;29(5):747‐751. 33887205 10.1016/j.chom.2021.04.007 PMC8053237

[33] Lopez Bernal J , Andrews N , Gower C , et al. Effectiveness of Covid‐19 vaccines against the B.1.617.2 (delta) variant. N Engl J Med. 2021;385(7):585‐594. 34289274 10.1056/NEJMoa2108891 PMC8314739

[34] Wu B , Zhang H , Wang YC , et al. Sequencing on an imported case in China of COVID‐19 Delta variant emerging from India in a cargo ship in Zhoushan, China. J Med Virol. 2021;93(12):6828‐6832. 34314048 10.1002/jmv.27239 PMC8426989

[35] Xuemei H , Hong W , Xiangyu P , Guangwen L , Xiawei W . SARS‐CoV‐2 Omicron variant: characteristics and prevention. MedComm. 2021;2(4):838‐845. 10.1002/mco2.110 PMC8693031 34957469

[36] Zahradník J , Marciano S , Shemesh M , et al. SARS‐CoV‐2 variant prediction and antiviral drug design are enabled by RBD in vitro evolution. Nat Microbiol. 2021;6(9):1188‐1198. 34400835 10.1038/s41564-021-00954-4

[37] Liu C , Ginn HM , Dejnirattisai W , et al. Reduced neutralization of SARS‐CoV‐2 B.1.617 by vaccine and convalescent serum. Cell. 2021;184(16):4220‐4236. 34242578 10.1016/j.cell.2021.06.020 PMC8218332

[38] McCallum M , Bassi J , De Marco A , et al. SARS‐CoV‐2 immune evasion by the B.1.427/B.1.429 variant of concern. Science. 2021;373(6555):648‐654. 34210893 10.1126/science.abi7994 PMC9835956

[39] Deng X , Garcia‐Knight M , Khalid M , et al. Transmission, infectivity, and neutralization of a spike L452R SARS‐CoV‐2 variant. Cell. 2021;184(13):3426‐3437. 33991487 10.1016/j.cell.2021.04.025 PMC8057738

[40] Spratt A , Kannan S , Woods L , et al. Evolution, correlation, structural impact and dynamics of emerging SARS‐CoV‐2 variants. Comput Struct Biotechnol J. 2021;19:3799‐3809. 34188776 10.1016/j.csbj.2021.06.037 PMC8225291

[41] Liu J , Liu Y , Xia H , et al. BNT162b2‐elicited neutralization of B.1.617 and other SARS‐CoV‐2 variants. Nature. 2021;596(7871):273‐275. 34111888 10.1038/s41586-021-03693-y

[42] Zhou H , Dcosta BM , Samanovic MI , Mulligan MJ , Landau NR , Tada T . B.1.526 SARS‐CoV‐2 variants identified in New York City are neutralized by vaccine‐elicited and therapeutic monoclonal antibodies. mBio. 2021;27:e0138621. 10.1128/mBio.01386-21 PMC8406170 34311587

[43] Edara VV , Lai L , Sahoo MK , et al. Infection and vaccine‐induced neutralizing antibody responses to the SARS‐CoV‐2 B.1.617.1 variant. bioRxiv. Published online May 10, 2021. 10.1101/2021.05.09.443299 PMC8279090 34233096

[44] Chen L , Lu L , Choi C , et al. Impact of SARS‐CoV‐2 variant associated RBD mutations on the susceptibility to serum antibodies elicited by COVID‐19 infection or vaccination. Clin Infect Dis. 2021:ciab656. 34309648 10.1093/cid/ciab656

[45] Tablizo FA , Kim KM , Lapid CM , et al. Genome sequencing and analysis of an emergent SARS‐CoV‐2 variant characterized by multiple spike protein mutations detected from the Central Visayas Region of the Philippines. medRxiv. Published online March 6, 2021. 10.1101/2021.03.03.21252812

[46] Padilla‐Rojas C , Jimenez‐Vasquez V , Hurtado V , et al. Genomic analysis reveals a rapid spread and predominance of lambda (C.37) SARS‐COV‐2 lineage in Peru despite circulation of variants of concern. J Med Virol. 2021;93(12):6845‐6849. 34370324 10.1002/jmv.27261 PMC8427000

[47] Chakraborty C , Bhattacharya M , Sharma AR , Lee SS , Agoramoorthy G . SARS‐CoV‐2 Brazil variants in Latin America: more serious research urgently needed on public health and vaccine protection. Ann Med Surg (Lond). 2021;66:102428. 34109031 10.1016/j.amsu.2021.102428 PMC8178066

[48] Heurich A , Hofmann‐Winkler H , Gierer S , Liepold T , Jahn O , Pöhlmann S . TMPRSS2 and ADAM17 cleave ACE2 differentially and only proteolysis by TMPRSS2 augments entry driven by the severe acute respiratory syndrome coronavirus spike protein. J Virol. 2014;88(2):1293‐1307. 24227843 10.1128/JVI.02202-13 PMC3911672

[49] Di Nardo M , van Leeuwen G , Loreti A , et al. A literature review of 2019 novel coronavirus (SARS‐CoV2) infection in neonates and children. Pediatr Res. 2021;89(5):1101‐1108. 32679582 10.1038/s41390-020-1065-5

[50] Jeffers SA , Tusell SM , Gillim‐Ross L , et al. CD209L (L‐SIGN) is a receptor for severe acute respiratory syndrome coronavirus. Proc Natl Acad Sci U S A. 2004;101(44):15748‐15753. 15496474 10.1073/pnas.0403812101 PMC524836

[51] Amraei R , Yin W , Napoleon MA , et al. CD209L/L‐SIGN and CD209/DC‐SIGN act as receptors for SARS‐CoV‐2. ACS Cent Sci. 2021;7(7):1156‐1165. 34341769 10.1021/acscentsci.0c01537 PMC8265543

[52] Wang K , Chen W , Zhang Z , et al. CD147‐spike protein is a novel route for SARS‐CoV‐2 infection to host cells. Signal Transduct Target Ther. 2020;5(1):283. 33277466 10.1038/s41392-020-00426-x PMC7714896

[53] Ulrich H , Pillat MM . CD147 as a target for COVID‐19 treatment: suggested effects of azithromycin and stem cell engagement. Stem Cell Rev Rep. 2020;16(3):434‐440. 32307653 10.1007/s12015-020-09976-7 PMC7167302

[54] Espinosa JM . Down syndrome and COVID‐19: a perfect storm? Cell Rep Med. 2020;1(2):100019. 32501455 10.1016/j.xcrm.2020.100019 PMC7252041

[55] Yang L , Liu S , Liu J , et al. COVID‐19: immunopathogenesis and immunotherapeutics. Sig Transduct Target Ther. 2020;5(1):128. 10.1038/s41392-020-00243-2 PMC7381863 32712629

[56] Del Valle DM , Kim‐Schulze S , Huang HH , et al. An inflammatory cytokine signature predicts COVID‐19 severity and survival. Nat Med. 2020;26(10):1636‐1643. 32839624 10.1038/s41591-020-1051-9 PMC7869028

[57] Prompetchara E , Ketloy C , Palaga T . Immune responses in COVID‐19 and potential vaccines: lessons learned from SARS and MERS epidemic. Asian Pac J Allergy Immunol. 2020;38(1):1‐9. 32105090 10.12932/AP-200220-0772

[58] Zhong J , Tang J , Ye C , Dong L . The immunology of COVID‐19: is immune modulation an option for treatment? Lancet Rheumatol. 2020;2(7):e428‐e436. 32835246 10.1016/S2665-9913(20)30120-X PMC7239618

[59] Cao X . COVID‐19: immunopathology and its implications for therapy. Nat Rev Immunol. 2020;20(5):269‐270. 32273594 10.1038/s41577-020-0308-3 PMC7143200

[60] İnandıklıoğlu N , Akkoc T . Immune responses to SARS‐CoV, MERS‐CoV and SARS‐CoV‐2. Adv Exp Med Biol. 2020;1288:5‐12. 32514817 10.1007/5584_2020_549

[61] Lei X , Dong X , Ma R , et al. Activation and evasion of type I interferon responses by SARS‐CoV‐2. Nat Commun. 2020;11(1):3810. 32733001 10.1038/s41467-020-17665-9 PMC7392898

[62] Jiang C , Li X , Ge C , et al. Molecular detection of SARS‐CoV‐2 being challenged by virus variation and asymptomatic infection. J Pharm Anal. 2021;11(3):257‐264. 33815862 10.1016/j.jpha.2021.03.006 PMC7997641

[63] Liu R , Fu A , Deng Z , Li Y , Liu T . Promising methods for detection of novel coronavirus SARS‐CoV‐2. View. 2020;1(1):e4. 10.1002/viw2.4 PMC7169335 38607796

[64] Ravi N , Cortade DL , Ng E , Wang SX . Diagnostics for SARS‐CoV‐2 detection: a comprehensive review of the FDA‐EUA COVID‐19 testing landscape. Biosens Bioelectron. 2020;165:112454. 32729549 10.1016/j.bios.2020.112454 PMC7368663

[65] Li C , Ren L . Recent progress on the diagnosis of 2019 novel coronavirus Transbound Emerg Dis. 2020;67(4):1485‐1491. 32395897 10.1111/tbed.13620 PMC7272792

[66] Nelson PP , Rath BA , Fragkou PC , Antalis E , Tsiodras S , Skevaki C . Current and future point‐of‐care tests for emerging and new respiratory viruses and future perspectives. Front Cell Infect Microbiol. 2020;10:181. 32411619 10.3389/fcimb.2020.00181 PMC7202255

[67] Huang C , Wen T , Shi FJ , Zeng XY , Jiao YJ . Rapid detection of IgM antibodies against the SARS‐CoV‐2 virus via colloidal gold nanoparticle‐based lateral‐flow assay. ACS Omega. 2020;5(21):12550‐12556. 32542208 10.1021/acsomega.0c01554 PMC7241732

[68] Soni N , Pai P , Krishna Kumar GR , Prasad V , Dasgupta S , Bhadra B . A flow virometry process proposed for detection of SARS‐CoV‐2 and large‐scale screening of COVID‐19 cases. Future Virol. 2020;10.2217/fvl‐2020‐0141.

[69] Ward S , Lindsley A , Courter J , Assa'ad A. Clinical testing for COVID‐19. J Allergy Clin Immunol. 2020;146(1):23‐34. 32445839 10.1016/j.jaci.2020.05.012 PMC7237919

[70] Zhou Y , Pei F , Ji M , et al. Sensitivity evaluation of 2019 novel coronavirus (SARS‐CoV‐2) RT‐PCR detection kits and strategy to reduce false negative. PLoS One. 2020;15(11):e0241469. 33206690 10.1371/journal.pone.0241469 PMC7673793

[71] Pujadas E , Ibeh N , Hernandez MM , et al. Comparison of SARS‐CoV‐2 detection from nasopharyngeal swab samples by the Roche cobas 6800 SARS‐CoV‐2 test and a laboratory‐developed real‐time RT‐PCR test. J Med Virol. 2020;92(9):1695‐1698. 32383179 10.1002/jmv.25988 PMC7267546

[72] Fu Y , Li B , Liu G . Rapid and efficient detection methods of pathogenic swine enteric coronaviruses. Appl Microbiol Biotechnol. 2020;104(14):6091‐6100. 32430534 10.1007/s00253-020-10645-5 PMC7235545

[73] Singh B , Datta B , Ashish A , Dutta G . A comprehensive review on current COVID‐19 detection methods: from lab care to point of care diagnosis. Sens Int. 2021;2:100119. 34766062 10.1016/j.sintl.2021.100119 PMC8302821

[74] Yoo HM , Kim IH , Kim S . Nucleic acid testing of SARS‐CoV‐2. Int J Mol Sci. 2021;22(11):6150. 34200331 10.3390/ijms22116150 PMC8201071

[75] Yang W , Dang X , Wang Q , et al. Rapid detection of SARS‐CoV‐2 using reverse transcription RT‐LAMP method. medRxiv. Published online March 3, 2020. 10.1101/2020.03.02.20030130

[76] Amaral C , Antunes W , Moe E , et al. A molecular test based on RT‐LAMP for rapid, sensitive and inexpensive colorimetric detection of SARS‐CoV‐2 in clinical samples. Sci Rep. 2021;11(1):16430. 34385527 10.1038/s41598-021-95799-6 PMC8361189

[77] Lau YL , Ismail IB , Mustapa NIB , et al. Development of a reverse transcription recombinase polymerase amplification assay for rapid and direct visual detection of severe acute respiratory syndrome coronavirus 2 (SARS‐CoV‐2). PLoS One. 2021;16(1):e0245164. 33406112 10.1371/journal.pone.0245164 PMC7787525

[78] Yu L , Wu S , Hao X , et al. Rapid detection of COVID‐19 coronavirus using a reverse transcriptional loop‐mediated isothermal amplification (RT‐LAMP) diagnostic platform. Clin Chem. 2020;66(7):975‐977. 32315390 10.1093/clinchem/hvaa102 PMC7188121

[79] Chen S , He C , Li Y , Li Z , Melançon CE . A computational toolset for rapid identification of SARS‐CoV‐2, other viruses and microorganisms from sequencing data. Brief Bioinform. 2021;22(2):924‐935. 33003197 10.1093/bib/bbaa231 PMC7543257

[80] Dara M , Talebzadeh M . CRISPR/Cas as a potential diagnosis technique for COVID‐19. Avicenna J Med Biotechnol. 2020;12(3):201‐202. 32695284 PMC7368118

[81] Kellner MJ , Koob JG , Gootenberg JS , Abudayyeh OO , Zhang F . SHERLOCK: nucleic acid detection with CRISPR nucleases. Nat Protoc. 2020;15(3):1311. 32005984 10.1038/s41596-020-0302-z

[82] Joung J , Ladha A , Saito M , et al. Point‐of‐care testing for COVID‐19 using SHERLOCK diagnostics. medRxiv. Published online May 8, 2020. 10.1101/2020.05.04.20091231

[83] Carter LJ , Garner LV , Smoot JW , et al. Assay techniques and test development for COVID‐19 diagnosis. ACS Cent Sci. 2020;6(5):591‐605. 32382657 10.1021/acscentsci.0c00501 PMC7197457

[84] John G , Sahajpal NS , Mondal AK , et al. Next‐generation sequencing (NGS) in COVID‐19: a tool for SARS‐CoV‐2 diagnosis, monitoring new strains and phylodynamic modeling in molecular epidemiology. Curr Issues Mol Biol. 2021;43(2):845‐867. 34449545 10.3390/cimb43020061 PMC8929009

[85] Wu Q , Suo C , Brown T , Wang T , Teichmann SA , Bassett AR . INSIGHT: a population‐scale COVID‐19 testing strategy combining point‐of‐care diagnosis with centralized high‐throughput sequencing. Sci Adv. 2021;7(7):eabe5054. 33579697 10.1126/sciadv.abe5054 PMC7880595

[86] Hossain A , Reis AC , Rahman S , Salis HM . A Massively Parallel COVID‐19 Diagnostic Assay for Simultaneous Testing of 19200 Patient Samples. Google Docs; 2020.

[87] Yasumizu Y , Hara A , Sakaguchi S , Ohkura N . VIRTUS: a pipeline for comprehensive virus analysis from conventional RNA‐seq data. Bioinformatics. 2021;37(10):1465‐1467. 33017003 10.1093/bioinformatics/btaa859 PMC7745649

[88] Mathur G , Mathur S . Antibody testing for COVID‐19. Am J Clin Pathol. 2020;154(1):1‐3. 32412044 10.1093/ajcp/aqaa082 PMC7239247

[89] Lambert‐Niclot S , Cuffel A , Le Pape S , et al. Evaluation of a rapid diagnostic assay for detection of SARS‐CoV‐2 antigen in nasopharyngeal swabs. J Clin Microbiol. 2020;58(8):e00977‐20. 32404480 10.1128/JCM.00977-20 PMC7383555

[90] Santaella‐Tenorio J . SARS‐CoV‐2 diagnostic testing alternatives for Latin America. Colomb Med (Cali). 2020;51(2):e4272. 33012887 10.25100/cm.v51i2.4272 PMC7518727

[91] Krüttgen A , Cornelissen CG , Dreher M , Hornef M , Imöhl M , Kleines M . Comparison of four new commercial serologic assays for determination of SARS‐CoV‐2 IgG. J Clin Virol. 2020;128:104394. 32416599 10.1016/j.jcv.2020.104394 PMC7189838

[92] Theel ES , Slev P , Wheeler S , Couturier MR , Wong SJ , Kadkhoda K . The role of antibody testing for SARS‐CoV‐2: is there one? J Clin Microbiol. 2020;58(8):e00797‐20. 32350047 10.1128/JCM.00797-20 PMC7383527

[93] Ng KW , Faulkner N , Cornish GH , et al. Preexisting and de novo humoral immunity to SARS‐CoV‐2 in humans. Science. 2020;370(6522):1339‐1343. 33159009 10.1126/science.abe1107 PMC7857411

[94] Djaileb A , Charron B , Jodaylami MH , et al. Cross‐validation of ELISA and a portable surface plasmon resonance instrument for IgG antibody serology with SARS‐CoV‐2 positive individuals. Analyst. 2021;146(15):4905‐4917. 34250530 10.1039/d1an00893e

[95] Lapuente D , Maier C , Irrgang P , et al. Rapid response flow cytometric assay for the detection of antibody responses to SARS‐CoV‐2. Eur J Clin Microbiol Infect Dis. 2021;40(4):751‐759. 33078221 10.1007/s10096-020-04072-7 PMC7572153

[96] Wen T , Huang C , Shi FJ , et al. Development of a lateral flow immunoassay strip for rapid detection of IgG antibody against SARS‐CoV‐2 virus. Analyst. 2020;145(15):5345‐5352. 32568341 10.1039/d0an00629g

[97] Hoy CFO , Kushiro K , Yamaoka Y , Ryo A , Takai M . Rapid multiplex microfiber‐based immunoassay for anti‐MERS‐CoV antibody detection. Sens Bio‐Sens Res. 2019;26:100304. 10.1016/j.sbsr.2019.100304 PMC7104066 32289017

[98] Rissin DM , Kan CW , Campbell TG , et al. Single‐molecule enzyme‐linked immunosorbent assay detects serum proteins at subfemtomolar concentrations. Nat Biotechnol. 2010;28(6):595‐599. 20495550 10.1038/nbt.1641 PMC2919230

[99] Shan D , Johnson JM , Fernandes SC , et al. N‐protein presents early in blood, dried blood and saliva during asymptomatic and symptomatic SARS‐CoV‐2 infection. Nat Commun. 2021;12(1):1931. 33771993 10.1038/s41467-021-22072-9 PMC7997897

[100] Cinquanta L , Fontana DE , Bizzaro N . Chemiluminescent immunoassay technology: what does it change in autoantibody detection? Auto Immun Highlights. 2017;8(1):9. 28647912 10.1007/s13317-017-0097-2 PMC5483212

[101] Nicol T , Lefeuvre C , Serri O , et al. Assessment of SARS‐CoV‐2 serological tests for the diagnosis of COVID‐19 through the evaluation of three immunoassays: two automated immunoassays (Euroimmun and Abbott) and one rapid lateral flow immunoassay (NG Biotech). J Clin Virol. 2020;129:104511. 32593133 10.1016/j.jcv.2020.104511 PMC7295485

[102] Mullett WM , Lai EPC , Yeung JM . Surface plasmon resonance‐based immunoassays. Methods. 2000;22(1):77‐91. 11020321 10.1006/meth.2000.1039

[103] Yola ML , Eren T , Atar N . Molecular imprinted nanosensor based on surface plasmon resonance: application to the sensitive determination of amoxicillin. Sens Actuators B: Chem. 2014;195:28‐35.

[104] Nguyen T , Duong Bang D , Wolff A . 2019 novel coronavirus disease (COVID‐19): paving the road for rapid detection and point‐of‐care diagnostics. Micromachines. 2020;11(3):306. 10.3390/mi11030306 PMC7142866 32183357

[105] Pulia MS , O'Brien TP , Hou PC , Schuman A , Sambursky R . Multi‐tiered screening and diagnosis strategy for COVID‐19: a model for sustainable testing capacity in response to pandemic. Ann Med. 2020;52(5):207‐214. 32370561 10.1080/07853890.2020.1763449 PMC7877955

[106] Grant BD , Anderson CE , Williford JR , et al. SARS‐CoV‐2 coronavirus nucleocapsid antigen‐detecting half‐strip lateral flow assay toward the development of point of care tests using commercially available reagents. Anal Chem. 2020;92(16):11305‐11309. 32605363 10.1021/acs.analchem.0c01975

[107] FIND Diagnosis for All. Test Directory. https://www.finddx.org/test-directory/?_type_of_technology=immunoassay. Accessed November 23, 2021.

[108] Gao X , Liu X , Zhang Y , Wei Y , Wang Y . Rapid and visual detection of porcine delta coronavirus by recombinase polymerase amplification combined with a lateral flow dipstick. BMC Vet Res. 2020;16(1):130. 32381014 10.1186/s12917-020-02341-3 PMC7203717

[109] Udugama B , Kadhiresan P , Kozlowski HN , et al. Diagnosing COVID‐19: the disease and tools for detection. ACS Nano. 2020;14(4):3822‐3835. 32223179 10.1021/acsnano.0c02624

[110] da Silva SJR , Silva CTAD , Guarines KM , et al. Clinical and laboratory diagnosis of SARS‐CoV‐2, the virus causing COVID‐19. ACS Infect Dis. 2020;6(9):2319‐2336. 32786280 10.1021/acsinfecdis.0c00274

[111] Sidiq Z , Hanif M , Dwivedi KK , Chopra KK . Laboratory diagnosis of novel corona virus (2019‐nCoV)—present and the future. Indian J Tuberc. 2020;67(4S):S128‐S131. 33308658 10.1016/j.ijtb.2020.09.023 PMC7527305

[112] Yong SK , Su PC , Yang YS . Molecular targets for the testing of COVID‐19. Biotechnol J. 2020;15(6):e2000152. 32419272 10.1002/biot.202000152 PMC7267081

[113] Liu J , Yu H , Zhang S . The indispensable role of chest CT in the detection of coronavirus disease 2019 (COVID‐19). Eur J Nucl Med Mol Imaging. 2020;47(7):1638‐1639. 32246209 10.1007/s00259-020-04795-x PMC7118704

[114] Zhang J , Xie Y , Pang G , et al. Viral pneumonia screening on chest X‐rays using confidence‐aware anomaly detection. IEEE Trans Med Imaging. 2021;40(3):879‐890. 33245693 10.1109/TMI.2020.3040950 PMC8544953

[115] Xu B , Xing Y , Peng J , et al. Chest CT for detecting COVID‐19: a systematic review and meta‐analysis of diagnostic accuracy. Eur Radiol. 2020;30(10):5720‐5727. 32415585 10.1007/s00330-020-06934-2 PMC7227176

[116] Takayama K . In vitro and animal models for SARS‐CoV‐2 research. Trends Pharmacol Sci. 2020;41(8):513‐517. 32553545 10.1016/j.tips.2020.05.005 PMC7260555

[117] Singh A , Singh RS , Sarma P , et al. A comprehensive review of animal models for coronaviruses: SARS‐CoV‐2, SARS‐CoV, and MERS‐CoV. Virol Sin. 2020;35(3):290‐304. 32607866 10.1007/s12250-020-00252-z PMC7324485

[118] Sharun K , Tiwari R , Patel SK , et al. Coronavirus disease 2019 (COVID‐19) in domestic animals and wildlife: advances and prospects in the development of animal models for vaccine and therapeutic research. Hum Vaccin Immunother. 2020;16(12):3043‐3054. 32915100 10.1080/21645515.2020.1807802 PMC8641595

[119] Shi J , Wen Z , Zhong G , et al. Susceptibility of ferrets, cats, dogs, and other domesticated animals to SARS‐coronavirus 2. Science. 2020;368(6494):1016‐1020. 32269068 10.1126/science.abb7015 PMC7164390

[120] Lakdawala SS , Menachery VD . The search for a COVID‐19 animal model. Science. 2020;368(6494):942‐943. 32467379 10.1126/science.abc6141

[121] Deb B , Shah H , Goel S . Current global vaccine and drug efforts against COVID‐19: pros and cons of bypassing animal trials. J Biosci. 2020;45(1):82. 32554907 10.1007/s12038-020-00053-2 PMC7291183

[122] Lutz C , Maher L , Lee C , Kang W . COVID‐19 preclinical models: human angiotensin‐converting enzyme 2 transgenic mice. Hum Genomics. 2020;14(1):20. 32498696 10.1186/s40246-020-00272-6 PMC7269898

[123] Sun J , Zhuang Z , Zheng J , et al. Generation of a broadly useful model for COVID‐19 pathogenesis, vaccination, and treatment. Cell. 2020;182(3):734‐743. 32643603 10.1016/j.cell.2020.06.010 PMC7284240

[124] Sun SH , Chen Q , Gu HJ , et al. A mouse model of SARS‐CoV‐2 infection and pathogenesis. Cell Host Microbe. 2020;28(1):124‐133. 32485164 10.1016/j.chom.2020.05.020 PMC7250783

[125] Tseng CT , Huang C , Newman P , et al. Severe acute respiratory syndrome coronavirus infection of mice transgenic for the human angiotensin‐converting enzyme 2 virus receptor. J Virol. 2007;81(3):1162‐1173. 17108019 10.1128/JVI.01702-06 PMC1797529

[126] Yoshikawa N , Yoshikawa T , Hill T , et al. Differential virological and immunological outcome of severe acute respiratory syndrome coronavirus infection in susceptible and resistant transgenic mice expressing human angiotensin‐converting enzyme 2. J Virol. 2009;83(11):5451‐5465. 19297479 10.1128/JVI.02272-08 PMC2681954

[127] McCray PB Jr , Pewe L , Wohlford‐Lenane C , et al. Lethal infection of K18‐hACE2 mice infected with severe acute respiratory syndrome coronavirus. J Virol. 2007;81(2):813‐821. 17079315 10.1128/JVI.02012-06 PMC1797474

[128] Yang XH , Deng W , Tong Z , et al. Mice transgenic for human angiotensin‐converting enzyme 2 provide a model for SARS coronavirus infection. Comp Med. 2007;57(5):450‐459. 17974127

[129] Menachery VD , Yount BL Jr , Sims AC , et al. SARS‐like WIV1‐CoV poised for human emergence. Proc Natl Acad Sci U S A. 2016;113(11):3048‐3053. 26976607 10.1073/pnas.1517719113 PMC4801244

[130] Wang J , Kaplan N , Wysocki J , et al. The ACE2‐deficient mouse: a model for a cytokine storm‐driven inflammation. FASEB J. 2020;34(8):10505‐10515. 32725927 10.1096/fj.202001020R PMC7323146

[131] Ni W , Yang X , Yang D , et al. Role of angiotensin‐converting enzyme 2 (ACE2) in COVID‐19. Crit Care. 2020;24:422. 32660650 10.1186/s13054-020-03120-0 PMC7356137

[132] Stopsack KH , Mucci LA , Antonarakis ES , Nelson PS , Kantoff PW . TMPRSS2 and COVID‐19: serendipity or opportunity for intervention? Cancer Discov. 2020;10(6):779‐782. 32276929 10.1158/2159-8290.CD-20-0451 PMC7437472

[133] Sakai K , Ami Y , Tahara M , et al. The host protease TMPRSS2 plays a major role in in vivo replication of emerging H7N9 and seasonal influenza viruses. J Virol. 2014;88(10):5608‐5616. 24600012 10.1128/JVI.03677-13 PMC4019123

[134] Hogan RJ , Gao G , Rowe T , et al. Resolution of primary severe acute respiratory syndrome‐associated coronavirus infection requires Stat1. J Virol. 2004;78(20):11416‐11421. 15452265 10.1128/JVI.78.20.11416-11421.2004 PMC521834

[135] Frieman MB , Chen J , Morrison TE , et al. SARS‐CoV pathogenesis is regulated by a STAT1 dependent but a type I, II and III interferon receptor independent mechanism. PLoS Pathog. 2010;6(4):e1000849. 20386712 10.1371/journal.ppat.1000849 PMC2851658

[136] World Health Organization (WHO) . Novel Coronavirus (2019‐nCoV). Situation Report – 1. https://www.who.int/docs/default-source/coronaviruse/situation-reports/20200121-sitrep-1-2019-ncov.pdf. Accessed January 21, 2020.

[137] Zhang J , Wang S , Xue Y . Fecal specimen diagnosis 2019 novel coronavirus‐infected pneumonia. J Med Virol. 2020;92(6):680‐682. 32124995 10.1002/jmv.25742 PMC7228355

[138] Ren JG , Li DY , Wang CF , et al. Positive RT‐PCR in urine from an asymptomatic patient with novel coronavirus 2019 infection: a case report. Infect Dis (Lond). 2020;52(8):571‐574. 32420777 10.1080/23744235.2020.1766105

[139] Chen N , Zhou M , Dong X , et al. Epidemiological and clinical characteristics of 99 cases of 2019 novel coronavirus pneumonia in Wuhan, China: a descriptive study. Lancet. 2020;395(10223):507‐513. 32007143 10.1016/S0140-6736(20)30211-7 PMC7135076

[140] Rivett L , Sridhar S , Sparkes D , et al. Screening of healthcare workers for SARS‐CoV‐2 highlights the role of asymptomatic carriage in COVID‐19 transmission. eLife. 2020;9:e58728. 32392129 10.7554/eLife.58728 PMC7314537

[141] Sorbello M , El‐Boghdadly K , Di Giacinto I , et al. The Italian coronavirus disease 2019 outbreak: recommendations from clinical practice. Anaesthesia. 2020;75(6):724‐732. 32221973 10.1111/anae.15049

[142] Leva E , Morandi A , Sartori A , Macchini F , Berrettini A , Manzoni G . Correspondence from Northern Italy about our experience with COVID‐19. J Pediatr Surg. 2020;55(5):985‐986. 32278542 10.1016/j.jpedsurg.2020.03.028 PMC7195317

[143] Lazzerini M , Barbi E , Apicella A , Marchetti F , Cardinale F , Trobia G . Delayed access or provision of care in Italy resulting from fear of COVID‐19. Lancet Child Adolesc Health. 2020;4(5):e10‐e11. 32278365 10.1016/S2352-4642(20)30108-5 PMC7146704

[144] Mahmoudi S , Mehdizadeh M , Shervin Badv R , et al. The coronavirus disease 2019 (COVID‐19) in children: a study in an Iranian children's referral hospital. Infect Drug Resist. 2020;13:2649‐2655. 32801803 10.2147/IDR.S259064 PMC7406067

[145] Yokoo P , Fonseca E , Filho MO , Chate RC , Szarf G & Baroni RH Abdominal symptoms as an initial manifestation of COVID‐19 infection: report of two cases. Preprint (Version 1). Research Square; 2020.

[146] Kanne JP . Chest CT findings in 2019 novel coronavirus (2019‐nCoV) infections from Wuhan, China: key points for the radiologist. Radiology. 2020;295(1):16‐17. 32017662 10.1148/radiol.2020200241 PMC7233362

[147] Preckel B , Schultz MJ , Vlaar AP , et al. Update for anaesthetists on clinical features of COVID‐19 patients and relevant management. J Clin Med. 2020;9(5):1495. 10.3390/jcm9051495 PMC7291059 32429249

[148] Esler M , Esler D . Can angiotensin receptor‐blocking drugs perhaps be harmful in the COVID‐19 pandemic? J Hypertens. 2020;38(5):781‐782. 32195824 10.1097/HJH.0000000000002450

[149] Saeed U , Sellevoll HB , Young VS , Sandbaek G , Glomsaker T , Mala T . Covid‐19 may present with acute abdominal pain. Br J Surg. 2020;107(7):e186‐e187. 32343396 10.1002/bjs.11674 PMC7267330

[150] Zhang S , Li H , Huang S , You W , Sun H . High‐resolution computed tomography features of 17 cases of coronavirus disease 2019 in Sichuan province, China. Eur Respir J. 2020;55(4):2000334. 32139463 10.1183/13993003.00334-2020 PMC7098481

[151] Pranata R , Lim MA , Huang I , Raharjo SB , Lukito AA . Hypertension is associated with increased mortality and severity of disease in COVID‐19 pneumonia: a systematic review, meta‐analysis and meta‐regression. J Renin Angiotensin Aldosterone Syst. 2020;21(2):1470320320926899. 32408793 10.1177/1470320320926899 PMC7231906

[152] Huang C , Wang Y , Li X , et al. Clinical features of patients infected with 2019 novel coronavirus in Wuhan, China. Lancet. 2020;395(10223):497‐506. 31986264 10.1016/S0140-6736(20)30183-5 PMC7159299

[153] Wang D , Hu B , Hu C , et al. Clinical characteristics of 138 hospitalized patients with 2019 novel coronavirus‐infected pneumonia in Wuhan, China. JAMA. 2020;323(11):1061‐1069. 32031570 10.1001/jama.2020.1585 PMC7042881

[154] Zhou F , Yu T , Du R , et al. Clinical course and risk factors for mortality of adult inpatients with COVID‐19 in Wuhan, China: a retrospective cohort study. Lancet. 2020;395(10229):1054‐1062. 32171076 10.1016/S0140-6736(20)30566-3 PMC7270627

[155] Zhang JJ , Dong X , Cao YY , et al. Clinical characteristics of 140 patients infected with SARS‐CoV‐2 in Wuhan, China. Allergy. 2020;75(7):1730‐1741. 32077115 10.1111/all.14238

[156] Yang X , Yu Y , Xu J , et al. Clinical course and outcomes of critically ill patients with SARS‐CoV‐2 pneumonia in Wuhan, China: a single‐centered, retrospective, observational study. Lancet Respir Med. 2020;8(5):475‐481. 32105632 10.1016/S2213-2600(20)30079-5 PMC7102538

[157] Wu Z , McGoogan JM . Characteristics of and important lessons from the coronavirus disease 2019 (COVID‐19) outbreak in China: summary of a report of 72 314 cases from the Chinese Center for Disease Control and Prevention. JAMA. 2020;323(13):1239‐1242. 32091533 10.1001/jama.2020.2648

[158] Khan M , Khan H , Khan S , Nawaz M . Epidemiological and clinical characteristics of coronavirus disease (COVID‐19) cases at a screening clinic during the early outbreak period: a single‐centre study. J Med Microbiol. 2020;69(8):1114‐1123. 32783802 10.1099/jmm.0.001231 PMC7642977

[159] Guan W , Ni Z , Hu Y , et al. Clinical characteristics of coronavirus disease 2019 in China. N Engl J Med. 2020;382(18):1708‐1720. 32109013 10.1056/NEJMoa2002032 PMC7092819

[160] Katulanda P , Dissanayake HA , Ranathunga I , et al. Prevention and management of COVID‐19 among patients with diabetes: an appraisal of the literature. Diabetologia. 2020;63(8):1440‐1452. 32405783 10.1007/s00125-020-05164-x PMC7220850

[161] Anguiano L , Riera M , Pascual J , Soler MJ . Circulating ACE2 in cardiovascular and kidney diseases. Curr Med Chem. 2017;24(30):3231‐3241. 28413960 10.2174/0929867324666170414162841

[162] Peric S , Stulnig TM . Diabetes and COVID‐19: disease‐management‐people. Wien Klin Wochenschr. 2020;132(13‐14):356‐361. 32435867 10.1007/s00508-020-01672-3 PMC7238399

[163] Yang JK , Lin SS , Ji XJ , Guo LM . Binding of SARS coronavirus to its receptor damages islets and causes acute diabetes. Acta Diabetol. 2010;47(3):193‐199. 19333547 10.1007/s00592-009-0109-4 PMC7088164

[164] Behzad S , Aghaghazvini L , Radmard AR , Gholamrezanezhad A . Extrapulmonary manifestations of COVID‐19: radiologic and clinical overview. Clin Imaging. 2020;66:35‐41. 32425338 10.1016/j.clinimag.2020.05.013 PMC7233216

[165] Jin M , Tong Q . Rhabdomyolysis as potential late complication associated with COVID‐19. Emerg Infect Dis. 2020;26(7):1618‐1620. 32197060 10.3201/eid2607.200445 PMC7323559

[166] Zaim S , Chong JH , Sankaranarayanan V , Harky A . COVID‐19 and multiorgan response. Curr Probl Cardiol. 2020;45(8):100618. 32439197 10.1016/j.cpcardiol.2020.100618 PMC7187881

[167] Xu X , Yu C , Qu J , et al. Imaging and clinical features of patients with 2019 novel coronavirus SARS‐CoV‐2. Eur J Nucl Med Mol Imaging. 2020;47(5):1275‐1280. 32107577 10.1007/s00259-020-04735-9 PMC7080117

[168] Stewart DJ , Hartley JC , Johnson M , Marks SD , du Pré P , Stojanovic J . Renal dysfunction in hospitalised children with COVID‐19. Lancet Child Adolesc Health. 2020;4(8):e28‐e29. 32553126 10.1016/S2352-4642(20)30178-4 PMC7295466

[169] Zhou B , She J , Wang Y , Ma X . The clinical characteristics of myocardial injury in severe and very severe patients with 2019 novel coronavirus disease. J Infect. 2020;81(1):147‐178. 10.1016/j.jinf.2020.03.021 PMC7163185 32209382

[170] Bansal M . Cardiovascular disease and COVID‐19. Diabetes Metab Syndr. 2020;14(3):247‐250. 32247212 10.1016/j.dsx.2020.03.013 PMC7102662

[171] Albarello F , Pianura E , Di Stefano F , et al. 2019‐novel coronavirus severe adult respiratory distress syndrome in two cases in Italy: an uncommon radiological presentation. Int J Infect Dis. 2020;93:192‐197. 32112966 10.1016/j.ijid.2020.02.043 PMC7110436

[172] Valette X , du Cheyron D , Goursaud S . Mediastinal lymphadenopathy in patients with severe COVID‐19. Lancet Infect Dis. 2020;20(11):1230. 10.1016/S1473-3099(20)30310-8 PMC7173806 32330440

[173] Taweesedt PT , Surani S . Mediastinal lymphadenopathy in COVID‐19: a review of literature. World J Clin Cases. 2021;9(12):2703‐2710. 33969053 10.12998/wjcc.v9.i12.2703 PMC8058669

[174] Desforges M , Le Coupanec A , Dubeau P , et al. Human coronaviruses and other respiratory viruses: underestimated opportunistic pathogens of the central nervous system? Viruses. 2019;12(1):14. 10.3390/v12010014 PMC7020001 31861926

[175] Fodoulian L , Tuberosa J , Rossier D , et al. SARS‐CoV‐2 receptors and entry genes are expressed in the human olfactory neuroepithelium and brain. iScience. 2020;23(12):101839. 33251489 10.1016/j.isci.2020.101839 PMC7685946

[176] Baig AM , Khaleeq A , Ali U , Syeda H . Evidence of the COVID‐19 virus targeting the CNS: tissue distribution, host–virus interaction, and proposed neurotropic mechanisms. ACS Chem Neurosci. 2020;11(7):995‐998. 32167747 10.1021/acschemneuro.0c00122

[177] Needham EJ , Chou SH , Coles AJ , Menon DK . Neurological implications of COVID‐19 infections. Neurocrit Care. 2020;32(3):667‐671. 32346843 10.1007/s12028-020-00978-4 PMC7188454

[178] Song YG , Shin HS . COVID‐19, a clinical syndrome manifesting as hypersensitivity pneumonitis. Infect Chemother. 2020;52(1):110‐112. 32153144 10.3947/ic.2020.52.1.110 PMC7113449

[179] Rothan HA , Byrareddy SN . The epidemiology and pathogenesis of coronavirus disease (COVID‐19) outbreak. J Autoimmun. 2020;109:102433. 32113704 10.1016/j.jaut.2020.102433 PMC7127067

[180] Liu K , Fang YY , Deng Y , et al. Clinical characteristics of novel coronavirus cases in tertiary hospitals in Hubei Province. Chin Med J (Engl). 2020;133(9):1025‐1031. 32044814 10.1097/CM9.0000000000000744 PMC7147277

[181] Xiong S , Liu L , Lin F , et al. Clinical characteristics of 116 hospitalized patients with COVID‐19 in Wuhan, China: a single‐centered, retrospective, observational study. BMC Infect Dis. 2020;20(1):787. 33092539 10.1186/s12879-020-05452-2 PMC7578439

[182] Wang Y , Wang Y , Chen Y , Qin Q . Unique epidemiological and clinical features of the emerging 2019 novel coronavirus pneumonia (COVID‐19) implicate special control measures. J Med Virol. 2020;92(6):568‐576. 32134116 10.1002/jmv.25748 PMC7228347

[183] Frater JL , Zini G , d' Onofrio G , Rogers HJ . COVID‐19 and the clinical hematology laboratory. Int J Lab Hematol. 2020;42(Suppl 1):11‐18. 32311826 10.1111/ijlh.13229 PMC7264622

[184] Wang S , Guo L , Chen L , et al. A case report of neonatal 2019 coronavirus disease in China. Clin Infect Dis. 2020;71(15):853‐857. 32161941 10.1093/cid/ciaa225 PMC7108144

[185] Dong L , Tian J , He S , et al. Possible vertical transmission of SARS‐CoV‐2 from an infected mother to her newborn. JAMA. 2020;323(18):1846‐1848. 32215581 10.1001/jama.2020.4621 PMC7099527

[186] Wong SF , Chow KM , Leung TN , et al. Pregnancy and perinatal outcomes of women with severe acute respiratory syndrome. Am J Obstet Gynecol. 2004;191(1):292‐297. 15295381 10.1016/j.ajog.2003.11.019 PMC7137614

[187] Phoswa WN , Khaliq OP . Is pregnancy a risk factor of COVID‐19? Eur J Obstet Gynecol. 2020;252:605‐609. 10.1016/j.ejogrb.2020.06.058 PMC7320674 32620513

[188] Wastnedge EA , Reynolds RM , van Boeckel SR , et al. Pregnancy and COVID‐19. Physiol Rev. 2021;101:303‐318. 32969772 10.1152/physrev.00024.2020 PMC7686875

[189] Schwartz DA . An analysis of 38 pregnant women with COVID‐19, their newborn infants, and maternal–fetal transmission of SARS‐CoV‐2: maternal coronavirus infections and pregnancy outcomes. Arch Pathol Lab Med. 2020;144(7):799‐805. 32180426 10.5858/arpa.2020-0901-SA

[190] Teng J , Dai J , Su Y , et al. Detection of IgM and IgG antibodies against SARS‐CoV‐2 in patients with autoimmune diseases. Lancet Rheumatol. 2020;2(7):e384‐e385. 32835238 10.1016/S2665-9913(20)30128-4 PMC7234786

[191] Liu Y , Sawalha AH , Lu Q . COVID‐19 and autoimmune diseases. Curr Opin Rheumatol. 2021;33(2):155‐162. 33332890 10.1097/BOR.0000000000000776 PMC7880581

[192] Stroppa EM , Toscani I , Citterio C , et al. Coronavirus disease‐2019 in cancer patients. A report of the first 25 cancer patients in a western country (Italy). Future Oncol. 2020;16(20):1425‐1432. 32403946 10.2217/fon-2020-0369 PMC7222528

[193] Liang W , Guan W , Chen R , et al. Cancer patients in SARS‐CoV‐2 infection: a nationwide analysis in China. Lancet Oncol. 2020;21(3):335‐337. 32066541 10.1016/S1470-2045(20)30096-6 PMC7159000

[194] Liu H , Yang D , Chen X , et al. The effect of anticancer treatment on cancer patients with COVID‐19: a systematic review and meta‐analysis. Cancer Med. 2020;10(3):1043‐1056. 33381923 10.1002/cam4.3692 PMC7897967

[195] Poortmans PM , Guarneri V , Cardoso MJ . Cancer and COVID‐19: what do we really know? Lancet. 2020;395(10241):1884‐1885. 32479827 10.1016/S0140-6736(20)31240-X PMC7259910

[196] Drożdżal S , Rosik J , Lechowicz K , et al. FDA approved drugs with pharmacotherapeutic potential for SARS‐CoV‐2 (COVID‐19) therapy. Drug Resist Updat. 2020;53:100719. 32717568 10.1016/j.drup.2020.100719 PMC7362818

[197] Al‐Tawfiq JA , Al‐Homoud AH , Memish ZA . Remdesivir as a possible therapeutic option for the COVID‐19. Travel Med Infect Dis. 2020;34:101615. 32145386 10.1016/j.tmaid.2020.101615 PMC7129391

[198] Huang F , Li Y , Leung EL , et al. A review of therapeutic agents and Chinese herbal medicines against SARS‐COV‐2 (COVID‐19). Pharmacol Res. 2020;158:104929. 32442720 10.1016/j.phrs.2020.104929 PMC7237953

[199] Sanders JM , Monogue ML , Jodlowski TZ , Cutrell JB . Pharmacologic treatments for coronavirus disease 2019 (COVID‐19): a review. JAMA. 2020;323(18):1824‐1836. 32282022 10.1001/jama.2020.6019

[200] Di Lorenzo G , Di Trolio R , Kozlakidis Z , et al. COVID 19 therapies and anti‐cancer drugs: a systematic review of recent literature. Crit Rev Oncol Hematol. 2020;152:102991. 32544802 10.1016/j.critrevonc.2020.102991 PMC7239789

[201] Parang K , El‐Sayed NS , Kazeminy AJ , Tiwari RK . Comparative antiviral activity of remdesivir and anti‐HIV nucleoside analogs against human coronavirus 229E (HCoV‐229E). Molecules. 2020;25(10):2343. 10.3390/molecules25102343 PMC7287735 32429580

[202] Babadaei MMN , Hasan A , Vahdani Y , et al. Development of remdesivir repositioning as a nucleotide analog against COVID‐19 RNA dependent RNA polymerase. J Biomol Struct Dyn. 2021;39(10):3771‐3779. 32397906 10.1080/07391102.2020.1767210 PMC7256352

[203] Yan VC , Muller FL . Advantages of the parent nucleoside GS‐441524 over remdesivir for covid‐19 treatment. ACS Med Chem Lett. 2020;11(7):1361‐1366. 32665809 10.1021/acsmedchemlett.0c00316 PMC7315846

[204] Amirian ES , Levy JK . Current knowledge about the antivirals remdesivir (GS‐5734) and GS‐441524 as therapeutic options for coronaviruses. One Health. 2020;9:100128. 32258351 10.1016/j.onehlt.2020.100128 PMC7118644

[205] Choy KT , Wong AY , Kaewpreedee P , et al. Remdesivir, lopinavir, emetine, and homoharringtonine inhibit SARS‐CoV‐2 replication in vitro. Antiviral Res. 2020;178:104786. 32251767 10.1016/j.antiviral.2020.104786 PMC7127386

[206] Wang M , Cao R , Zhang L , et al. Remdesivir and chloroquine effectively inhibit the recently emerged novel coronavirus (2019‐nCoV) in vitro. Cell Res. 2020;30(3):269‐271. 32020029 10.1038/s41422-020-0282-0 PMC7054408

[207] Badgujar KC , Badgujar AB , Patil VP , Dhangar DV . Hydroxychloroquine for COVID‐19: a review and a debate based on available clinical trials/case studies. J Drug Deliv Ther. 2020;10(3):304‐311.

[208] Touret F , de Lamballerie X . Of chloroquine and COVID‐19. Antiviral Res. 2020;177:104762. 32147496 10.1016/j.antiviral.2020.104762 PMC7132364

[209] Principi N , Esposito S . Chloroquine or hydroxychloroquine for prophylaxis of COVID‐19. Lancet Infect Dis. 2020;20(10):1118. 10.1016/S1473-3099(20)30296-6 PMC7164862 32311322

[210] Ferner RE , Aronson JK . Chloroquine and hydroxychloroquine in covid‐19. BMJ. 2020;369:m1432. 32269046 10.1136/bmj.m1432

[211] Quiros Roldan E , Biasiotto G , Magro P , Zanella I . The possible mechanisms of action of 4‐aminoquinolines (chloroquine/hydroxychloroquine) against Sars‐Cov‐2 infection (COVID‐19): a role for iron homeostasis? Pharmacol Res. 2020;158:104904. 32430286 10.1016/j.phrs.2020.104904 PMC7217799

[212] Devaux CA , Rolain JM , Colson P , Raoult D . New insights on the antiviral effects of chloroquine against coronavirus: what to expect for COVID‐19? Int J Antimicrob Agents. 2020;55(5):105938. 32171740 10.1016/j.ijantimicag.2020.105938 PMC7118659

[213] Mehra MR , Desai SS , Ruschitzka F , Patel AN . Retracted: hydroxychloroquine or chloroquine with or without a macrolide for treatment of COVID‐19: a multinational registry analysis. Lancet. 2020;396(10245):e2‐e3. 32450107 10.1016/S0140-6736(20)31180-6 PMC7255293

[214] Roustit M , Guilhaumou R , Molimard M , et al. Chloroquine and hydroxychloroquine in the management of COVID‐19: much kerfuffle but little evidence. Therapie. 2020;75(4):363‐370. 32473812 10.1016/j.therap.2020.05.010 PMC7244425

[215] Zimmer C, Corum J, Wee SL, Kristoffersen M. The New York Times. Coronavirus Vaccine Tracker. https://www.nytimes.com/interactive/2020/science/coronavirus-vaccine-tracker.html. Accessed November 29, 2021.

[216] Choudhary R , Sharma AK . Potential use of hydroxychloroquine, ivermectin and azithromycin drugs in fighting COVID‐19: trends, scope and relevance. New Microbes New Infect. 2020;35:100684. 32322397 10.1016/j.nmni.2020.100684 PMC7175902

[217] Andreani J , Le Bideau M , Duflot I , et al. *In vitro* testing of combined hydroxychloroquine and azithromycin on SARS‐CoV‐2 shows synergistic effect. Microb Pathog. 2020;145:104228. 32344177 10.1016/j.micpath.2020.104228 PMC7182748

[218] Gautret P , Lagier JC , Parola P , et al. Hydroxychloroquine and azithromycin as a treatment of COVID‐19: results of an open‐label non‐randomized clinical trial. Int J Antimicrob Agents. 2020;56(1):105949. 32205204 10.1016/j.ijantimicag.2020.105949 PMC7102549

[219] Mahévas M , Tran VT , Roumier M , et al. Clinical efficacy of hydroxychloroquine in patients with covid‐19 pneumonia who require oxygen: observational comparative study using routine care data. BMJ. 2020;369(1):m1844. 32409486 10.1136/bmj.m1844 PMC7221472

[220] Xia T , Wang Y . Coronavirus disease 2019 and transplantation: the combination of lopinavir/ritonavir and hydroxychloroquine is responsible for excessive tacrolimus trough level and unfavorable outcome. Am J Transplant. 2020;20(9):2630‐2631. 32400965 10.1111/ajt.15992 PMC7273014

[221] Romão VC , Cruz‐Machado AR , Fonseca JE . No evidence so far on the protective effect of hydroxychloroquine to prevent COVID‐19: comment by Joob and Wiwanitkit. Ann Rheum Dis. 2021;80(2):e22. 32404340 10.1136/annrheumdis-2020-217665

[222] Mazzitelli M , Davoli C , Scaglione V , et al. Apparent inefficacy of hydroxychloroquine combined with azithromycin on SARS‐CoV‐2 clearance in an incident cohort of geriatric patients with COVID‐19. Travel Med Infect Dis. 2020;37:101826. 32739472 10.1016/j.tmaid.2020.101826 PMC7392844

[223] Sharma S , Ray A , Sadasivam B . Metformin in COVID‐19: a possible role beyond diabetes. Diabetes Res Clin Pract. 2020;164:108183. 32360697 10.1016/j.diabres.2020.108183 PMC7190487

[224] Luo P , Qiu L , Liu Y , et al. Metformin treatment was associated with decreased mortality in COVID‐19 patients with diabetes in a retrospective analysis. Am J Trop Med Hyg. 2020;103(1):69‐72. 32446312 10.4269/ajtmh.20-0375 PMC7356425

[225] Gong WJ , Zhou T , Wu SL , et al. A retrospective analysis of clinical efficacy of ribavirin in adults hospitalized with severe COVID‐19. J Infect Chemother. 2021;27(6):876‐881. 33676844 10.1016/j.jiac.2021.02.018 PMC7894089

[226] Xu J , Shi PY , Li H , Zhou J . Broad spectrum antiviral agent niclosamide and its therapeutic potential. ACS Infect Dis. 2020;6(5):909‐915. 32125140 10.1021/acsinfecdis.0c00052 PMC7098069

[227] Jeon S , Ko M , Lee J , et al. Identification of antiviral drug candidates against SARS‐CoV‐2 from FDA‐approved drugs. Antimicrob Agents Chemother. 2020;64(7):e00819‐20. 32366720 10.1128/AAC.00819-20 PMC7318052

[228] Caly L , Druce JD , Catton MG , Jans DA , Wagstaff KM . The FDA‐approved drug ivermectin inhibits the replication of SARS‐CoV‐2 in vitro. Antiviral Res. 2020;178:104787. 32251768 10.1016/j.antiviral.2020.104787 PMC7129059

[229] Sharun K , Dhama K , Patel SK , et al. Ivermectin, a new candidate therapeutic against SARS‐CoV‐2/COVID‐19. Ann Clin Microbiol Antimicrob. 2020;19(1):23. 32473642 10.1186/s12941-020-00368-w PMC7261036

[230] Cure E , Kucuk A , Cumhur Cure M . Cyclosporine therapy in cytokine storm due to coronavirus disease 2019 (COVID‐19). Rheumatol Int. 2020;40(7):1177‐1179. 32415310 10.1007/s00296-020-04603-7 PMC7227450

[231] Ma‐Lauer Y , Zheng Y , Malešević M , von Brunn B , Fischer G , von Brunn A . Interaction of cyclophilin A and human coronavirus 229E N protein is essential for virus replication. Antiviral Res. 2020;173:104620. 31634494 10.1016/j.antiviral.2019.104620 PMC7114175

[232] de Wilde AH , Falzarano D , Zevenhoven‐Dobbe JC , et al. Alisporivir inhibits MERS‐ and SARS‐coronavirus replication in cell culture, but not SARS‐coronavirus infection in a mouse model. Virus Res. 2017;228:7‐13. 27840112 10.1016/j.virusres.2016.11.011 PMC7114565

[233] Pawlotsky JM . COVID‐19 pandemic: time to revive the cyclophilin inhibitor alisporivir. Clin Infect Dis. 2020;71(16):2191‐2194. 32409832 10.1093/cid/ciaa587 PMC7239253

[234] Buonaguro L , Buonaguro FM . Knowledge‐based repositioning of the anti‐HCV direct antiviral agent Sofosbuvir as SARS‐CoV‐2 treatment. Infect Agents Cancer. 2020;15:32. 10.1186/s13027-020-00302-x PMC7215134 32419838

[235] Sayad B , Sobhani M , Khodarahmi R . Sofosbuvir as repurposed antiviral drug against COVID‐19: why were we convinced to evaluate the drug in a registered/approved clinical trial? Arch Med Res. 2020;51(6):577‐581. 32387040 10.1016/j.arcmed.2020.04.018 PMC7188631

[236] Yao TT , Qian JD , Zhu WY , Wang Y , Wang GQ . A systematic review of lopinavir therapy for SARS coronavirus and MERS coronavirus—a possible reference for coronavirus disease‐19 treatment option. J Med Virol. 2020;92(6):556‐563. 32104907 10.1002/jmv.25729 PMC7217143

[237] Cattaneo D , Cattaneo D , Gervasoni C , et al. Does lopinavir really inhibit SARS‐CoV‐2? Pharmacol Res. 2020;158:104898. 32438034 10.1016/j.phrs.2020.104898 PMC7211645

[238] Tobaiqy M , Qashqary M , Al‐Dahery S , et al. Therapeutic management of patients with COVID‐19: a systematic review. Infect Prev Pract. 2020;2(3):100061. 34316558 10.1016/j.infpip.2020.100061 PMC7162768

[239] Cao B , Wang Y , Wen D , et al. A trial of lopinavir‐ritonavir in adults hospitalized with severe Covid‐19. N Engl J Med. 2020;382(19):1787‐1799. 32187464 10.1056/NEJMoa2001282 PMC7121492

[240] Yamamoto N , Yang R , Yoshinaka Y , et al. HIV protease inhibitor nelfinavir inhibits replication of SARS‐associated coronavirus. Biochem Biophys Res Commun. 2004;318(3):719‐725. 15144898 10.1016/j.bbrc.2004.04.083 PMC7111005

[241] Musarrat F , Chouljenko V , Dahal A , et al. The anti‐HIV drug nelfinavir mesylate (Viracept) is a potent inhibitor of cell fusion caused by the SARSCoV‐2 spike (S) glycoprotein warranting further evaluation as an antiviral against COVID‐19 infections. J Med Virol. 2020;92(10):2087‐2095. 32374457 10.1002/jmv.25985 PMC7267418

[242] Xu Z, Yao H, Shen J, et al. Nelfinavir is active against SARS‐CoV‐2 in vero E6 cells. ChemRxiv. Published online March 30, 2020. 10.26434/chemrxiv.12039888.v1

[243] Yanai H . Favipiravir: a possible pharmaceutical treatment for COVID‐19. J Endocrinol Metab. 2020;10(2):33‐34.

[244] Shannon A, Selisko B, Le N, et al. Favipiravir strikes the SARS‐CoV‐2 at its Achilles heel, the RNA polymerase. bioRxiv. Published online May 15, 2020. 10.1101/2020.05.15.098731

[245] Coomes EA , Haghbayan H . Favipiravir, an antiviral for COVID‐19? J Antimicrob Chemother. 2020;75(7):2013‐2014. 32417899 10.1093/jac/dkaa171 PMC7239147

[246] Cai Q , Yang M , Liu D , et al. Experimental treatment with favipiravir for COVID‐19: an open‐label control study. Engineering (Beijing). 2020;6(10):1192‐1198. 32346491 10.1016/j.eng.2020.03.007 PMC7185795

[247] Joshi S , Parkar J , Ansari A , et al. Role of favipiravir in the treatment of COVID‐19. Int J Infect Dis. 2021;102:501‐508. 33130203 10.1016/j.ijid.2020.10.069 PMC7831863

[248] Wu R , Wang L , Kuo HD , et al. An update on current therapeutic drugs treating COVID‐19. Curr Pharmacol Rep. 2020;6(3):56‐70. 32395418 10.1007/s40495-020-00216-7 PMC7211915

[249] Vankadari N . Arbidol: a potential antiviral drug for the treatment of SARS‐CoV‐2 by blocking trimerization of the spike glycoprotein. Int J Antimicrob Agents. 2020;56(2):105998. 32360231 10.1016/j.ijantimicag.2020.105998 PMC7187825

[250] Zhu Z , Lu Z , Xu T , et al. Arbidol monotherapy is superior to lopinavir/ritonavir in treating COVID‐19. J Infect. 2020;81(1):e21‐e23. 32283143 10.1016/j.jinf.2020.03.060 PMC7195393

[251] Meo SA , Zaidi SZA , Shang T , et al. Biological, molecular and pharmacological characteristics of chloroquine, hydroxychloroquine, convalescent plasma, and remdesivir for COVID‐19 pandemic: a comparative analysis. J King Saud Univ Sci. 2020;32(7):3159‐3166. 32921965 10.1016/j.jksus.2020.09.002 PMC7474813

[252] RECOVERY Collaborative Group . Azithromycin in patients admitted to hospital with COVID‐19 (recovery): a randomised, controlled, open‐label, platform trial. Lancet. 2021;397(10274):605‐612. 33545096 10.1016/S0140-6736(21)00149-5 PMC7884931

[253] Raha S , Mallick R , Basak S , Duttaroy AK . Is copper beneficial for COVID‐19 patients? Med Hypotheses. 2020;142:109814. 32388476 10.1016/j.mehy.2020.109814 PMC7199671

[254] Martel J , Ko YF , Young JD , Ojcius DM . Could nasal nitric oxide help to mitigate the severity of COVID‐19? Microbes Infect. 2020;22(4‐5):168‐171. 32387333 10.1016/j.micinf.2020.05.002 PMC7200356

[255] Wen J , Wang R , Liu H , et al. Potential therapeutic effect of Qingwen Baidu Decoction against Corona Virus Disease 2019: a mini review. Chin Med. 2020;15:48. 32454888 10.1186/s13020-020-00332-y PMC7235554

[256] Gigante A , Aquili A , Farinelli L , et al. Sodium chromo‐glycate and palmitoylethanolamide: a possible strategy to treat mast cell‐induced lung inflammation in COVID‐19. Med Hypotheses. 2020;143:109856. 32460208 10.1016/j.mehy.2020.109856 PMC7236677

[257] Salaris C , Scarpa M , Elli M , et al. Protective effects of lactoferrin against SARS‐CoV‐2 infection *in vitro* . Nutrients. 2021;13(2):328. 33498631 10.3390/nu13020328 PMC7911668

[258] Zhang L , Lin D , Kusov Y , et al. Alpha‐ketoamides as broad‐spectrum inhibitors of coronavirus and enterovirus replication structure‐based design, synthesis, and activity assessment. J Med Chem. 2020;63(9):4562–4578. 32045235 10.1021/acs.jmedchem.9b01828

[259] Zheng Y , Li R , Liu S . Immunoregulation with mTOR inhibitors to prevent COVID‐19 severity: a novel intervention strategy beyond vaccines and specific antiviral medicines. J Med Virol. 2020;92(9):1495‐1500. 32410266 10.1002/jmv.26009 PMC7272823

[260] Apaydın ÇB , Cesur N , Stevaert A , Naesens L , Cesur Z . Synthesis and anti‐coronavirus activity of a series of 1‐thia‐4‐azaspiro[4.5]decan‐3‐one derivatives. Arch Pharm Chem Life Sci. 2019;352(6):1800330. 10.1002/ardp.201800330 PMC7161747 31073993

[261] Evgen'ev MB , Frenkel A . Possible application of H2S‐producing compounds in therapy of coronavirus (COVID‐19) infection and pneumonia. Cell Stress Chaperones. 2020;25(5):713‐715. 32409956 10.1007/s12192-020-01120-1 PMC7221330

[262] Zhang YN , Zhang QY , Li XD , et al. Gemcitabine, lycorine and oxysophoridine inhibit novel coronavirus (SARS‐CoV‐2) in cell culture. Emerg Microbes Infect. 2020;9(1):1170‐1173. 32432977 10.1080/22221751.2020.1772676 PMC7448857

[263] Farag A, Wang P, Boys IN, et al. Identification of atovaquone, ouabain and mebendazole as FDA approved drugs targeting SARS‐CoV‐2 (version 4). ChemRxiv. Published online May 14, 2020. 10.26434/chemrxiv.12003930.v4

[264] Thibaud S , Tremblay D , Bhalla S , Zimmerman B , Sigel K , Gabrilove J . Protective role of Bruton tyrosine kinase inhibitors in patients with chronic lymphocytic leukaemia and COVID‐19. Br J Haematol. 2020;190(2):e73‐e76. 32433778 10.1111/bjh.16863 PMC7276870

[265] Rothan HA , Stone S , Natekar J , Kumari P , Arora K , Kumar M . The FDA‐approved gold drug auranofin inhibits novel coronavirus (SARS‐COV‐2) replication and attenuates inflammation in human cells. Virology. 2020;547:7‐11. 32442105 10.1016/j.virol.2020.05.002 PMC7236683

[266] Müller C , Schulte FW , Lange‐Grünweller K , et al. Broad‐spectrum antiviral activity of the eIF4A inhibitor silvestrol against corona‐ and picornaviruses. Antiviral Res. 2018;150:123‐129. 29258862 10.1016/j.antiviral.2017.12.010 PMC7113723

[267] Ma Q , Pan W , Li R , et al. Liu Shen capsule shows antiviral and anti‐inflammatory abilities against novel coronavirus SARS‐CoV‐2 via suppression of NF‐κB signaling pathway. Pharmacol Res. 2020;158:104850. 32360580 10.1016/j.phrs.2020.104850 PMC7192119

[268] Wang Y , Jiang W , He Q , et al. A retrospective cohort study of methylprednisolone therapy in severe patients with COVID‐19 pneumonia. Signal Transduct Target Ther. 2020;5(1):57. 32341331 10.1038/s41392-020-0158-2 PMC7186116

[269] Ekins S , Madrid PB . Tilorone, a broad‐spectrum antiviral for emerging viruses. Antimicrob Agents Chemother. 2020;64(5):e00440‐20. 32205350 10.1128/AAC.00440-20 PMC7179581

[270] Milewska A , Chi Y , Szczepanski A , et al. HTCC as a polymeric inhibitor of SARS‐CoV‐2 and MERS‐CoV. J Virol. 2021;95(4):e01622‐20. 33219167 10.1128/JVI.01622-20 PMC7851557

[271] Marinella MA . Indomethacin and resveratrol as potential treatment adjuncts for SARS‐CoV‐2/COVID‐19. Int J Clin Pract. 2020;74(9):e13535. 32412158 10.1111/ijcp.13535 PMC7261995

[272] Terrier O, Dilly S, Pizzorno A, et al. Broad‐spectrum antiviral activity of naproxen: from Influenza A to SARS‐CoV‐2 Coronavirus. bioRxiv. Published online May 1, 2020. 10.1101/2020.04.30.069922

[273] Fiorino S , Gallo C , Zippi M , et al. Cytokine storm in aged people with CoV‐2: possible role of vitamins as therapy or preventive strategy. Aging Clin Exp Res. 2020;32(10):2115‐2131. 32865757 10.1007/s40520-020-01669-y PMC7456763

[274] Ali N . Role of vitamin D in preventing of COVID‐19 infection, progression and severity. J Infect Public Health. 2020;13:1373‐138. 32605780 10.1016/j.jiph.2020.06.021 PMC7305922

[275] Whittemore PB . COVID‐19 fatalities, latitude, sunlight, and vitamin D. Am J Infect Control. 2020;48(9):1042‐1044. 32599103 10.1016/j.ajic.2020.06.193 PMC7319635

[276] Rajendran K , Krishnasamy N , Rangarajan J , Rathinam J , Natarajan M , Ramachandran A . Convalescent plasma transfusion for the treatment of COVID‐19: systematic review. J Med Virol. 2020;92(9):1475‐1483. 32356910 10.1002/jmv.25961 PMC7267113

[277] Charaa N, Chahed M, Ghedira H, Daghfous R. Prophylactic Treatment Protocol against the Severity of COVID‐19 Using Melatonin. SSRN. Published online May 19, 2020. https://ssrn.com/abstract=3601861

[278] Wang C , Li W , Drabek D , et al. A human monoclonal antibody blocking SARS‐CoV‐2 infection. Nat Commun. 2020;11(1):2251. 32366817 10.1038/s41467-020-16256-y PMC7198537

[279] F Hoffmann‐La Roche Ltd & Media & investor release . Japan Becomes First Country to Approve Ronapreve (Casirivimab and Imdevimab) for the Treatment of Mild to Moderate COVID‐19. https://www.roche.com/media/releases/med-cor-2021-07-20.htm. Accessed July 20, 2021.

[280] Saha A , Sharma AR , Bhattacharya M , Sharma G , Lee SS , Chakraborty C . Tocilizumab: a therapeutic option for the treatment of cytokine storm syndrome in COVID‐19. Arch Med Res. 2020;51(6):595‐597. 32482373 10.1016/j.arcmed.2020.05.009 PMC7241374

[281] Magro G . SARS‐CoV‐2 and COVID‐19: is interleukin‐6 (IL‐6) the ‘culprit lesion’ of ARDS onset? What is there besides Tocilizumab? SGP130Fc. Cytokine X. 2020;2(2):100029. 32421092 10.1016/j.cytox.2020.100029 PMC7224649

[282] Rimland CA , Morgan CE & Bell GJ et al. Clinical characteristics and early outcomes in patients with COVID‐19 treated with tocilizumab at a United States academic center. medRxiv. 2020. 10.1101/2020.05.13.20100404

[283] Sánchez‐Montalvá A , Sellarés‐Nadal J , Espinosa‐Pereiro J , et al. Early outcomes in adults hospitalized with severe SARS‐CoV‐2 infection receiving tocilizumab. Med Clin (Barc). 2021;S0025‐7753(21)00360‐2. 10.1016/j.medcli.2021.06.012 PMC8448395 34544604

[284] Hung IFN , Lung KC , Tso EYK , et al. Triple combination of interferon beta‐1b, lopinavir–ritonavir, and ribavirin in the treatment of patients admitted to hospital with COVID‐19: an open‐label, randomised, phase 2 trial. Lancet. 2020;395(10238):1695‐1704. 32401715 10.1016/S0140-6736(20)31042-4 PMC7211500

[285] Su S , Jiang S . A suspicious role of interferon in the pathogenesis of SARS‐CoV‐2 by enhancing expression of ACE2. Signal Transduct Target Ther. 2020;5(1):71. 32435059 10.1038/s41392-020-0185-z PMC7239689

[286] Monk PD , Marsden RJ , Tear VJ , et al. Safety and efficacy of inhaled nebulised interferon beta‐1a (SNG001) for treatment of SARS‐CoV‐2 infection: a randomised, double‐blind, placebo‐controlled, phase 2 trial. Lancet Respir Med. 2021;9(2):196‐206. 33189161 10.1016/S2213-2600(20)30511-7 PMC7836724

[287] Griffin S . Covid‐19: lopinavir‐ritonavir does not benefit hospitalised patients, UK trial finds. BMJ. 2020;370:m2650. 32611587 10.1136/bmj.m2650

[288] Xu X , Han M , Li T , et al. Effective treatment of severe COVID‐19 patients with tocilizumab. Proc Natl Acad Sci U S A. 2020;117(20):10970‐10975. 32350134 10.1073/pnas.2005615117 PMC7245089

[289] Liebich S , BabuBio LLC . Potent SELEX Aptamer‐Based Therapeutic Method for Novel SARS‐CoV2 Virus Disease (COVID‐19). COVID‐19 Treatment Proposal. 2020.

[290] Nascimento Junior JAC , Santos AM , Quintans‐Júnior LJ , Walker CIB , Borges LP , Serafini MR . SARS, MERS and SARS‐CoV‐2 (COVID‐19) treatment: a patent review. Expert Opin Ther Pat. 2020;30(8):567‐579. 32429703 10.1080/13543776.2020.1772231

[291] Luo P , Liu D , Li J . Pharmacological perspective: glycyrrhizin may be an efficacious therapeutic agent for COVID‐19. Int J Antimicrob Agents. 2020;55(6):105995. 32335281 10.1016/j.ijantimicag.2020.105995 PMC7180159

[292] Murck H . Symptomatic protective action of glycyrrhizin (licorice) in COVID‐19 infection? Front Immunol. 2020;11:1239. 32574273 10.3389/fimmu.2020.01239 PMC7270278

[293] Golchin A , Seyedjafari E , Ardeshirylajimi A . Mesenchymal stem cell therapy for COVID‐19: present or future. Stem Cell Rev Rep. 2020;16(3):427‐433. 32281052 10.1007/s12015-020-09973-w PMC7152513

[294] Ghosh S , Firdous SM , Nath A . siRNA could be a potential therapy for COVID‐19. EXCLI J. 2020;19:528‐531. 32398976 10.17179/excli2020-1328 PMC7214778

[295] Miller A , Reandelar MJ , Fasciglione K , Roumenova V , Li Y, Otazu GH. Correlation between universal BCG vaccination policy and reduced morbidity and mortality for COVID‐19: an epidemiological study. medRxiv. Published online March 28, 2020. 10.1101/2020.03.24.20042937

[296] van Riel D , de Wit E . Next‐generation vaccine platforms for COVID‐19. Nat Mater. 2020;19(8):810‐812. 32704139 10.1038/s41563-020-0746-0

[297] Liu C , Zhou Q , Li Y , et al. Research and development on therapeutic agents and vaccines for COVID‐19 and related human coronavirus diseases. ACS Cent Sci. 2020;6(3):315‐331. 32226821 10.1021/acscentsci.0c00272 PMC10467574

[298] Abdoli A , Aalizadeh R , Aminianfar H , et al. Safety and potency of BIV1‐CovIran inactivated vaccine candidate for SARS‐CoV‐2: a preclinical study. Rev Med Virol. 2021;e2305. 34699647 10.1002/rmv.2305 PMC8646699

[299] Zhang J , Zeng H , Gu J , Li H , Zheng L , Zou Q . Progress and prospects on vaccine development against SARS‐CoV‐2. Vaccines (Basel). 2020;8(2):153. 10.3390/vaccines8020153 PMC7349596 32235387

[300] El Sahly HM , Baden LR , Essink B , et al. Efficacy of the mRNA‐1273 SARS‐CoV‐2 vaccine at completion of blinded phase. N Engl J Med. 2021;385(19):1774‐1785. 34551225 10.1056/NEJMoa2113017 PMC8482810

[301] Uskoković V . Why have nanotechnologies been underutilized in the global uprising against the coronavirus pandemic? Nanomedicine (Lond). 2020;15(17):1719‐1734. 32462968 10.2217/nnm-2020-0163 PMC7265684

[302] Siddiquie RY , Agrawal A , Joshi SS . Surface alterations to impart antiviral properties to combat COVID‐19 transmission. Trans Indian Natl Acad Eng. 2020;1‐5. 10.1007/s41403-020-00096-9 PMC7223978 38624346

[303] van Doremalen N , Bushmaker T , Morris DH , et al. Aerosol and surface stability of SARS‐CoV‐2 as compared with SARS‐CoV‐1. N Engl J Med. 2020;382(16):1564‐1567. 32182409 10.1056/NEJMc2004973 PMC7121658

[304] Zhong H , Zhu Z , You P , et al. Plasmonic and superhydrophobic self‐decontaminating N95 respirators. ACS Nano. 2020;14(7):8846‐8854. 32578981 10.1021/acsnano.0c03504

[305] Balagna C , Perero S , Percivalle E , Nepita EV , Ferraris M . Virucidal effect against coronavirus SARS‐CoV‐2 of a silver nanocluster/silica composite sputtered coating. Open Ceramics. 2020;1:100006.

[306] El‐Atab N , Qaiser N , Badghaish H , Shaikh SF , Hussain MM . Flexible nanoporous template for the design and development of reusable anti‐COVID‐19 hydrophobic face masks. ACS Nano. 2020;14(6):7659‐7665. 32432461 10.1021/acsnano.0c03976 PMC7243426

[307] Joe YH , Park DH , Hwang J . Evaluation of Ag nanoparticle coated air filter against aerosolized virus: anti‐viral efficiency with dust loading. J Hazard Mater. 2016;301:547‐553. 26434534 10.1016/j.jhazmat.2015.09.017 PMC7116979

[308] Stanford MG , Li JT , Chen Y , et al. Self‐sterilizing laser‐induced graphene bacterial air filter. ACS Nano. 2019;13(10):11912‐11920. 31560513 10.1021/acsnano.9b05983

[309] Rai PK , Usmani Z , Thakur VK , Gupta VK , Mishra YK . Tackling COVID‐19 pandemic through nanocoatings: confront and exactitude. Curr Res Green Sustain Chem. 2020;3:100011.

[310] Vaze N , Pyrgiotakis G , McDevitt J , et al. Inactivation of common hospital acquired pathogens on surfaces and in air utilizing engineered water nanostructures (EWNS) based nano‐sanitizers. Nanomedicine. 2019;18:234‐242. 30904585 10.1016/j.nano.2019.03.003 PMC6588479

[311] Anti‐viral surface coating to prevent the spread of COVID‐19. Focus on Powder Coatings. 2020;2020(7):5.

[312] Vazquez‐Munoz R , Lopez‐Ribot JL . Nanotechnology as an alternative to reduce the spread of COVID‐19. Challenges. 2020;11(2):15.

[313] Weiss C , Carriere M , Fusco L , et al. Toward nanotechnology‐enabled approaches against the COVID‐19 pandemic. ACS Nano. 2020;14(6):6383‐6406. 32519842 10.1021/acsnano.0c03697

[314] Fernando SSN , Gunasekara TDCP , Holton J . Antimicrobial nanoparticles: applications and mechanisms of action. Sri Lankan J Infect Dis. 2018;8(1):2.

[315] Kaweeteerawat C , Na Ubol P , Sangmuang S , Aueviriyavit S , Maniratanachote R . Mechanisms of antibiotic resistance in bacteria mediated by silver nanoparticles. J Toxicol Environ Health A. 2017;80(23‐24):1276‐1289. 29020531 10.1080/15287394.2017.1376727

[316] Chatterjee AK , Chakraborty R , Basu T . Mechanism of antibacterial activity of copper nanoparticles. Nanotechnology. 2014;25(13):135101. 24584282 10.1088/0957-4484/25/13/135101

[317] Sirelkhatim A , Mahmud S , Seeni A , et al. Review on zinc oxide nanoparticles: antibacterial activity and toxicity mechanism. Nanomicro Lett. 2015;7(3):219‐242. 30464967 10.1007/s40820-015-0040-x PMC6223899

[318] Tallury P , Malhotra A , Byrne LM , Santra S . Nanobioimaging and sensing of infectious diseases. Adv Drug Deliv Rev. 2010;62(4‐5):424‐437. 19931579 10.1016/j.addr.2009.11.014 PMC7103339

[319] Malik P , Katyal V , Malik V , Asatkar A , Inwati G , Mukherjee TK . Nanobiosensors: concepts and variations. ISRN Nanomater. 2013;2013:1‐9.

[320] Noah NM , Ndangili PM . Current trends of nanobiosensors for point‐of‐care diagnostics. J Anal Methods Chem. 2019;2019:1‐16. 10.1155/2019/2179718 PMC6925704 31886019

[321] Saylan Y , Denizli A . Virus detection using nanosensors. Han B , Tomer VK , Nguyen TA , Farmani A , Singh PK (eds.). Nanosensors for Smart Cities. 2020;501‐511.

[322] Qiu G , Gai Z , Tao Y , Schmitt J , Kullak‐Ublick GA , Wang J . Dual‐functional plasmonic photothermal biosensors for highly accurate severe acute respiratory syndrome coronavirus 2 detection. ACS Nano. 2020;14(5):5268‐5277. 32281785 10.1021/acsnano.0c02439

[323] Maharia S , Robertsa A , Shahdeoa D, Gandhi S. eCovSens‐ultrasensitive novel in‐house built printed circuit board based electrochemical device for rapid detection of nCovid‐19 antigen, a spike protein domain 1 of SARS‐CoV‐2. bioRxiv. Published online March 28, 2020. 10.1101/2020.04.24.059204

[324] Seo G , Lee G , Kim MJ , et al. Rapid detection of COVID‐19 causative virus (SARS‐CoV‐2) in human nasopharyngeal swab specimens using field‐effect transistor‐based biosensor. ACS Nano. 2020;14(4):5135‐5142. 32293168 10.1021/acsnano.0c02823

[325] Zhu X , Wang X , Han L , et al. Multiplex reverse transcription loop‐mediated isothermal amplification combined with nanoparticle‐based lateral flow biosensor for the diagnosis of COVID‐19. Biosens Bioelectron. 2020;166:112437. 32692666 10.1016/j.bios.2020.112437 PMC7361114

[326] Chen Z , Zhang Z , Zhai X , et al. Rapid and sensitive detection of anti‐SARS‐CoV‐2 IgG, using lanthanide‐doped nanoparticles‐based lateral flow immunoassay. Anal Chem. 2020;92(10):7226‐7231. 32323974 10.1021/acs.analchem.0c00784

[327] Li Z , Yi Y , Luo X , et al. Development and clinical application of a rapid IgM‐IgG combined antibody test for SARS‐CoV‐2 infection diagnosis. J Med Virol. 2020;92(9):1518‐1524. 32104917 10.1002/jmv.25727 PMC7228300

[328] Lee Y , Kang BH , Kang M , et al. Nanoplasmonic on‐chip PCR for rapid precision molecular diagnostics. ACS Appl Mater Interfaces. 2020;12(11):12533‐12540. 32101396 10.1021/acsami.9b23591

[329] Layqah LA , Eissa S . An electrochemical immunosensor for the corona virus associated with the Middle East respiratory syndrome using an array of gold nanoparticle‐modified carbon electrodes. Mikrochim Acta. 2019;186(4):224. 30847572 10.1007/s00604-019-3345-5 PMC7088225

[330] Kim H , Park M , Hwang J , et al. Development of label‐free colorimetric assay for MERS‐CoV using gold nanoparticles. ACS Sens. 2019;4(5):1306‐1312. 31062580 10.1021/acssensors.9b00175

[331] Byers KM , Bird AR , Cho HD , Linnes JC . Fully dried two‐dimensional paper network for enzymatically enhanced detection of nucleic acid amplicons. ACS Omega. 2020;5(9):4673‐4681. 32175514 10.1021/acsomega.0c00115 PMC7066650

[332] Teengam P , Siangproh W , Tuantranont A , Vilaivan T , Chailapakul O , Henry CS . Multiplex paper‐based colorimetric DNA sensor using pyrrolidinyl peptide nucleic acid‐induced AgNPs aggregation for detecting MERS‐CoV, MTB, and HPV oligonucleotides. Anal Chem. 2017;89(10):5428‐5435. 28394582 10.1021/acs.analchem.7b00255 PMC7077925

[333] Ishikawa FN , Curreli M , Olson CA , et al. Importance of controlling nanotube density for highly sensitive and reliable biosensors functional in physiological conditions. ACS Nano. 2010;4(11):6914‐6922. 21028792 10.1021/nn101198u

[334] Ishikawa FN , Chang HK , Curreli M , et al. Label‐free, electrical detection of the SARS virus N‐protein with nanowire biosensors utilizing antibody mimics as capture probes. ACS Nano. 2009;3(5):1219‐1224. 19422193 10.1021/nn900086c PMC2765574

[335] Wu F , Mao M , Liu Q , et al. Ultra sensitive detection of influenza A virus based on Cdse/Zns quantum dots immunoassay. SOJ Biochem. 2016;2(3):2‐6.

[336] Krejcova L , Nejdl L , Rodrigo MA , et al. 3D printed chip for electrochemical detection of influenza virus labeled with CdS quantum dots. Biosens Bioelectron. 2014;54:421‐427. 24296063 10.1016/j.bios.2013.10.031

[337] Shah KG , Singh V , Kauffman PC , Abe K , Yager P . Mobile phone ratiometric imaging enables highly sensitive fluorescence lateral flow immunoassays without external optical filters. Anal Chem. 2018;90(11):6967‐6974. 29715012 10.1021/acs.analchem.8b01241

[338] Pang Y , Rong Z , Wang J , Xiao R , Wang S . A fluorescent aptasensor for H5N1 influenza virus detection based‐on the core‐shell nanoparticles metal‐enhanced fluorescence (MEF). Biosens Bioelectron. 2015;66:527‐532. 25506900 10.1016/j.bios.2014.10.052

[339] Sayhi M , Ouerghi O , Belgacem K , et al. Electrochemical detection of influenza virus H9N2 based on both immunomagnetic extraction and gold catalysis using an immobilization‐free screen printed carbon microelectrode. Biosens Bioelectron. 2018;107:170‐177. 29455027 10.1016/j.bios.2018.02.018

[340] Ahmed SR , Kang SW , Oh S , Lee J , Neethirajan S . Chiral zirconium quantum dots: a new class of nanocrystals for optical detection of coronavirus. Heliyon. 2018;4(8):e00766. 30186985 10.1016/j.heliyon.2018.e00766 PMC6120744

[341] Weng X , Neethirajan S . Immunosensor based on antibody‐functionalized MoS_2_ for rapid detection of avian coronavirus on cotton thread. IEEE Sens J. 2018;18(11):4358‐4363. 32390783 10.1109/JSEN.2018.2829084 PMC7186039

[342] Karnaushenko D , Ibarlucea B , Lee S , et al. Light weight and flexible high‐performance diagnostic platform. Adv Healthcare Mater. 2015;4(10):1517‐1525. 10.1002/adhm.201500128 25946521

[343] Lee DW , Lee J , Sohn IY , et al. Field‐effect transistor with a chemically synthesized MoS2 sensing channel for label‐free and highly sensitive electrical detection of DNA hybridization. Nano Res. 2015;8(7):2340‐2350.

[344] Qin Z , Peng R , Baravik IK , Liu X . Fighting COVID‐19: integrated micro‐ and nanosystems for viral infection diagnostics. Matter. 2020;3(3):628‐651. 32838297 10.1016/j.matt.2020.06.015 PMC7346839

[345] Miripour ZS , Sarrami‐Forooshani R , Sanati H , et al. Real‐time diagnosis of reactive oxygen species (ROS) in fresh sputum by electrochemical tracing; correlation between COVID‐19 and viral‐induced ROS in lung/respiratory epithelium during this pandemic. Biosens Bioelectron. 2020;165:112435. 32729548 10.1016/j.bios.2020.112435 PMC7341050

[346] Zhao Z , Cui H , Song W , Ru X , Zhou W , Yu X. A simple magnetic nanoparticles‐based viral RNA extraction method for efficient detection of SARS‐CoV‐2. bioRxiv. Published online February 27, 2020. 10.1101/2020.02.22.961268

[347] Algaissi A , Agrawal AS , Hashem AM , Tseng CK . Quantification of the Middle East respiratory syndrome‐coronavirus RNA in tissues by quantitative real‐time RT‐PCR. Methods Mol Biol. 2020;2099:99‐106. 31883090 10.1007/978-1-0716-0211-9_8 PMC7122982

[348] Liu R , Han H , Liu F , et al. Positive rate of RT‐PCR detection of SARS‐CoV‐2 infection in 4880 cases from one hospital in Wuhan, China, from Jan to Feb 2020. Clin Chim Acta. 2020;505:172‐175. 32156607 10.1016/j.cca.2020.03.009 PMC7094385

[349] Chacón‐Torres JC , Reinoso C , Navas‐León DG , Briceño S , González G . Optimized and scalable synthesis of magnetic nanoparticles for RNA extraction in response to developing countries’ needs in the detection and control of SARS‐CoV‐2. Sci Rep. 2020;10:19004. 33149153 10.1038/s41598-020-75798-9 PMC7642403

[350] Somvanshi SB , Kharat PB , Saraf TS , Somwanshi SB , Shejul SB , Jadhav KM . Multifunctional nano‐magnetic particles assisted viral RNA‐extraction protocol for potential detection of COVID‐19. Mater Res Innov. 2020;1‐6.

[351] Knepp JH , Geahr MA , Forman MS , Valsamakis A . Comparison of automated and manual nucleic acid extraction methods for detection of enterovirus RNA. J Clin Microbiol. 2003;41(8):3532‐3536. 12904351 10.1128/JCM.41.8.3532-3536.2003 PMC179781

[352] Lee AHF , Gessert SF , Chen Y , Sergeev NV , Haghiri B . Preparation of iron oxide silica particles for Zika viral RNA extraction. Heliyon. 2018;4(3):e00572. 10.1016/j.heliyon.2018.e00572 PMC5854921 29556569

[353] Tang Y , Anne Hapip C , Liu B , Fang CT . Highly sensitive TaqMan RT‐PCR assay for detection and quantification of both lineages of West Nile virus RNA. J Clin Virol. 2006;36(3):177‐182. 16675298 10.1016/j.jcv.2006.02.008

[354] Chianese‐Bullock KA , Irvin WP Jr , Petroni GR , et al. A multipeptide vaccine is safe and elicits T‐cell responses in participants with advanced stage ovarian cancer. J Immunother. 2008;31(4):420‐430. 18391753 10.1097/CJI.0b013e31816dad10

[355] Koellhoffer JF , Higgins CD , Lai JR . Protein engineering strategies for the development of viral vaccines and immunotherapeutics. FEBS Lett. 2014;588(2):298‐307. 24157357 10.1016/j.febslet.2013.10.014 PMC3947166

[356] Skwarczynski M , Toth I . Peptide‐based synthetic vaccines. Chem Sci. 2016;7(2):842‐854. 28791117 10.1039/c5sc03892h PMC5529997

[357] Gartner TE , Jayaraman A . Modeling and simulations of polymers: a roadmap. Macromolecules. 2019;52(3):755‐786.

[358] McNeil SE . Unique benefits of nanotechnology to drug delivery and diagnostics. Methods Mol Biol. 2011;697:3‐8. 21116949 10.1007/978-1-60327-198-1_1

[359] Petros RA , DeSimone JM . Strategies in the design of nanoparticles for therapeutic applications. Nat Rev Drug Discov. 2010;9(8):615‐627. 20616808 10.1038/nrd2591

[360] Chauhan G , Madou MJ , Kalra S , Chopra V , Ghosh D , Martinez‐Chapa SO . Nanotechnology for COVID‐19: therapeutics and vaccine research. ACS Nano. 2020;14(7):7760‐7782. 32571007 10.1021/acsnano.0c04006

[361] Yang ZY , Kong WP , Huang Y , et al. A DNA vaccine induces SARS coronavirus neutralization and protective immunity in mice. Nature. 2004;428(6982):561‐564. 15024391 10.1038/nature02463 PMC7095382

[362] Graham RL , Becker MM , Eckerle LD , Bolles M , Denison MR , Baric RS . A live, impaired‐fidelity coronavirus vaccine protects in an aged, immunocompromised mouse model of lethal disease. Nat Med. 2012;18(12):1820‐1826. 23142821 10.1038/nm.2972 PMC3518599

[363] Banchereau J , Steinman RM . Dendritic cells and the control of immunity. Nature. 1998;392(6673):245‐252. 9521319 10.1038/32588

[364] Steinman RM , Banchereau J . Taking dendritic cells into medicine. Nature. 2007;449:419‐426. 17898760 10.1038/nature06175

[365] Joffre OP , Segura E , Savina A , Amigorena S . Cross‐presentation by dendritic cells. Nat Rev Immunol. 2012;12:557‐569. 22790179 10.1038/nri3254

[366] Smith DM , Simon JK , Baker JR Jr. Applications of nanotechnology for immunology. Nat Rev Immunol. 2013;13(8):592‐605. 23883969 10.1038/nri3488 PMC7097370

[367] Staroverov SA , Vidyasheva IV , Gabalov KP , Vasilenko OA , Laskavyi VN , Dykman LA . Immunostimulatory effect of gold nanoparticles conjugated with transmissible gastroenteritis virus. Bull Exp Biol Med. 2011;151(4):436. 22448360 10.1007/s10517-011-1350-8 PMC7087599

[368] Sekimukai H , Iwata‐Yoshikawa N , Fukushi S , et al. Gold nanoparticle‐adjuvanted S protein induces a strong antigen‐specific IgG response against severe acute respiratory syndrome‐related coronavirus infection, but fails to induce protective antibodies and limit eosinophilic infiltration in lungs. Microbiol Immunol. 2020;64(1):33‐51. 31692019 10.1111/1348-0421.12754 PMC7168429

[369] Kim YS , Son A , Kim J , et al. Chaperna‐mediated assembly of ferritin‐based Middle East respiratory syndrome‐coronavirus nanoparticles. Front Immunol. 2018;9:1093. 29868035 10.3389/fimmu.2018.01093 PMC5966535

[370] Jung SY , Kang KW , Lee EY , et al. Heterologous prime–boost vaccination with adenoviral vector and protein nanoparticles induces both Th1 and Th2 responses against Middle East respiratory syndrome coronavirus. Vaccine. 2018;36(24):3468‐3476. 29739720 10.1016/j.vaccine.2018.04.082 PMC7115429

[371] Lin LC , Huang C , Yao B , et al. Viromimetic STING agonist‐loaded hollow polymeric nanoparticles for safe and effective vaccination against Middle East respiratory syndrome coronavirus. Adv Funct Mater. 2019;29(28):1807616. 32313544 10.1002/adfm.201807616 PMC7161765

[372] Krichel B , Falke S , Hilgenfeld R , Redecke L , Uetrecht C . Processing of the SARS‐CoV pp1a/ab nsp7‐10 region. Biochem J. 2020;477(5):1009‐1019. 32083638 10.1042/BCJ20200029 PMC7078746

[373] Mansoor F , Earley B , Cassidy JP , Markey B , Doherty S , Welsh MD . Comparing the immune response to a novel intranasal nanoparticle PLGA vaccine and a commercial BPI3V vaccine in dairy calves. BMC Vet Res. 2015;11:220. 26293453 10.1186/s12917-015-0481-y PMC4546173

[374] Dykman LA , Staroverov SA , Fomin AS . Effect of M2e peptide–gold nanoparticle conjugates on development of anti‐influenza antibodies. Gold Bull. 2018;51:197‐203.

[375] Cai M , Wang C , Li Y , et al. Virus‐like particle vaccine by intranasal vaccination elicits protective immunity against respiratory syncytial viral infection in mice. Acta Biochim Biophys Sin. 2017;49:74‐82. 27974288 10.1093/abbs/gmw118

[376] Łoczechin A , Séron K , Barras A , et al. Functional carbon quantum dots as medical countermeasures to human coronavirus. ACS Appl Mater Interfaces. 2019;11(46):42964‐42974. 31633330 10.1021/acsami.9b15032 PMC7075527

[377] Foronjy RF , Dabo AJ , Cummins N , Geraghty P . Leukemia inhibitory factor protects the lung during respiratory syncytial viral infection. BMC Immunol. 2014;15:41. 25277705 10.1186/s12865-014-0041-4 PMC4189665

[378] Quinton LJ , Mizgerd JP , Hilliard KL , Jones MR , Kwon CY , Allen E . Leukemia inhibitory factor signaling is required for lung protection during pneumonia. J Immunol. 2012;188(12):6300‐6308. 22581855 10.4049/jimmunol.1200256 PMC3370070

[379] Metcalfe SM , Strom TB , Williams A , Fahmy TM . Multiple sclerosis and the LIF/IL‐6 axis: use of nanotechnology to harness the tolerogenic and reparative properties of LIF. Nanobiomedicine. 2015;2:5. 29942371 10.5772/60622 PMC5997376

[380] Metcalfe SM . Mesenchymal stem cells and management of COVID‐19 pneumonia. Med Drug Discov. 2020;5:100019. 32296777 10.1016/j.medidd.2020.100019 PMC7147223

[381] Rinanda T . In silico studies in antimicrobial peptides design and development. IOP Conf Ser: Earth Environ Sci. 2019;305:012062.

[382] Quintero‐Gil C , Parra‐Suescún J , Lopez‐Herrera A , Orduz S . In‐silico design and molecular docking evaluation of peptides derivatives from bacteriocins and porcine beta defensin‐2 as inhibitors of Hepatitis E virus capsid protein. Virus Dis. 2017;28(3):281‐238. 10.1007/s13337-017-0383-7 PMC5684997 29291214

[383] Kelley LA , Mezulis S , Yates CM , Wass MN , Sternberg MJ . The Phyre2 web portal for protein modeling, prediction and analysis. Nat Protoc. 2015;10(6):845‐858. 25950237 10.1038/nprot.2015.053 PMC5298202

[384] McGuffin LJ , Bryson K , Jones DT . The PSIPRED protein structure prediction server. Bioinformatics. 2000;16(4):404‐405. 10869041 10.1093/bioinformatics/16.4.404

[385] Yang J , Yan R , Roy A , Xu D , Poisson J , Zhang Y . The I‐TASSER suite: protein structure and function prediction. Nat Methods. 2015;12(1):7‐8. 25549265 10.1038/nmeth.3213 PMC4428668

[386] Lamiable A , Thévenet P , Rey J , Vavrusa M , Derreumaux P , Tufféry P . PEP‐FOLD3: faster de novo structure prediction for linear peptides in solution and in complex. Nucleic Acids Res. 2016;44(W1):W449‐W454. 27131374 10.1093/nar/gkw329 PMC4987898

[387] Schwede T , Kopp J , Guex N , Peitsch MC . SWISS‐MODEL: an automated protein homology‐modeling server. Nucleic Acids Res. 2003;31(13):3381‐3385. 12824332 10.1093/nar/gkg520 PMC168927

[388] Corman VM , Landt O , Kaiser M , et al. Detection of 2019 novel coronavirus (2019‐nCoV) by real‐time RT‐PCR. Euro Surveill. 2020;25(3):2000045. 10.2807/1560-7917.ES.2020.25.3.2000045 PMC6988269 31992387

[389] Chu DKW , Pan Y , Cheng SMS , et al. Molecular diagnosis of a novel coronavirus (2019‐nCoV) causing an outbreak of pneumonia. Clin Chem. 2020;66(4):549‐555. 32031583 10.1093/clinchem/hvaa029 PMC7108203

[390] Marston DA , McElhinney LM , Ellis RJ , et al. Next generation sequencing of viral RNA genomes. BMC Genomics. 2013;14(1):444. 23822119 10.1186/1471-2164-14-444 PMC3708773

[391] Lopez‐Rincon A , Tonda A , Mendoza‐Maldonado L , et al. Classification and specific primer design for accurate detection of SARS‐CoV‐2 using deep learning. Sci Rep. 2021;11(1):947. 33441822 10.1038/s41598-020-80363-5 PMC7806918

[392] Pearson WR . Rapid and sensitive sequence comparison with FASTP and FASTA. Methods Enzymol. 1990;183:63‐98. 2156132 10.1016/0076-6879(90)83007-v

[393] Altschul SF , Gish W , Miller W , Myers EW , Lipman DJ . Basic local alignment search tool. J Mol Biol. 1990;215(3):403‐410. 2231712 10.1016/S0022-2836(05)80360-2

[394] Chenna R , Sugawara H , Koike T , et al. Multiple sequence alignment with the clustal series of programs. Nucleic Acids Res. 2003;31(13):3497‐3500. 12824352 10.1093/nar/gkg500 PMC168907

[395] Notredame C , Higgins DG , Heringa J . T‐Coffee: a novel method for fast and accurate multiple sequence alignment. J Mol Biol. 2000;302(1):205‐217. 10964570 10.1006/jmbi.2000.4042

[396] LeCun Y , Bengio Y , Hinton G . Deep learning. Nature. 2015;521(7553):436‐444. 26017442 10.1038/nature14539

[397] Li Y , Huang C , Ding L , Li Z , Pan Y , Gao X . Deep learning in bioinformatics: introduction, application, and perspective in the big data era. Methods. 2019;166:4‐21. 31022451 10.1016/j.ymeth.2019.04.008

[398] Schmidhuber J . Deep learning in neural networks: an overview. Neural Netw. 2015;61:85‐117. 25462637 10.1016/j.neunet.2014.09.003

[399] Esteva A , Robicquet A , Ramsundar B , et al. A guide to deep learning in healthcare. Nat Med. 2019;25(1):24‐29. 30617335 10.1038/s41591-018-0316-z

[400] Nguyen NG , Tran VA , Ngo DL , et al. DNA sequence classification by convolutional neural network. J Biomed Sci Eng. 2016;9(05):280.

[401] Rizzo R , Fiannaca A , La Rosa M , Urso A . A deep learning approach to DNA sequence classification. In: International Meeting on Computational Intelligence Methods for Bioinformatics and Biostatistics. Springer; 2015;9874:129‐140.

[402] Panwar H , Gupta PK , Siddiqui MK , Morales‐Menendez R , Singh V . Application of deep learning for fast detection of COVID‐19 in X‐rays using nCOVnet. Chaos Solitons Fractals. 2020;138:109944. 32536759 10.1016/j.chaos.2020.109944 PMC7254021

[403] Enayatkhani M , Hasaniazad M , Faezi S , et al. Reverse vaccinology approach to design a novel multi‐epitope vaccine candidate against COVID‐19: an in silico study. J Biomol Struct Dyn. 2021;39(8):2857‐2872. 32295479 10.1080/07391102.2020.1756411 PMC7196925

[404] Dong R , Chu Z , Yu F , Zha Y . Contriving multi‐epitope subunit of vaccine for COVID‐19: immunoinformatics approaches. Front Immunol. 2020;11:1784. 32849643 10.3389/fimmu.2020.01784 PMC7399176

[405] Naz A , Shahid F , Butt TT , Awan FM , Ali A , Malik A . Designing multi‐epitope vaccines to combat emerging coronavirus disease 2019 (COVID‐19) by employing immuno‐informatics approach. Front Immunol. 2020;11:1663. 32754160 10.3389/fimmu.2020.01663 PMC7365865

[406] Bullock J , Pham KH , Lam CS , Luengo‐Oroz M . Mapping the landscape of artificial intelligence applications against COVID‐19. J Artif Intell Res. 2020;69:807‐845.

[407] Doytchinova IA , Flower DR . VaxiJen: a server for prediction of protective antigens, tumour antigens and subunit vaccines. BMC Bioinformatics. 2007;8:4. 17207271 10.1186/1471-2105-8-4 PMC1780059

[408] Wang P , Sidney J , Kim Y , et al. Peptide binding predictions for HLA DR, DP and DQ molecules. BMC Bioinformatics. 2010;11:568. 21092157 10.1186/1471-2105-11-568 PMC2998531

[409] Larsen MV , Lundegaard C , Lamberth K , Buus S , Lund O , Nielsen M . Large‐scale validation of methods for cytotoxic T‐lymphocyte epitope prediction. BMC Bioinformatics. 2007;8:424. 17973982 10.1186/1471-2105-8-424 PMC2194739

[410] Paul S , Sidney J , Sette A , Peters B . TepiTool: a pipeline for computational prediction of T cell epitope candidates. Curr Protoc Immunol. 2016;114:18.19.1‐18.19.24. 27479659 10.1002/cpim.12 PMC4981331

[411] Feldhahn M , Thiel P , Schuler MM , et al. EpiToolKit–a web server for computational immunomics. Nucleic Acids Res. 2008;36:W519‐W522. 18440979 10.1093/nar/gkn229 PMC2447732

[412] Saha S , Raghava GP . Prediction of continuous B‐cell epitopes in an antigen using recurrent neural network. Proteins. 2006;65(1):40‐48. 16894596 10.1002/prot.21078

[413] Tong JC , Tan TW , Ranganathan S . Methods and protocols for prediction of immunogenic epitopes. Brief Bioinform. 2007;8(2):96‐108. 17077136 10.1093/bib/bbl038

[414] Jespersen MC , Peters B , Nielsen M , Marcatili P . BepiPred‐2.0: improving sequence‐based B‐cell epitope prediction using conformational epitopes. Nucleic Acids Res. 2017;45(W1):W24‐W29. 28472356 10.1093/nar/gkx346 PMC5570230

[415] Ponomarenko J , Bui HH , Li W , et al. ElliPro: a new structure‐based tool for the prediction of antibody epitopes. BMC Bioinformatics. 2008;9(1):514. 19055730 10.1186/1471-2105-9-514 PMC2607291

[416] Saha S , Raghava GP . AlgPred: prediction of allergenic proteins and mapping of IgE epitopes. Nucleic Acids Res. 2006;34:W202‐W209. 16844994 10.1093/nar/gkl343 PMC1538830

[417] Dimitrov I , Bangov I , Flower DR , Doytchinova I . AllerTOP v.2–a server for in silico prediction of allergens. J Mol Model. 2014;20(6):2278. 24878803 10.1007/s00894-014-2278-5

[418] Magnan CN , Zeller M , Kayala MA , et al. High‐throughput prediction of protein antigenicity using protein microarray data. Bioinformatics. 2010;26(23):2936‐2943. 20934990 10.1093/bioinformatics/btq551 PMC2982151

[419] Magnan CN , Randall A , Baldi P . SOLpro: accurate sequence‐based prediction of protein solubility. Bioinformatics. 2009;25(17):2200‐2207. 19549632 10.1093/bioinformatics/btp386

[420] Castiglione F , Bernaschi M . C‐immsim: playing with the immune response. In Proceedings of the 16th International Symposium on Mathematical Theory of Networks and Systems (MTNS2004). 2004.

[421] Kelley LA , Sternberg MJ . Protein structure prediction on the web: a case study using the Phyre server. Nat Protoc. 2009;4(3):363‐371. 19247286 10.1038/nprot.2009.2

[422] Heo L , Park H , Seok C . GalaxyRefine: protein structure refinement driven by side‐chain repacking. Nucleic Acids Res. 2013;41:W384‐W388. 23737448 10.1093/nar/gkt458 PMC3692086

[423] Madanchi H , Khalaj V , Jang S , et al. AurH1: a new heptapeptide derived from Aurein1.2 antimicrobial peptide with specific and exclusive fungicidal activity. J Pep Sci. 2019;25:e3175. 10.1002/psc.3175 31264322

[424] Wang W , Xia M , Chen J , et al. Data set for phylogenetic tree and RAMPAGE Ramachandran plot analysis of SODs in *Gossypium raimondii* and *G. arboreum* . Data Brief. 2016;9:345‐348. 27672674 10.1016/j.dib.2016.05.025 PMC5030311

[425] Wiederstein M , Sippl MJ . ProSA‐web: interactive web service for the recognition of errors in three‐dimensional structures of proteins. Nucleic Acids Res. 2007;35:W407‐W410. 17517781 10.1093/nar/gkm290 PMC1933241

[426] Ikram A , Zaheer T , Awan FM , et al. Exploring NS3/4A, NS5A and NS5B proteins to design conserved subunit multi‐epitope vaccine against HCV utilizing immunoinformatics approaches. Sci Rep. 2018;8(1):1‐4. 30382118 10.1038/s41598-018-34254-5 PMC6208421

[427] Bhattacharya M , Sharma AR , Patra P , et al. A SARS‐CoV‐2 vaccine candidate: in‐silico cloning and validation. Inform Med Unlocked. 2020;20:100394. 32835079 10.1016/j.imu.2020.100394 PMC7361115

[428] Dominguez C , Boelens R , Bonvin AM . HADDOCK: a protein–protein docking approach based on biochemical or biophysical information. J Am Chem Soc. 2003;125(7):1731‐1737. 12580598 10.1021/ja026939x

[429] Kozakov D , Hall DR , Xia B , et al. The ClusPro web server for protein–protein docking. Nat Protoc. 2017;12(2):255‐278. 28079879 10.1038/nprot.2016.169 PMC5540229

[430] Comeau SR , Gatchell DW , Vajda S , Camacho CJ . ClusPro: a fully automated algorithm for protein–protein docking. Nucleic Acids Res. 2004;32:W96‐W99. 15215358 10.1093/nar/gkh354 PMC441492

[431] Lindahlv E , Hess B , van der Spoel D . GROMACS 3.0: a package for molecular simulation and trajectory analysis. J Mol Model. 2001;7:306‐317.

[432] Krieger E , Koraimann G , Vriend G . Increasing the precision of comparative models with YASARA NOVA–a self‐parameterizing force field. Proteins. 2002;47(3):393‐402. 11948792 10.1002/prot.10104

[433] Misra N , Panda PK , Shah K , Sukla LB , Chaubey P . Population coverage analysis of T‐cell epitopes of *Neisseria meningitidis* serogroup B from iron acquisition proteins for vaccine design. Bioinformation. 2011;6(7):255. 21738325 10.6026/97320630006255 PMC3124689

[434] Yadav M , Dhagat S , Eswari JS . Emerging strategies on in silico drug development against COVID‐19: challenges and opportunities. Eur J Pharmaceut Sci. 2020;155:105522. 10.1016/j.ejps.2020.105522 PMC7438372 32827661

[435] Durojaiye AB , Clarke JD , Stamatiades GA , Wang C . Repurposing cefuroxime for treatment of COVID‐19: a scoping review of in silico studies. J Biomol Struct Dyn. 2021;39(12):4547‐4554. 32538276 10.1080/07391102.2020.1777904 PMC7298880

[436] Wang J . Fast identification of possible drug treatment of coronavirus disease‐19 (COVID‐19) through computational drug repurposing study. J Chem Inf Model. 2020;60:3277‐3286. 32315171 10.1021/acs.jcim.0c00179 PMC7197972

[437] Ciliberto G , Cardone L . Boosting the arsenal against COVID‐19 through computational drug repurposing. Drug Discov Today. 2020;25:946‐948. 32304645 10.1016/j.drudis.2020.04.005 PMC7158822

[438] Amin SA , Ghosh K , Gayen S , Jha T . Chemical‐informatics approach to COVID‐19 drug discovery: Monte Carlo based QSAR, virtual screening and molecular docking study of some in‐house molecules as papain‐like protease (PLpro) inhibitors. J Biomol Struct Dyn. 2021;39(13):4764‐4773. 32568618 10.1080/07391102.2020.1780946 PMC7332872

[439] Xu C , Ke Z , Liu C , et al. Systemic in silico screening in drug discovery for coronavirus disease (COVID‐19) with an online interactive web server. J Chem Inf Model. 2020;60(12):5735‐5745. 32786695 10.1021/acs.jcim.0c00821

[440] Keshavarzi Arshadi A , Webb J , Salem M , et al. Artificial intelligence for COVID‐19 drug discovery and vaccine development. Front Artif Intell. 2020;3:65. 33733182 10.3389/frai.2020.00065 PMC7861281

[441] Arora G , Joshi J , Mandal RS , Shrivastava N , Virmani R , Sethi T . Artificial intelligence in surveillance, diagnosis, drug discovery and vaccine development against COVID‐19. Pathogens. 2021;10(8):1048. 34451513 10.3390/pathogens10081048 PMC8399076

[442] Trott O , Olson AJ . Autodock vina: improving the speed and accuracy of docking with a new scoring function, efficient optimization, and multithreading. J Comput Chem. 2010;31(2):455‐461. 19499576 10.1002/jcc.21334 PMC3041641

[443] Lo YC , Rensi SE , Torng W , Altman RB . Machine learning in chemoinformatics and drug discovery. Drug Discov Today. 2018;23(8):1538‐1546. 29750902 10.1016/j.drudis.2018.05.010 PMC6078794

[444] Cherkasov A , Muratov EN , Fourches D , et al. QSAR modeling: where have you been? Where are you going to? J Med Chem. 2014;57(12):4977‐5010. 24351051 10.1021/jm4004285 PMC4074254

