# Supplementary material for: COVID‐19: A systematic review and update on prevention, diagnosis, and treatment
Source: MedComm (2020). 2022 Feb 17;3(1):e115. doi: 10.1002/mco2.115 (PMC8906461; doi:10.1002/mco2.115)
Supplement: Supplementary file 1 — SUPPORTING INFORMATION [file MCO2-3-0-s001.docx]

**COVID-19: A Systematic Review and Update on Prevention, Diagnosis, and Treatment**

Hooman Aghamirza Moghim Aliabadi^1,2^, Reza Eivazzadeh-Keihan^3^, Arezoo Beig Parikhani^4^, Sara Fattahi Mehraban^5^, Ali Maleki^3^, Sepideh Fereshteh^6^, Masoume Bazaz^4^, Ashkan Zolriasatein^5^, Bahareh Bozorgnia^7^, Saman Rahmati^4^, Fatemeh Saberi^8^, Zeinab Yousefi^9,10^, Shadi Damough^4^, Sara Mohseni^5^, Hamid Salehzadeh^11^, Vahid Khakyzadeh^12,*^, Hamid Madanchi^13,14,*^, Gholam Ali Kardar^7^, Payam Zarrintaj^15^, Mohammad Reza Saeb^16^, Masoud Mozafari^17,*^

^1^ Protein Chemistry Laboratory, Department of Medical Biotechnology, Biotechnology Research Center, Pasteur Institute of Iran, Tehran, Iran

^2^ Advance Chemical Studies Laboratory, Faculty of Chemistry, K.N. Toosi University, Tehran, Iran

^3^ Department of Chemistry, Iran University of Science and Technology, Tehran, Iran

^4^ Department of Medical Biotechnology, Biotechnology Research Center, Pasteur Institute, Tehran, Iran

^5^ Non-metallic Materials Research Group, Niroo Research Institute, Tehran, Iran

^6^ Department of Bacteriology, Pasteur Institute of Iran, Tehran

^7^ Faculty of Chemistry, Alzahra University, Tehran, Iran

^8^ Department of Medical Biotechnology, School of Advanced Technologies in Medicine, Shahid Beheshti University of Medical Sciences, Tehran, Iran

^9^ Department of Medical Biotechnology, School of Advanced Technologies in Medicine, Tehran University of Medical Sciences, Tehran, Iran

^10^ Immunology, Asthma & Allergy Research Institute, Tehran University of Medical Sciences, Tehran, Iran

^11^ Faculty of Chemistry, Kharazmi University, Tehran, Iran

^12^ Department of Chemistry, K. N. Toosi University of Technology, Tehran, Iran

^13^ School of Medicine, Semnan University of Medical Sciences, Semnan, Iran

^14^ Drug Design and Bioinformatics Unit, Department of Medical Biotechnology, Biotechnology Research Center, Pasteur Institute of Iran, Tehran, Iran

^15^School of Chemical Engineering, Oklahoma State University, 420 Engineering North, Stillwater, OK, 74078, USA

^16^Université de Lorraine, CentraleSupélec, LMOPS, F-57000, Metz, France

^17^Faculty of Advanced Technologies in Medicine, Department of Tissue Engineering & Regenerative Medicine, Iran University of Medical Sciences, Tehran, Iran

**Corresponding Authors:**

* V. Khakyzadeh, PhD; E-mail: [v.khakyzadeh@kntu.ac.ir](mailto:v.khakyzadeh@kntu.ac.ir)

* H. Madanchi, PhD; E-mail: [hamid.madanchi@yahoo.com](mailto:hamid.madanchi@yahoo.com)

* M. Mozafari, PhD; Currently at: Lunenfeld-Tanenbaum Research Institute, Mount Sinai Hospital, University of Toronto, Toronto, Canada. E-mail: [mozafari.masoud@gmail.com](mailto:mozafari.masoud@gmail.com); [m.mozafari@utoronto.ca](mailto:m.mozafari@utoronto.ca)

**Supplementary information**

**Table S1.** Therapeutic drugs against COVID-19 with their chemical structure

| Drug Name | | Chemical Structure |
| --- | --- | --- |
| Remdesivir | |  |
| GS-441524 | |  |
| Chloroquine | |  |
| Hydroxychloroquine | |  |
| Ivermectin  (two homologs) | 5-O-dimethyl-22,23-dihydroavermectin B_1a_ |  |
|  | 5-O-dimethyl-22,23-dihydroavermectin B_1b_ |  |
| Cyclosporine | |  |
| Alisporivir | |  |
| Sofosbuvir | |  |
| Lopinavir | |  |
| Ritonavir | |  |
| Favipiravir | |  |
| Azithromycin | |  |

**Table S2.** Link address of most important servers

| Server | Link |
| --- | --- |
| NCBI | [www.ncbi.nlm.nih.gov](file:///C:\Users\h_aghamirza\Desktop\Hooman\Corona\www.ncbi.nlm.nih.gov) |
| RCSB PDB | [www.rcsb.org](http://www.rcsb.org/) |
| Expasy | [www.expasy.org](file:///C:\Users\h_aghamirza\Desktop\Hooman\Corona\www.expasy.org) |
| EBI-EMBL and PDB sum | [www.ebi.ac.uk](file:///C:\Users\h_aghamirza\Desktop\Hooman\Corona\www.ebi.ac.uk) |
